# Gut microbiota in health and disease

**DOI:** 10.1186/s43556-026-00512-6

**Published:** 2026-07-20

**Authors:** Yangziyi Li, Xueyan Xiao, Xin Jiang, Xinyi Li, Weijun Wang, Rong Lin

**Affiliations:** 1 Department of Gastroenterology, Union Hospital, Tongji Medical College, Huazhong University of Science and Technology, 1277 Jiefang Avenue, Wuhan, 430022 China; 2 Hubei Province Engineering Research Center for Minimally Invasive Therapy of Digestive Disorders, Union Hospital, Tongji Medical College, Huazhong University of Science and Technology, Wuhan, Hubei China

**Keywords:** Gut microbiota, Dysbiosis, Colorectal cancer (CRC), Gut-organ axis, Microbiota-based biomarker, Microbiota-based therapy

## Abstract

The gut microbiota is integral to host physiology, contributing to metabolic homeostasis, epithelial barrier integrity, immune balance, and bidirectional communication along gut–organ axes. Disruption of this ecosystem, commonly referred to as dysbiosis, is increasingly implicated in a wide range of gastrointestinal and extra-intestinal diseases. Rather than reflecting isolated compositional changes, microbiota-related pathology often involves interconnected disturbances in barrier function, microbial metabolism, immune regulation, genotoxicity, inflammatory and oncogenic signaling, and long-range communication with distal organs. However, key challenges remain, particularly in resolving causality, accounting for interindividual heterogeneity, and translating complex microbiome data into robust clinical tools. In this review, we summarize the role of the gut microbiota in maintaining host homeostasis and outline the concept, drivers, and consequences of dysbiosis. We then discuss the major mechanisms through which the gut microbiota contributes to disease development and progression, using colorectal cancer as a representative gastrointestinal example and gut–organ axes as a framework for extra-intestinal disorders. We further highlight current translational advances in microbiota-based biomarkers, dietary modulation, biotic and postbiotic strategies, fecal microbiota transplantation, and emerging precision microbiota therapies. By integrating mechanistic insights with translational perspectives, this review offers an updated framework for interpreting the gut microbiota in health and disease and may help inform the future development of more precise, mechanism-informed diagnostic and therapeutic strategies.

## Introduction

The gut microbiota is a complex microbial ecosystem inhabiting the human gastrointestinal tract and maintaining dynamic interactions with the host [1, 2]. It regulates diverse physiological processes, including nutrient metabolism, epithelial integrity, immune function, and systemic signaling [1, 2]. With the rapid development of high-throughput sequencing, multi-omics profiling, and functional experimental approaches, our understanding of how the gut microbiota shapes both local intestinal homeostasis and systemic physiology has substantially expanded [2–4].

Increasing evidence links gut microbiota dysbiosis to numerous disorders, including gastrointestinal diseases such as colorectal cancer (CRC), inflammatory bowel disease (IBD), and irritable bowel syndrome (IBS), as well as extra-intestinal conditions affecting the liver, metabolism, nervous, and endocrine systems [1, 2]. The relationship between the gut microbiota and disease is complex and often bidirectional. A healthy gut microbiota cannot be defined by a single compositional pattern [5, 6], while dysbiosis may function as either a driver or consequence of disease [7, 8]. Moreover, the effects of specific microbes and metabolites are highly context-dependent, shaped by host background, environmental factors, and disease stage [9, 10].

These complexities present a major challenge for the field. Although many studies have identified disease-associated microbial signatures, compositional changes alone are often insufficient to explain pathogenesis or to support their reliable clinical translation. As a result, increasing attention has shifted toward functional mechanisms, including metabolic remodeling, barrier disruption, immune reprogramming, genotoxic damage, and inter-organ communication through gut–organ axes [1, 2]. These mechanistic advances have also increased interest in the clinical applicability of microbiota-based biomarkers and targeted interventions [11, 12].

Because host–microbiota interactions are complex and context-dependent, disease-associated microbial changes cannot be fully interpreted through composition alone. This review integrates current evidence on microbial homeostasis, dysbiosis, disease mechanisms, and translational applications, with an emphasis on how mechanistic insights can inform microbiota-based biomarkers and therapeutic strategies.

## Gut microbiota and health

A healthy gut microbiota is not simply defined by a fixed microbial composition, but rather by its ability to sustain host homeostasis across changing physiological and environmental conditions. Its key ecological features, including diversity, stability, and resilience, help maintain a functionally balanced microbial ecosystem. By mediating metabolic regulation, barrier protection, immune balance, and gut–organ axis communication, this ecosystem supports both intestinal and systemic health, making microbial homeostasis an important basis of host physiology.

### Definition of a healthy gut microbiota

**Fig. 1:**
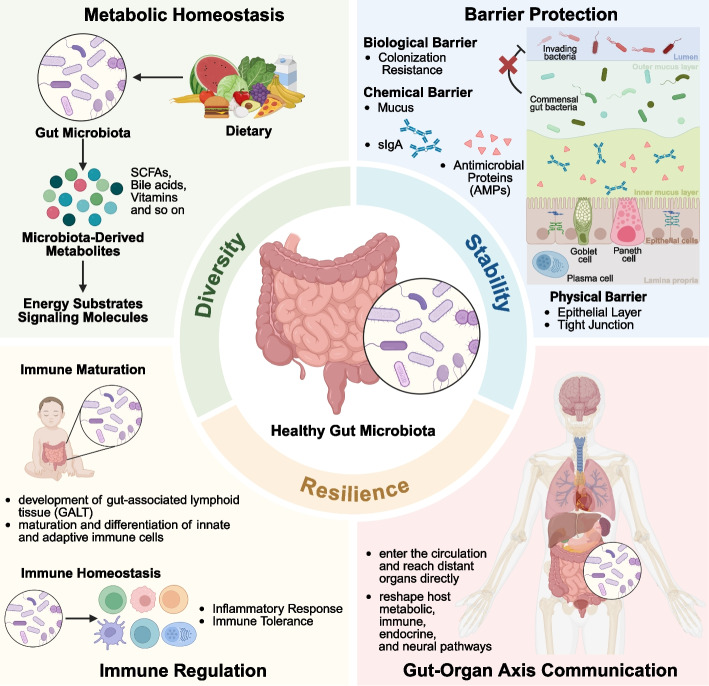
Healthy gut microbiota and host homeostasis. A healthy gut microbiota is generally characterized by diversity, stability, and resilience, and contributes to host homeostasis through metabolic homeostasis, barrier protection, immune regulation, and gut–organ axis communication

The gut microbiota is a complex microbial community colonizing the human gastrointestinal tract, where it establishes a relatively stable symbiotic relationship with the host [1, 3]. Bacteria, commonly dominated by *Firmicutes*, *Bacteroidetes*, and *Actinobacteria* [13], predominate in the gut microbiota, although fungi, viruses, and archaea are also present [3, 5, 14]. By contrast, the gut microbiome refers more broadly to this microbial community together with its collective genetic material and microbially derived metabolites and functional molecules [3, 15, 16]. Through sustained host–microbe interactions, the gut microbiota exerts broad regulatory effects on host physiological homeostasis and health. Its composition and function are shaped by multiple host-related and environmental factors, including genetics, diet, age, lifestyle, medication exposure, external environment and so on, which together drive substantial interindividual variability [1, 3, 5]. Nevertheless, a healthy gut microbiota is commonly characterized by relatively high diversity, overall stability, and resilience to perturbations [1, 5, 17] (Fig. 1). Rather than representing a fixed taxonomic configuration, a healthy gut microbiota is better understood as a functionally balanced ecological state. Gut microbiota diversity extends beyond taxonomic composition to functional diversity, enabling a wide range of activities in nutrient processing, metabolism, and immune modulation [5, 17]. Notably, many key functions can be maintained by multiple microbial taxa, a phenomenon known as functional redundancy [18, 19]. This allows functional compensation across alternative community structures and thereby promotes stability and resilience. Stability describes a dynamic equilibrium in core community functions under daily fluctuations [17, 20], while resilience refers to the ability to recover from stronger perturbations, such as antibiotics or inflammation [17, 20].

### Role of gut microbiota in maintaining host health

By producing bioactive metabolites, strengthening the intestinal barrier, modulating immune responses, and communicating along gut–organ axes, the gut microbiota supports metabolic homeostasis, barrier protection, and immune balance while extending its regulatory effects to distal organs [1, 3] (Fig. 1).

#### Metabolic homeostasis

There is extensive metabolic crosstalk between the gut microbiota and the host, mainly through two major mechanisms [21]. First, the microbiota acts as a metabolic organ that supplies energy substrates to the host. Second, microbiota-derived metabolites function as signaling molecules that regulate host metabolic homeostasis.

A substantial fraction of dietary components is not directly digested by the host. Instead, gut microbes ferment substrates such as dietary fiber, resistant starch, and other complex carbohydrates into host-accessible metabolites, particularly short-chain fatty acids (SCFAs) [21, 22]. More than 90% of SCFAs are absorbed by the intestinal epithelium via transporters such as solute carrier family 26 member 3 (SLC26A3) and monocarboxylate transporter 1 (MCT1) [22, 23]. Once absorbed, these metabolites may be used for lipid and carbohydrate synthesis or enter the tricarboxylic acid cycle, contributing about 5%–10% of daily energy requirements [22–24]. Among SCFAs, butyrate is the main fuel for colonocytes [25], whereas acetate and propionate primarily enter the portal circulation to support hepatic lipogenesis and cholesterol biosynthesis [26]. Acetate can also be detected in peripheral blood as a systemic energy substrate [27]. The gut microbiota broadens host metabolic capacity by contributing to vitamin synthesis, particularly vitamin K and B vitamins [28, 29], and by modulating amino acid metabolism [30].

Gut microbiota-derived metabolites also have regulatory roles beyond their use as substrates. Many of them engage host receptors, regulate hormone secretion, and influence epigenetic programs, thereby reshaping host metabolic networks. SCFAs and secondary bile acids provide well-established examples of this signaling role, as they connect microbial metabolism with host endocrine and metabolic regulation. For instance, SCFAs activate G protein-coupled receptors (GPCRs) such as GPR41 and GPR43, promoting the release of enteroendocrine hormones, including glucagon-like peptide-1 (GLP-1) and peptide YY (PYY), thereby improving insulin sensitivity, appetite regulation, and glycemic homeostasis [22, 31, 32]. Butyrate can also function as a histone deacetylase (HDAC) inhibitor and modulate transcriptional programs involved in lipid metabolism [22, 33]. Secondary bile acids signal through the nuclear receptor farnesoid X receptor (FXR) and Takeda G protein-coupled receptor 5 (TGR5) to regulate lipid absorption, hepatic lipid handling, and glucose homeostasis [34, 35]. More recent studies have expanded this view beyond classical SCFA and bile acid pathways, identifying additional microbiota-derived metabolites, such as 3-succinylcysteine [36] and mesaconate [37], as regulators of host metabolic homeostasis. Overall, the gut microbiota contributes to host metabolism not only by extracting nutrients, but also by producing signaling molecules that help coordinate metabolic regulation.

#### Barrier protection

The gut microbiota helps maintain intestinal barrier function at several levels. It supports the physical barrier formed by the epithelial layer and intercellular junctions, the chemical barrier composed of mucus and soluble defensive effectors, and the biological barrier provided by colonization resistance [38, 39]. Together, these mechanisms form an intestinal defense system against pathogen invasion and the translocation of harmful substances.

For the physical barrier, the gut microbiota restricts the translocation of luminal contents by promoting epithelial cell homeostasis and maintaining tight junction integrity [22, 38, 39]. Commensal bacteria and their metabolites provide energy substrates and regulatory signals that support epithelial metabolism and proliferation, thereby helping epithelial cells resist injury and repair damage [38–40]. Microbial signals also regulate the expression and function of tight junction proteins [38, 39]. For example, butyrate [22] and spermidine [41] can upregulate proteins such as Occludin, Claudin-1, and ZO-1, while specific strains such as *Bifidobacterium bifidum* [42] have also been shown to strengthen tight junction barrier function through microbe–host interactions. Together, these actions preserve epithelial integrity and limit increased intestinal permeability, often referred to as a “leaky gut”.

The gut microbiota also reinforces the chemical barrier through its effects on mucus, antimicrobial peptides (AMPs), and secretory IgA (sIgA) [38]. Microbial metabolites stimulate goblet cells to secrete mucin 2 (MUC2), which contributes to the formation of the double-layered mucus structure [43–45]. This structure supports commensal colonization while keeping the inner layer relatively sterile [43–45]. The microbiota also promotes AMP production by intestinal epithelial cells, including enterocytes, goblet cells, and Paneth cells. For example, microbial signals induce Paneth cells to secrete α-defensins and RegIIIγ, which help eliminate bacteria that penetrate the mucus layer [46, 47]. The microbiota further induces plasma cells to produce sIgA, which is transported into the intestinal lumen and limits bacterial adhesion to and invasion of the epithelium by coating microbial surfaces [48, 49].

The microbiota also provides biological antagonism through colonization resistance [50, 51]. A healthy gut microbiota suppresses potential pathogens by competing for limited nutrients, such as monosaccharides, amino acids, and iron, and by occupying attachment sites within the epithelial and mucus layers [52, 53]. In addition, commensal bacteria can directly inhibit invading pathogens by producing antimicrobial substances, including bacteriocins [54] and hydrogen peroxide [55]. Microbiota-derived metabolites, particularly SCFAs and secondary bile acids, also exert antimicrobial effects [22, 51, 56]. These metabolites can further restrict pathogen growth, especially that of environmentally sensitive pathogens, by lowering local pH [57] and oxygen levels [50] in the gut. Through these mechanisms, the microbiota helps maintain a community structure that resists pathogen overgrowth and supports long-term barrier stability.

#### Immune regulation

Beyond maintaining intestinal barrier integrity, the gut microbiota is also a key exogenous regulator of the immune system, shaping immune development and sustaining immune homeostasis [58].

The gut microbiota provides the initial driving force for immune maturation. Early-life colonization and subsequent continuous microbial exposure supply essential antigenic and signaling inputs that shape immune development via mechanisms such as microbe-associated molecular pattern (MAMP)–pattern recognition receptor (PRR) signaling, including Toll-like receptor (TLR)- and NOD-like receptor (NLR)-mediated pathways [59, 60]. On the one hand, the microbiota and its metabolites promote the development of gut-associated lymphoid tissue (GALT), including isolated lymphoid follicles and Peyer’s patches, thereby providing an organized niche for immune cell localization and crosstalk [61]. On the other hand, commensal microbes provide tonic signals that support the maturation and differentiation of both innate and adaptive immune cells [58]. For example, segmented filamentous bacteria (SFB) can induce T helper 17 (Th17) cell differentiation and thereby enhance host resistance to infection [62, 63]. In infants, *Bifidobacterium* can also promote CD4+ T cell polarization toward regulatory T (Treg) and Th1 lineages through indole-3-lactic acid (ILA), helping restrain mucosal inflammation [64]. Consistently, germ-free animal studies show that the absence of microbiota-driven immune education results in structural and functional immune defects, with lasting consequences for immune homeostasis and susceptibility to infectious and inflammatory diseases [58, 59].

In addition, the gut microbiota sustains immune homeostasis through dynamic regulatory networks that calibrate inflammatory intensity, effector programs, and response duration [58]. This allows the host to mount protective immunity when needed while limiting excessive inflammation and maintaining tolerance to commensals. Microbial metabolites are central mediators of this regulation. SCFAs promote the induction and stability of Treg cells through HDAC inhibition and/or GPCR-mediated signaling [22, 65, 66], and also modulate antigen-presenting and myeloid-cell function by reshaping cellular metabolism and cytokine production [67, 68], thereby restraining inflammatory amplification and promoting resolution. In parallel, microbiota-derived tryptophan metabolites regulate innate and adaptive effector pathways, including innate lymphoid cell 3 (ILC3)/Th17-IL (Interleukin)-22-linked responses, through activation of the aryl hydrocarbon receptor (AhR), promoting barrier restitution and coordinating immune responses [69, 70]. Secondary bile acids and their derivatives also regulate myeloid-cell inflammatory set-points and effector functions, such as chemotaxis and phagocytosis, through receptors including TGR5 and FXR [71–73]. Thus, microbiota-dependent immune regulation should not be viewed simply as immune activation or suppression, but as an integrated process that supports immune maturation, maintains tolerance, and calibrates inflammatory responses.

#### Gut–organ axis communication

The gut–organ axis refers to a bidirectional and dynamic communication network between the intestine and extraintestinal organs, mediated through multiple pathways, primarily including neural, endocrine, immune, and metabolic routes [74, 75]. This network enables intestinal states to modulate distal organ function while allowing distal organs to reciprocally influence gut physiology [74, 75]. Major gut–organ axes include the gut–liver, gut–brain, gut–kidney, gut–lung, gut–heart axes and so on. Through these axes, the gut microbiota functions not only as a local intestinal community, but also as a systemic regulator linking microbial ecology to distal organ physiology.

One major route involves the direct dissemination of gut microbes and their components, products, and metabolites through the circulation [74, 75]. SCFAs are representative examples. Along the gut–liver axis, butyrate supports hepatocyte metabolism [76], whereas propionate can suppress hepatic gluconeogenesis via GPR43, which contributes to glycemic homeostasis [32, 77]. In the gut–lung axis, SCFAs regulate pulmonary immune equilibrium by metabolically reprogramming alveolar macrophages [78]. In the gut–kidney axis, they participate in blood pressure regulation through receptor-mediated effects on renin secretion and vascular tone [79]. SCFAs can also influence blood–brain barrier function and microglial maturation, thereby supporting central nervous system homeostasis [80, 81].

Another route operates indirectly through the remodeling of host metabolic, immune, endocrine, and neural pathways [74, 75]. At the metabolic level, the microbiota can modulate carbohydrate, lipid, and protein metabolism across organs, as well as calcium absorption and vitamin metabolism, especially vitamins B, D, and K [82]. These effects are relevant to the gut–bone axis and bone remodeling [82]. Immunologically, the gut microbiota and its metabolites influence systemic immune homeostasis by affecting immune cells, including ILCs, Tregs, and Th17 cells, and related cytokines [58]. This has particular relevance for the respiratory tract as part of the mucosal immune system [83, 84]. In the endocrine system, the microbiota influences incretin secretion, including GLP-1, PYY, and glucose-dependent insulinotropic polypeptide (GIP), contributing to glycemic stability [85, 86]. The microbiota is also linked to sex hormone homeostasis. Microbial β-glucuronidases can deconjugate estrogens and affect circulating active estrogen levels [87, 88], while gut bacteria can convert tetrahydrodeoxycorticosterone (THDOC) into the progesterone derivative tetrahydroprogesterone (THP) through microbiota-dependent 21-dehydroxylation [89]. Through neural routes, the microbiota can signal through the vagus nerve or enteric nervous system and modulate neurotransmitter production, such as γ-aminobutyric acid (GABA) and serotonin, thereby influencing the nervous system and consequently other organs [90–92]. Taken together, these routes make gut–organ axis communication a link between local microbial activity and systemic homeostasis, and show how the gut microbiota helps coordinate intestinal and extraintestinal physiological functions.

## Gut microbiota dysbiosis

Gut microbiota dysbiosis is generally viewed as a dynamic disruption of the intestinal microbial ecosystem rather than a single compositional abnormality. It involves related changes in microbial structure, composition, function, and ecological stability, shaped by external exposures, host-related factors, and disease-associated feedback. These disturbances can impair barrier integrity, alter microbial metabolism, reshape immune regulation, and perturb gut–organ communication, thereby linking microbial imbalance to disease susceptibility and progression.

### Definition and features of gut microbiota dysbiosis

Gut microbiota dysbiosis refers to alterations in the structure, composition, and function of the intestinal microbial community, which can disrupt the symbiotic balance established through long-term co-evolution between the host and its microbes [93–95]. The concept extends beyond simple compositional imbalance to encompass functional perturbations, including shifts in microbial metabolite profiles and disturbances in the host–microbe interaction network [93–95]. Although some dysbiosis indices have been studied, substantial individual variability in the gut microbiota driven by factors such as host genetics, diet, and geography makes it difficult to define a single diagnostic criterion [96–98]. As a result, dysbiosis is better understood through an integrated assessment that combines diversity metrics, specific taxonomic changes, and functional parameters.

**Fig. 2:**
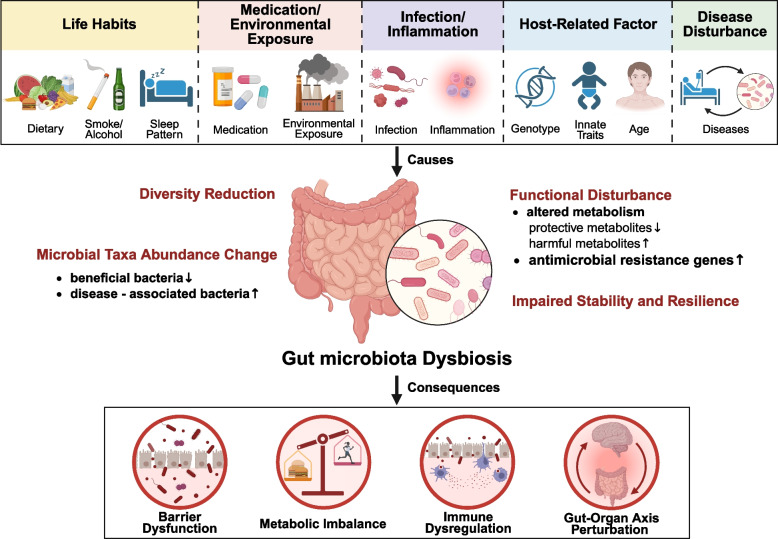
Gut microbiota dysbiosis. Gut microbiota dysbiosis can be driven by multiple factors, including lifestyle, medication and environmental exposure, infection and inflammation, host-related factors, and disease-associated disturbances. It is characterized by reduced diversity, altered microbial composition, functional disturbance, and impaired stability and resilience, ultimately leading to barrier dysfunction, metabolic imbalance, immune dysregulation, and perturbed gut–organ communication

Although there is no unified quantification standard, gut microbiota dysbiosis commonly manifests as a multidimensional disturbance involving microbial structure, function, and ecological stability (Fig. 2). The most prominent characteristic is a reduction in microbial α-diversity, which can be assessed using indices such as the Shannon diversity index [96, 99]. This is often accompanied by taxonomic imbalance, characterized by the depletion of beneficial commensals and the enrichment of disease-associated taxa. For example, SCFA-producing microbes, including *Faecalibacterium prausnitzii*, *Roseburia* spp., and *Eubacterium rectale*, are frequently reduced [99, 100], whereas disease-associated taxa such as *Fusobacterium nucleatum*, *Escherichia coli*, *Bacteroides fragilis*, *Enterococcus faecalis*, and *Streptococcus gallolyticus* show marked increases in abundance [101, 102]. However, these taxonomic changes should not be interpreted in isolation, as their biological relevance largely depends on the functional consequences they produce. Structurally altered microbial communities can lead to functional disturbances, particularly changes in microbial metabolic capacity. These include a decline in protective metabolites, especially SCFAs, and increased production of harmful microbial products, such as lipopolysaccharide (LPS) [21, 22]. Another feature of functional dysbiosis is the enrichment of antimicrobial resistance genes [103, 104]. Furthermore, dysbiosis is associated with reduced stability and resilience, which can lead to persistent or difficult-to-reverse imbalances in the microbiota, thereby increasing disease susceptibility [20, 99, 105].

### Causes and consequences of gut microbiota dysbiosis

Gut microbiota dysbiosis is rarely driven by a single cause, rather, it often emerges from interactions among external perturbations, host-intrinsic factors, and disease-related feedback loops [7, 94, 99] (Fig. 2). Lifestyle habits, such as diet, smoking, alcohol consumption, and sleep patterns, represent major exogenous contributors [94, 99, 106, 107]. For example, a Western diet high in fat and sugar but low in fiber has been associated with reduced microbial diversity and an expansion of *Proteobacteria* [108, 109]. Medications and environmental exposures are also key contributors [94, 110, 111]. Antibiotics can markedly reshape community composition [105, 112, 113], and early-life exposure to macrolides has been linked to long-lasting reductions in microbial diversity [114]. Several non-antibiotic drugs, such as proton pump inhibitors [115] and metformin [116], can also alter the gut microbiota [117]. Infection and inflammation serve as additional triggers [94, 118]. Pathogen invasion can directly disrupt community structure, while the ensuing inflammatory environment may further favor the outgrowth of certain pathobionts, thereby reinforcing and sustaining dysbiosis [94, 118]. In addition, host-related factors contribute substantially to dysbiosis. Genetic background and innate host traits can exert specific influences on microbiota composition [119], and advancing age is often accompanied by lower microbial diversity [120, 121]. Moreover, dysbiosis can act both as a “driver” of disease and as a “passenger” phenomenon. Disease-associated disturbances in metabolism, immunity, and other host processes can feed back to worsen microbial imbalance [122]. This bidirectional relationship suggests that dysbiosis is not merely an upstream disturbance, but may also be amplified by the disease environment itself.

The consequences of gut microbiota dysbiosis can be characterized as a synergistic disruption of barrier integrity, metabolic homeostasis, and immune regulation [3, 94] (Fig. 2). In terms of barrier function, dysbiosis is typically accompanied by impaired intestinal barrier integrity and diminished colonization resistance [38, 56]. This not only increases the risk of pathogen infection but also facilitates the translocation of harmful bacterial products, such as LPS, into the circulation through increased intestinal permeability, which can trigger systemic inflammatory responses [123, 124]. Metabolically, dysbiosis is associated with imbalances in key microbial pathways [125], including reduced SCFA availability, altered bile acid biotransformation, and disturbed tryptophan metabolism, alongside increased generation of deleterious metabolites such as the pro-atherogenic molecule trimethylamine N-oxide (TMAO) [126]. These alterations can impair the energy metabolism of intestinal epithelial cells and change the availability of specific signaling molecules [127, 128]. At the immune level, dysbiosis-driven shifts in microbial metabolites and products can reshape host immune responses, resulting in aberrant immune cell function and differentiation as well as activation of inflammatory pathways [129, 130]. The effects of dysbiosis can also extend beyond the intestine. Through metabolic, immune, endocrine, and neural pathways, dysbiosis can perturb gut–organ axis communication and thereby influence distal organs [3, 94, 131]. These barrier, metabolic, immune, and systemic alterations are closely interconnected, forming a self-reinforcing network through which dysbiosis can increase disease susceptibility and promote disease progression.

## Gut microbiota in disease development and progression

**Fig. 3:**
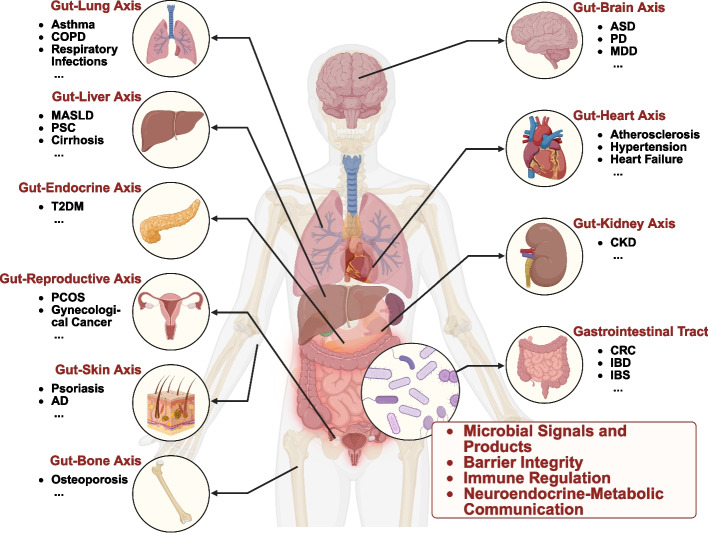
Gut microbiota in gastrointestinal and extra-intestinal diseases. Gut microbiota dysbiosis contributes to the development and progression of gastrointestinal diseases and multiple extra-intestinal disorders through microbial signals and products, barrier integrity, immune regulation, and neuroendocrine-metabolic communication across gut–organ axes

Although the causal direction between dysbiosis and disease often remains difficult to define, the gut microbiota is increasingly recognized as a key disease regulator, exerting both pathogenic and protective effects. Dysbiosis can promote pathology by disrupting barrier integrity, driving chronic inflammation and immune dysregulation, producing bioactive metabolites, modulating host signaling pathways, and, in some cases, generating genotoxins that directly damage host DNA. Conversely, commensal microbes can protect the host by regulating signaling, shaping the immune microenvironment, and producing beneficial metabolites. In the following sections, we first focus on gastrointestinal diseases, using colorectal cancer (CRC) as a representative example to illustrate major microbiota-related pathogenic and protective mechanisms, and then extend the discussion to gut–organ axes to summarize how the gut microbiota influences extra-intestinal diseases (Fig. 3).

### Gut microbiota in gastrointestinal diseases: colorectal cancer as a representative example

#### Colorectal Cancer (CRC)

**Fig. 4:**
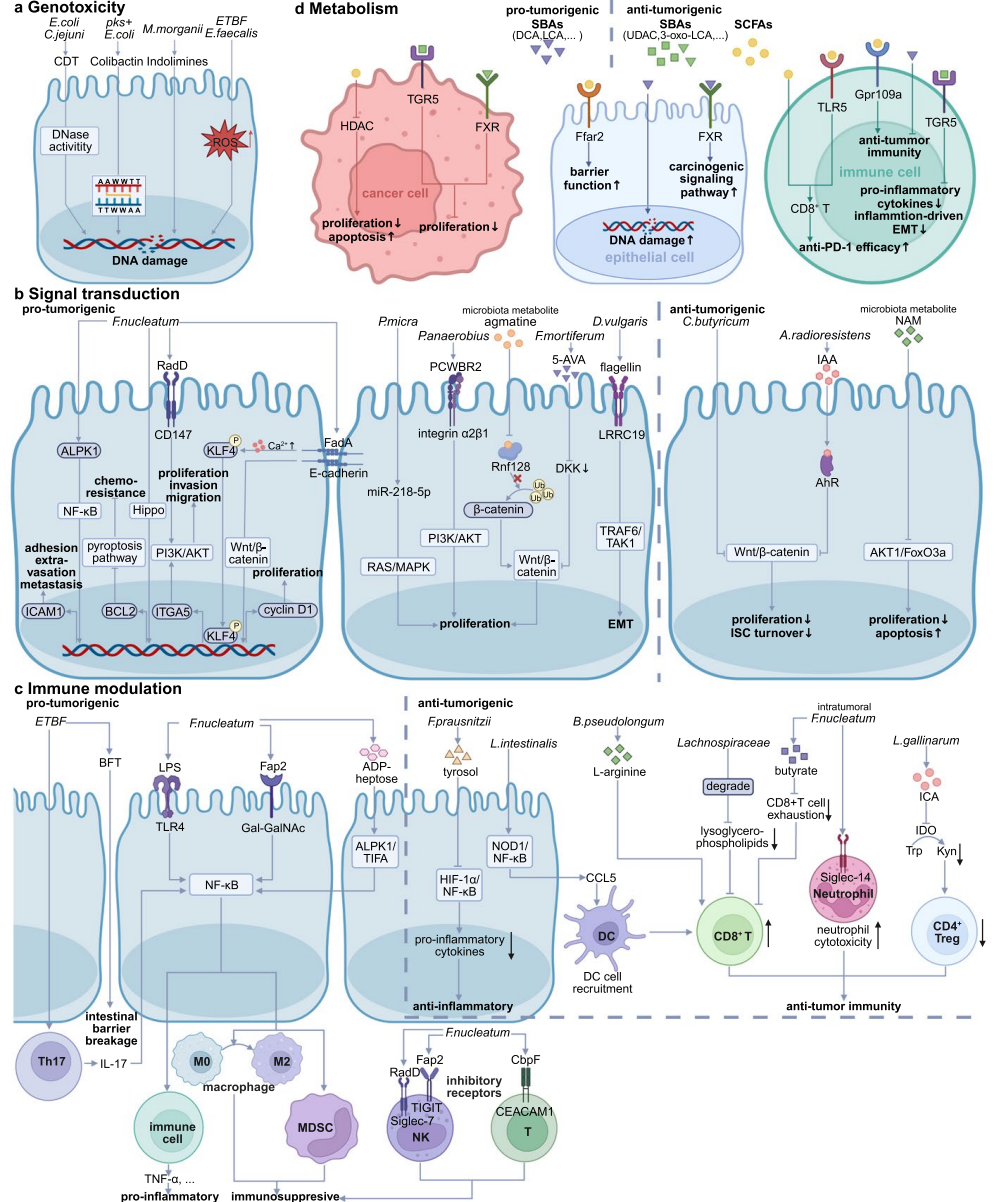
Mechanisms of gut microbiota in colorectal cancer. The mechanisms by which gut microbiota influence the initiation and progression of colorectal cancer primarily include the following four mechanisms. **a** Genotoxicity. Gut microbiota can produce genotoxic substances primarily including cytolethal distending toxin (CDT), colibactin, and indoleamine, that induce mutations or genomic instability, directly contributing to CRC initiation. In addition, some pathogenic bacteria can indirectly induce DNA damage by triggering host reactive oxygen species (ROS) production. **b** Signal transduction. Gut microbiota influences CRC development by modulating oncogenic pathways such as Wnt/β-catenin, NF-κB, and PI3K/AKT, as pathogenic bacteria often promote tumorigenesis through activation or enhancement of downstream signaling, while some microbes also exerting protective effects. **c** Immune modulation. The microbiota shapes the tumor microenvironment through immune modulation with dual effects. It can promote the development and progression of CRC by promoting inflammation and mediating immune suppression, while also contributing to beneficial effects by activating anti-tumor immunity. **d** Metabolism. Microbial metabolites are also associated with tumor initiation and progression. The most widely discussed metabolites linked to CRC include short-chain fatty acids (SCFAs) which exert protective effects and secondary bile acids (SBAs) that most of them promote tumorigenesis with ursodeoxycholic acid (UDCA) as an exception

CRC development involves complex genetic and environmental interactions. Although the causal relationship between gut microbiota dysbiosis and colorectal cancer remains incompletely defined, microbiota from CRC patients can promote intestinal carcinogenesis in mice [132, 133], supporting the involvement of microbes in CRC initiation and progression. The mechanisms can be broadly categorized into genotoxicity, signaling modulation, immune regulation, and metabolic pathways (Fig. 4).

##### Genotoxicity

Microbial genotoxicity provides a direct mechanistic link between CRC-associated dysbiosis and epithelial genomic instability (Fig. 4a). Gut microbes can produce genotoxic substances, primarily including cytolethal distending toxin (CDT), colibactin, and indoleamine, which damage colorectal epithelial DNA and may contribute to driver mutation accumulation, including alterations in *APC* (*adenomatous polyposis coli*), thereby facilitating the adenoma–carcinoma sequence.

CDT, produced by pathogenic *Escherichia coli* and *Campylobacter jejuni*, induces DNA double-strand breaks via deoxyribonuclease activity [134]. The reduced carcinogenic and metastatic potential of cdtB-mutant strains underscores the role of CDT in tumorigenesis [135, 136]. Colibactin, produced by *pks⁺ Escherichia coli* (*pks⁺ E. coli*) [137], preferentially targets AAWWTT DNA motifs [138], forming adducts and crosslinks that cause double-strand breaks [139, 140]. Its pro-tumorigenic effects have been demonstrated in experimental models [141, 142], and it has also been linked to chemotherapy and immunotherapy resistance [143, 144]. Consistently, colibactin-related mutational signatures have been identified in human CRC genomes [145, 146], while inhibition of its biosynthesis or blockade of adhesin-mediated epithelial binding can attenuate its genotoxic and tumor-promoting effects [147, 148]. In addition, *Morganella morganii* produces indolimines, which contain functional imine groups that can also mediate DNA damage and promote CRC development [149]. Some pathogens may further induce genomic instability indirectly by triggering host reactive oxygen species (ROS) production. For example, *Bacteroides fragilis* toxin (BFT) from enterotoxigenic* Bacteroides fragilis* (ETBF) induces ROS accumulation and DNA damage in colonic cells [150, 151], and *Enterococcus faecalis* exerts similar effects [152]. Collectively, these findings suggest that microbial genotoxicity contributes to CRC by inducing direct DNA damage and ROS-associated genomic instability, thereby promoting mutational accumulation during tumor development.

##### Signal transduction

Colorectal cancer commonly involves oncogenic pathways such as Wnt/β-catenin, NF-κB (nuclear factor kappa B), and PI3K (phosphoinositide 3-kinase)/AKT, with additional pathways identified in recent years (Fig. 4b). CRC-associated microbes can influence tumor development by converging on these host signaling networks, thereby promoting epithelial proliferation, resistance to apoptosis, invasion, metastasis, and therapy resistance. *Fusobacterium nucleatum* (*F. nucleatum*) is one of the best-studied examples and acts through several virulence factors. Its adhesin FadA (*Fusobacterium* adhesin A) binds E-cadherin to activate Wnt/β-catenin signaling and upregulate downstream proliferative genes such as cyclin D1 [153, 154]. FadA also enhances E-cadherin–KLF4 (Krüppel-like factor 4) signaling in a Ca^2^⁺-dependent manner, resulting in KLF4 phosphorylation and nuclear translocation, which drives integrin α5 transcription and metastatic progression [155]. Another virulence factor, RadD (radiation resistance protein D), facilitates *F. nucleatum* enrichment and colonization in tumor tissues through CD147 binding and activates PI3K/AKT signaling [156]. Beyond these effects, *F. nucleatum* can also promote chemoresistance by modulating the YAP/BCL2/Caspase-3/GSDME (Yes-associated protein/B-cell lymphoma-2/Caspase-3/gasdermin E) pathway [157] and promote metastatic dissemination through the ALPK1/NF-κB/ICAM1 (alpha-kinase 1/nuclear factor kappa B/intercellular adhesion molecule 1) axis [158].

Other CRC-associated bacteria also modulate host signaling pathways involved in tumor progression. *Parvimonas micra* activates the miR-218-5p/RAS (rat sarcoma viral oncogene)/ERK (extracellular signal-regulated kinase)/c-Fos axis [159], whereas *Peptostreptococcus anaerobius* stimulates PI3K/AKT signaling through integrin α2β1 binding mediated by its PCWBR2 (putative cell wall binding repeat 2) protein [160]. In addition, *Desulfovibrio vulgaris* promotes epithelial-mesenchymal transition (EMT) through flagellin-mediated interaction with LRRC19 (leucine-rich repeat containing 19) and activation of the TRAF6/TAK1 (tumor necrosis factor receptor-associated factor 6/transforming growth factor-β-activated kinase 1) signaling pathway [161]. Beyond direct bacterial signaling, microbial metabolites can also reshape oncogenic programs. Agmatine enhances Wnt/β-catenin signaling by inhibiting Rnf128-mediated β-catenin ubiquitination, thereby upregulating targets such as Cyclin D1, Lgr5, CD44, and c-Myc [162], while 5-aminovaleric acid (5-AVA) derived from *Fusobacterium mortiferum* similarly activates this pathway through suppression of the tumor suppressor DKK2 [163]. These examples suggest that CRC-associated microbes do not act through a single signaling route. Instead, they may reshape tumor-promoting signaling through bacterial adhesion, virulence factor–host receptor interactions, and microbiota-derived metabolites.

Conversely, selected commensals and microbiota-derived metabolites can also restrain CRC progression by modulating signaling pathways. For example, *Clostridium butyricum* suppresses CRC progression by inhibiting the Wnt/β-catenin pathway and altering microbial composition [164]. Indole acetic acid (IAA) from *Acinetobacter radioresistens* activates AhR in intestinal stem cells to inhibit Wnt/β-catenin signaling, reducing cellular proliferation, intestinal stem cell (ISC) turnover, and tumorigenesis [165]. The peptidoglycan fragment N-acetylmuramic acid (NAM) binds AKT1, inhibits its phosphorylation, and suppresses the oncogenic AKT1–FoxO3a pathway, thereby impeding tumor progression [166]. Thus, microbiota-mediated signaling regulation in CRC is bidirectional rather than uniformly tumor-promoting. Dysbiotic microbes may activate oncogenic pathways, whereas selected commensals or metabolites may restrain tumor-promoting signaling and preserve epithelial homeostasis.

##### Immune modulation

The microbiota shapes the tumor microenvironment through immune modulation with dual effects, promoting CRC progression by driving inflammation and immune suppression while also supporting anti-tumor immunity (Fig. 4c).

Inflammation is a recognized risk factor for CRC, and dysbiosis is closely linked to intestinal inflammation [167]. Both microbiota from CRC patients [132, 133] and individual tumor-promoting pathogens [168, 169] can induce colonic inflammation and upregulate pro-inflammatory mediators in mice. A key mechanism is the activation of Th17 cells [169, 170]. For example, ETBF disrupts the intestinal barrier through BFT, which then rapidly induces Th17 activation and IL-17 secretion [169, 171]. IL-17 then acts on colonic epithelial cells to activate STAT3 (signal transducer and activator of transcription 3) and NF-κB signaling, thereby promoting inflammation and carcinogenesis [172, 173]. The gut microbiota can also modulate the tumor immune microenvironment through direct interactions with epithelial cells. *F. nucleatum*, for instance, activates the pattern recognition receptor TLR4 through LPS to trigger NF-κB-driven inflammation [174]. Its virulence factor Fap2 can also bind to galactose-N-acetyl-D-galactosamine (Gal-GalNAc), inducing tumor cells to secrete IL-8 and CXCL1, which further recruits immune cells to secrete pro-inflammatory cytokines [175, 176]. Moreover, the *F. nucleatum* metabolite ADP-heptose can also activate NF-κB through the ALPK1/TIFA/TRAF6 pathway, leading to increased IL-8 expression [177]. These processes reinforce tumor-associated inflammation.

Beyond promoting inflammation, CRC-associated bacteria can also foster an immunosuppressive tumor microenvironment. Th17-driven signaling can induce CXC chemokine production and recruit myeloid cells, including myeloid-derived suppressor cells (MDSCs), into the tumor microenvironment [178, 179]. MDSCs suppress effector T cells through mechanisms such as arginase production and induction of Treg cell differentiation, thus fostering immunosuppression [180, 181]. *F. nucleatum* further protects tumor cells from immune-mediated killing through Fap2- and CbpF-mediated binding to the inhibitory receptors TIGIT (T-cell immunoglobulin and immunoreceptor tyrosine-based inhibitory motif domain) [182] and CEACAM1 (carcinoembryonic antigen-related cell adhesion molecule 1) [183] on natural killer (NK) cells and T cells. Another adhesin, RadD, can bind Siglec-7 (sialic-acid-binding immunoglobulin-like lectin 7) on NK cells and exert immunosuppressive effects as well [184]. In addition, *F. nucleatum* promotes TLR4-dependent M2 macrophage polarization [185, 186] and selectively recruits immunosuppressive myeloid-derived cells, including MDSCs, tumor-associated neutrophils (TANs), tumor-associated macrophages (TAMs), and immature dendritic cells (DCs) [168], thereby further shaping a pro-tumor microenvironment [187]. These mechanisms allow CRC-associated microbes to weaken anti-tumor immune surveillance and promote immune evasion.

Conversely, the gut microbiota can also enhance anti-tumor immunity. A high abundance of Lachnospiraceae is associated with higher immune scores in advanced CRC [188] and preserves CD8+ T cell immune surveillance by degrading inhibitory lysoglycerophospholipids [189]. L-arginine from *Bifidobacterium pseudolongum* promotes memory CD8+ T cell formation and suppresses colorectal cancer progression [190]. *Lactobacillus gallinarum* enhances CD8 + T cell function and restrains CD4+ Treg differentiation by blocking the IDO1 (indoleamine 2,3-dioxygenase 1)/Kyn/AhR axis via indole-3-carboxylic acid (ICA) [191]. *Faecalibacterium prausnitzii* produces tyrosol to inhibit HIF-1α (hypoxia-inducible factor 1α)/NF-κB signaling, thereby reducing ROS and inflammatory cytokines and exerting anti-tumor effects [192]. In addition, *Lactobacillus intestinalis* induces tumor-derived CCL5 via NOD1/NF-κB signaling, thereby promoting dendritic cell recruitment and suppressing colorectal tumorigenesis [193]. Interestingly, even *F. nucleatum*, a bacterium more commonly linked to CRC progression, may under specific conditions contribute to anti-tumor immunity. Intratumoral *F. nucleatum* can enhance anti-PD-1 (programmed cell death 1) responses in microsatellite-stable CRC through butyrate-mediated relief of CD8+ T cell exhaustion [194], and may also promote neutrophil cytotoxicity by activating Siglec-14-dependent tumor cell killing in a host genotype-dependent manner [195]. These seemingly contradictory findings underscore that the effects of *F. nucleatum* in CRC are highly context-specific and may depend on its spatial and ecological niche within tumors, the tumor molecular and therapeutic setting, and host genetic regulation of immune responsiveness. More broadly, they suggest that microbiota-immune interactions in CRC are better understood as conditional and state-dependent rather than uniformly tumor-promoting or tumor-suppressive.

##### Metabolism

Gut microbiota can metabolize host-derived and dietary substrates into a wide range of bioactive compounds that influence CRC initiation and progression [196, 197] (Fig. 4d). Among these microbiota-derived metabolites, SCFAs and secondary bile acids (SBAs) are the most extensively studied classes.

SCFAs, such as acetate, butyrate, and propionate, are produced by bacterial fermentation of indigestible carbohydrates in the colon [198]. They are generally thought to protect against CRC through immune modulation, especially butyrate [199]. By engaging receptors such as FFAR2 (free fatty acid receptor 2, also called GPR43) and GPR109A on epithelial and immune cells, they reinforce barrier integrity and enhance anti-tumor immunity [164, 200, 201]. As HDAC inhibitors, SCFAs also regulate Tregs and CD8+ T cells while suppressing tumor proliferation and promoting apoptosis [66, 68, 202]. Consistently, butyrate-producing bacteria and fecal butyrate levels are reduced in CRC [203], and lower SCFA levels are associated with increased CRC risk [204]. Butyrate may also enhance anti-PD-1 efficacy [205] and potentiate the pro-ferroptotic effect of oxaliplatin [206]. However, butyrate can also exert context-dependent pro-tumorigenic effects. For example, enrichment of specific butyrate-producing bacteria such as *Porphyromonas* spp. may increase local butyrate exposure and induce epithelial senescence and a senescence-associated secretory phenotype, thereby creating a tumor-promoting inflammatory microenvironment [207]. This apparent paradox likely reflects differences in host genetic background, microbial source, and epithelial metabolic state, which together determine whether butyrate functions predominantly as a homeostatic metabolite or as a pro-tumorigenic signal [208, 209].

Most bile acids are reabsorbed at the end of the ileum, but about 5%–10% enter the colon for further microbial metabolism [71]. Through the sequential actions of bile salt hydrolase [210] and bai operon-encoded 7α-dehydroxylase [211], bile acids undergo dehydroxylation to form SBAs, such as deoxycholic acid and lithocholic acid [212]. SBAs can promote tumorigenesis [71, 213] by activating carcinogenic signaling pathways [214, 215], inducing DNA damage [216, 217], and inhibiting anti-tumor immunity [218, 219]. They also promote CRC-associated dysbiosis, which can be alleviated by antibiotic-mediated microbiota depletion [220, 221]. These effects are mediated in part by bile acid receptors, particularly FXR [222, 223]. Supporting this, high colonic SBA levels in individuals with high-fat diets have been associated with increased CRC risk [224], and fecal metagenomic analyses further support enrichment of bile acid metabolism-related microbial signatures in CRC [225]. In contrast, ursodeoxycholic acid (UDCA), produced by *Ruminococcus gnavus* [226], appears protective against CRC, mainly through activation of the bile acid receptor TGR5 [227, 228]. Recent evidence further shows that the microbial bile acid 3-oxo-LCA (3-oxo-lithocholic acid) activates FXR signaling to inhibit cancer stem cell proliferation and induce apoptosis, highlighting its therapeutic potential in intestinal tumorigenesis [229].

Additionally, other metabolites such as hydrogen sulfide [230, 231], tryptophan metabolites [232–234], and polyamines [235, 236] have also been implicated in CRC. These findings indicate that CRC-associated microbial metabolism extends beyond SCFAs and SBAs, pointing to a broader network that may influence tumor initiation, progression, and therapeutic response.

#### Other gastrointestinal diseases

##### Inflammatory Bowel Disease (IBD)

Inflammatory bowel disease (IBD), a heterogeneous group of inflammatory diseases including ulcerative colitis (UC) and Crohn’s disease (CD) [237], is characterized by gut microbiota dysbiosis. This dysbiosis is typically marked by depletion of protective SCFA-producing bacteria, such as *Faecalibacterium prausnitzii* and *Roseburia* spp., and enrichment of pathobionts, including Enterobacteriaceae, *Enterococcus* spp., and *Fusobacterium* spp. [238, 239]. Transfer of microbiota from patients with CD to germ-free mice can induce colitis, further supporting a contributory role of the gut microbiota in IBD [240].

Under physiological conditions, commensal microbes maintain epithelial homeostasis and barrier integrity by regulating mucus secretion, preserving tight junctions, and producing beneficial metabolites. In IBD, disruption of these functions impairs barrier defense and promotes disease progression. In particular, depletion of SCFA-producing commensals reduces butyrate availability, which can compromise epithelial energy supply and tight junction stability [241–243]. Meanwhile, expansion of pathobionts can directly damage the epithelial barrier. *Adherent-invasive Escherichia coli* (AIEC) can adhere to and invade intestinal epithelial cells through adhesin-mediated interactions, such as FimH binding to cell adhesion molecule 6 receptor, and persist intracellularly, thereby increasing intestinal permeability [244, 245], whereas *Enterococcus faecalis* impairs epithelial integrity through gelatinase-mediated cleavage of E-cadherin [246].

Dysbiosis also amplifies intestinal inflammation and sustains disease activity through immune dysregulation [247]. AIEC can persist within macrophages and induce pro-inflammatory cytokine production [245, 248], and *Ruminococcus gnavus* has been linked to TNFα induction [249]. *F. nucleatum* can further exacerbate epithelial injury and inflammatory responses by inducing acetyl-CoA accumulation and activating STAT3 signaling [250]. The Th17/Treg imbalance observed in IBD is another important immune feature. It is characterized by increased Th17 responses and reduced Treg-mediated regulation, contributes to exaggerated mucosal immune activation, and is closely linked to microbial alterations. Reduced abundance of *Clostridium* spp. and *Bacteroides fragilis* has been implicated in impaired Treg regulation [251, 252], while *segmented filamentous bacteria* promote Th17 differentiation and IL-17/IL-22 production [62]. Oral-derived pathobionts such as *Porphyromonas gingivalis* can reshape gut microbial composition and suppress linoleic acid metabolism, thereby promoting Th17 differentiation while constraining Treg differentiation [253]. Conversely, *Roseburia intestinalis* promotes dendritic cell-mediated Treg differentiation through TLR5 signaling, suggesting that its depletion may likewise contribute to immune imbalance and intestinal injury [254].

Notably, dysbiosis-associated metabolic disturbances, including alterations in SCFAs, bile acids, and tryptophan metabolites, also play important roles in shaping immune responses and promoting inflammation [125, 247]. For example, microbiota-derived indole-3-propionic acid (IPA) is reduced in patients with IBD and can limit mucosal inflammation by promoting apoptosis of Th1/Th17 cells [255]. Likewise, depletion of *Odoribacter splanchnicus* in colitis may impair microbial secondary bile acid transformation, thereby reducing the suppression of neutrophil extracellular trap formation and aggravating mucosal inflammation [256]. In addition, recent studies suggest that gut microbes can generate bioactive H₂S from sulfur-containing substrates via assimilatory sulfate reduction, linking disordered microbial sulfur metabolism to epithelial injury and chronic intestinal inflammation [257]. Together, barrier dysfunction and excessive immune activation drive the onset, persistence, and recurrence of chronic intestinal inflammation.

##### Irritable Bowel Syndrome (IBS)

Irritable bowel syndrome (IBS) is a common functional gastrointestinal disorder characterized by recurrent abdominal pain associated with altered bowel frequency and/or stool form in the absence of overt structural abnormalities [258, 259]. Accumulating evidence suggests that gut microbiota dysbiosis is involved in IBS pathophysiology, with IBS-associated dysbiosis generally characterized by reduced microbial diversity, depletion of protective commensals, and enrichment of symptom-associated taxa [260]. However, compared with other inflammatory or structural gastrointestinal diseases, IBS-related microbial alterations are more heterogeneous and lack disease-specific signatures, which may reflect differences across subtypes, including diarrhea-predominant IBS (IBS-D) and constipation-predominant IBS (IBS-C), as well as marked interindividual variability [260–262].

Mechanistically, beyond mild barrier dysfunction and mucosal immune activation, the more distinctive microbiota-related features of IBS involve effects on visceral sensation, intestinal motility, and gut–brain axis signaling [263, 264]. Dysbiosis can impair barrier integrity, allowing translocation of bacterial products or components into the subepithelial compartment and trigger low-grade mucosal immune activation, particularly involving mast cells [265]. These immune mediators can sensitize enteric neurons and contribute to visceral hypersensitivity [265, 266]. At the same time, microbial metabolites and immune signals may alter bidirectional gut–brain communication by modulating enteric neural activity, vagal signaling, and central processing of visceral stimuli, which may further amplify symptom perception in IBS [267, 268]. Such pathophysiological alterations may persist for years [269]. Microbial metabolic disturbances also shape symptom onset and exacerbation in IBS [270]. Disordered bile acid metabolism is an important mechanism, particularly in IBS-D [261, 271]. Impaired conversion of primary to secondary bile acids and bile acid overproduction, associated with increased *Escherichia coli* and Clostridia and reduced *Clostridium leptum* and *Bifidobacterium*, may lead to the accumulation of primary bile acids in the intestinal lumen, thereby stimulating fluid secretion and accelerating intestinal transit [270–272]. In IBS-C, increased abundance of methanogens such as *Methanobrevibacter smithii* has been observed [262, 273]. Methane delays ileal and colonic transit and reduces contractile amplitude, thereby slowing peristalsis and contributing to constipation [262, 274]. In addition, other microbiota-related alterations, including disturbed tryptophan metabolism and abnormal fermentation, may further shape IBS symptom patterns [270, 275]. Overall, given the heterogeneity and complexity of IBS, the effects of gut microbiota are difficult to generalize. The microbiota in IBS is more appropriately viewed as a modulator of disease phenotype and symptom severity than as a primary driver of disease initiation.

### Gut microbiota in extra-intestinal diseases: the gut–organ axis

#### Gut–liver axis

The gut and liver are intimately connected both anatomically and functionally, forming a bidirectional gut–liver axis [276–278]. From an anatomical perspective, intestinal blood drains into the liver through the portal venous system, which allows the liver to sense and process gut-derived nutrients, microbial metabolites, and bacterial components [276, 277, 279]. Functionally, enterohepatic bile acid circulation is central to this interaction. Primary bile acids synthesized in the liver are converted by the gut microbiota into secondary bile acids, which not only facilitate lipid digestion and absorption but also shape microbial composition and feed back to regulate intestinal and hepatic physiology [280, 281]. This tightly regulated crosstalk helps maintain metabolic homeostasis, immune balance, and barrier integrity, supporting the importance of gut microbial homeostasis for liver health. When gut microbiota dysbiosis develops, this communication can shift from a physiological to a pathological state.

##### Metabolic Dysfunction-Associated Steatotic Liver Disease (MASLD)

In metabolic dysfunction-associated steatotic liver disease (MASLD, also termed nonalcoholic fatty liver disease, NAFLD), the contribution of the gut microbiota is particularly evident in metabolic dysregulation and chronic inflammatory activation [282–284]. At the metabolic level, dysbiosis can disrupt bile acid metabolism by increasing hydrophobic toxic bile acids while suppressing intestinal FXR signaling and fibroblast growth factor 19 (FGF19) expression, thereby relieving the negative feedback inhibition of hepatic cholesterol 7α-hydroxylase (CYP7A1) and promoting bile acid pool expansion and hepatocellular lipotoxicity [285, 286]. Within this context, a recent study further showed that the microbial bile acid 3-sucCA (3-succinylated cholic acid), which mitigates metabolic dysfunction-associated steatohepatitis (MASH) by enriching *Akkermansia muciniphila*, is reduced in MASLD patients [36]. At the same time, depletion of SCFA-producing bacteria weakens the protective effects of short-chain fatty acids. However, when SCFAs exceed the host metabolic threshold, they may indirectly promote hepatic lipid deposition by serving as substrates for lipogenesis and activating GPR41/43 [287, 288]. Dysbiosis also alters other microbial metabolic pathways relevant to hepatic lipid accumulation and injury. LPS and pro-inflammatory cytokines can activate IDO, shifting tryptophan metabolism from the beneficial indole pathway toward the kynurenine pathway [289, 290]. In addition, anaerobic bacteria can produce substantial amounts of endogenous ethanol, directly inducing oxidative damage in hepatocytes [291, 292]. Trimethylamine (TMA)-producing bacteria can also metabolize choline into TMA, which is subsequently converted in the liver to TMAO, thereby reducing choline availability and promoting hepatic lipid accumulation [293, 294].

At the inflammatory level, barrier dysfunction further increases hepatic exposure to LPS and other pathogen-associated molecular patterns (PAMPs), thereby enhancing TLR-mediated inflammatory signaling, including NLRP3 inflammasome assembly and innate immune activation [295, 296]. Gut-derived high-density lipoprotein subspecies HDL3 normally restrains LPS-driven hepatic inflammation by preventing the interaction between LPS and LPS-binding protein (LBP), whereas this protective effect is weakened during MASLD progression [297]. Collectively, gut microbiota dysbiosis may contribute to MASLD progression from simple steatosis to steatohepatitis and fibrosis through metabolic dysregulation and chronic inflammatory activation.

##### Primary Sclerosing Cholangitis (PSC)

In primary sclerosing cholangitis (PSC), gut microbiota dysbiosis is closely linked to aberrant gut–liver immune crosstalk [298, 299]. The close association between PSC and IBD suggests that persistent activation of mucosal immunity can contribute to biliary injury through the gut–liver axis [298]. Following mucosal immune activation, dendritic cells process antigens and imprint gut-homing properties on T cells, including α4β7 integrin and CCR9 [300, 301]. In PSC, the liver aberrantly expresses the corresponding ligands, MAdCAM-1 and CCL25, thereby promoting the recruitment of gut-primed lymphocytes and contributing to peribiliary inflammation and tissue injury [300, 301]. This pathogenic process may be reinforced by PSC-associated dysbiosis, which is characterized by reduced microbial diversity and enrichment of potentially pro-inflammatory taxa such as *Veillonella*, *Enterococcus*, and *Streptococcus* [298, 299]. Such dysbiosis may sustain intestinal immune activation and enhance gut-derived inflammatory signaling [298, 299]. Upon exposure to gut-derived PAMPs, cholangiocytes respond through pattern recognition receptors and produce chemokines and cytokines, which further recruit and activate local immune cells and promote inflammation and periductal fibrosis [302, 303]. Moreover, shared T-cell clonotypes have been identified in the liver and intestine of patients with PSC, supporting the idea that antigen-driven adaptive immune responses may operate across both organs [298, 304]. Overall, these findings suggest that, in PSC, gut microbiota dysbiosis may promote biliary inflammation, fibrosis, and liver injury by sustaining abnormal immune communication between the intestine and liver.

##### Cirrhosis

Cirrhosis can be viewed as a more advanced stage of gut–liver axis dysfunction, in which dysbiosis is no longer confined to a local intestinal abnormality but is closely linked to decompensation and multiple complications [276, 305]. Typical features include depletion of commensals such as *Lachnospiraceae* and *Ruminococcaceae*, together with enrichment of potentially pathogenic taxa, including *Enterobacteriaceae*, *Enterococcaceae*, and *Streptococcaceae* [306, 307]. This dysbiotic state can weaken colonization resistance, promote small intestinal bacterial overgrowth, facilitate recurrent translocation of bacteria and their products, and maintain systemic exposure to endotoxins and other gut-derived harmful molecules [308]. At the same time, portal hypertension, intestinal edema, delayed transit, reduced bile flow, and impaired hepatic clearance further aggravate this imbalance, creating a self-reinforcing cycle between gut ecological disruption and worsening liver function [277, 309]. This cycle may drive progression from compensated to decompensated cirrhosis. In addition, cirrhosis-associated dysbiosis may contribute to the development of related complications. For example, it can disturb intestinal nitrogen and ammonia metabolism, increase the burden of hyperammonemia, and promote hepatic encephalopathy [310, 311].

#### Gut–brain axis

The intestinal microbiota and the central nervous system maintain continuous and dynamic bidirectional communication through the microbiota–gut–brain axis (MGBA), which is mediated by microbial metabolites, immune signaling, hormone-like signals released by enteroendocrine cells (EECs), and peripheral nerves, particularly vagal afferent pathways [90, 312]. Microbial metabolites can influence central nervous system function by acting on the blood–brain barrier and various target cells within the brain [80, 312]. Furthermore, the regulatory effects of the gut microbiota on local and peripheral immune systems can reshape the central neuroimmune environment through cytokines, chemokines, and other immune mediators [313]. Concurrently, EECs can sense microbial and metabolic signals and release diverse hormone-like molecules that modulate neural activity and behavioral responses [314]. Peripheral sensory nerves innervating the gut provide a rapid pathway for transmitting intraluminal signals to the central nervous system. Conversely, the central nervous system can regulate gut motility, secretion, barrier integrity, mucosal immunity, and microbial ecology via the hypothalamic–pituitary–adrenal (HPA) axis and peripheral efferent nerves [90]. When gut microbial homeostasis is disrupted, this finely tuned bidirectional physiological communication may progressively shift toward pathological imbalance, thereby contributing to neurological dysfunction and disease susceptibility.

##### Autism Spectrum Disorder (ASD)

In neurodevelopmental disorders, particularly autism spectrum disorder (ASD), the MGBA is closely involved in early-life immune programming [315]. Individuals with ASD commonly show reduced gut microbial diversity and lower levels of beneficial bacteria, which correlate with gastrointestinal symptoms and the severity of behavioral symptoms [315, 316]. Recent work has shown that ASD model mice (BTBR strain) display abnormal accumulation of brain-resident CD4+ T cells [317]. These cells can promote neuroinflammation through the production of pro-inflammatory cytokines [317]. Depletion of these T cells or maintenance of mice under germ-free conditions can ameliorate ASD-related behaviors, supporting a causal link along a gut microbiota–brain immune cell–behavior axis [317]. Metabolically, specific microbial metabolites, such as 4-ethylphenyl sulfate (4-EPS) and p-cresol sulfate (pCS), have been implicated in abnormal neurodevelopmental processes, including altered myelination and synaptic function [318]. Additionally, individuals with ASD often present with an imbalance in the glutamate/GABA ratio, reflecting an excitation–inhibition imbalance that is considered relevant to core ASD symptoms [319]. The gut microbiota may contribute to the maintenance of this balance by producing or modulating neurotransmitter precursors [320]. These MGBA-related mechanisms may be especially important during the perinatal period and early infancy, when patterns of microbial colonization may exert long-lasting programming effects on social behavior and stress responsiveness by shaping microglial maturation and the neuroimmune microenvironment [321].

##### Parkinson’s Disease (PD)

In neurodegenerative disorders, Parkinson’s disease (PD) provides a distinctive framework for understanding the role of the MGBA in the propagation of protein pathology [322, 323]. The pathological hallmarks of PD include the progressive loss of dopaminergic neurons in the substantia nigra pars compacta and the abnormal aggregation of α-synuclein into Lewy bodies [322, 323]. Gastrointestinal dysfunction, particularly constipation, often precedes motor symptoms by years or even decades [324], which is consistent with the Braak hypothesis that PD may, at least in part, originate in the gut [325, 326]. Unlike Alzheimer’s disease, where MGBA-related mechanisms are often discussed in the context of chronic inflammation-associated protein aggregation, the MGBA in PD is more specifically centered on the initiation of α-synuclein pathology in the gut and its subsequent retrograde spread along the gut–brain axis [316]. EECs, which are chemosensory cells within the intestinal epithelium, endogenously express α-synuclein and form synapse-like connections with enteric neurons and vagal afferent terminals, forming a potential anatomical interface between the intestinal environment and the central nervous system [314]. Gut microbial dysbiosis can increase exposure to bacterial components and inflammatory stimuli, which may promote local innate immune activation and oxidative stress, and create a permissive microenvironment for α-synuclein misfolding and aggregation [327, 328]. These aberrantly folded proteins may then spread retrogradely in a prion-like manner via the vagus nerve to the dorsal motor nucleus and subsequently to other brain regions, including the substantia nigra [322, 323, 329]. Under conditions of impaired intestinal and blood–brain barrier integrity, bacterial products and microbial metabolites may also activate microglia and astrocytes and interfere with α-synuclein clearance, further amplifying neuroinflammatory responses [330]. Overall, current evidence supports a model in which gut dysbiosis may promote early intestinal α-synuclein pathology, followed by gut-to-brain propagation and chronic neuroinflammation, and ultimately to progressive dopaminergic neurodegeneration. This framework helps explain why gastrointestinal symptoms often precede motor manifestations and provides a rationale for further exploration of microbiota-targeted strategies in PD.

##### Major Depressive Disorder (MDD)

Major depressive disorder (MDD) is a prototypical affective disorder in which microbiota dysbiosis has been linked to chronic low-grade inflammation, altered tryptophan metabolism, and dysregulation of the HPA axis [331, 332]. Dysbiosis can impair intestinal barrier homeostasis, promoting LPS translocation into the circulation and systemic pro-inflammatory signaling, thereby sustaining peripheral low-grade inflammation [80, 331]. This inflammatory state not only disrupts blood–brain barrier integrity and the central immune milieu, but also enhances IDO activity, diverting tryptophan metabolism from the relatively mood-supportive 5-hydroxytryptamine (5-HT) pathway toward the kynurenine pathway [333, 334]. As a result, the availability of neurotransmitter precursors is reduced, while potentially neurotoxic intermediates accumulate [335, 336]. In parallel, gut dysbiosis can further disrupt emotional regulation by altering the production, bioavailability, and signaling of GABA, dopamine, norepinephrine, and their precursors [320, 332]. In addition, the gut microbiota is increasingly recognized as an important regulator of HPA axis programming and stress reactivity [337]. Under inflammatory conditions, persistent HPA axis dysregulation may result in chronically elevated and poorly controlled cortisol levels [338]. Importantly, MGBA dysfunction in MDD is characterized by a bidirectional amplification loop in which chronic stress and heightened HPA axis reactivity further increase intestinal permeability, alter gut motility, and disrupt microbial composition, whereas persistent dysbiosis, in turn, exacerbates inflammatory burden and stress sensitivity [332, 339]. Together, these processes link immune activation, metabolic disturbance, and neuroendocrine dysregulation in a self-reinforcing cycle that may contribute to MDD pathophysiology.

#### Gut–endocrine axis

The gut–endocrine axis is a bidirectional communication network linking the gut microbiota, enteroendocrine cells, neural circuits, and peripheral endocrine organs. Within this axis, microbiota-derived metabolites, particularly SCFAs, bile acid derivatives, and tryptophan metabolites, act as important signaling mediators that regulate enteroendocrine hormone secretion and thereby influence systemic endocrine homeostasis [127, 340]. Enteroendocrine cells, such as L and K cells, sense luminal nutritional and microbial cues and secrete hormones including GLP-1, PYY, and GIP, linking the intestinal microbial environment to appetite regulation, insulin secretion, glucose metabolism, and energy homeostasis [341]. Gut-derived hormonal and metabolic signals can also be sensed through vagal and neuroendocrine pathways, extending the regulatory influence of the gut microbiota on host endocrine function through the hypothalamic–pituitary–adrenal (HPA), hypothalamic–pituitary–thyroid (HPT), and hypothalamic–pituitary–gonadal (HPG) axes [342].

##### Type 2 Diabetes Mellitus (T2DM)

In type 2 diabetes mellitus (T2DM), dysfunction of the gut–endocrine axis involves not only dysbiosis-induced impairment of intestinal barrier integrity and chronic inflammation, but more importantly a reduced capacity to convert luminal microbial and nutritional cues into endocrine regulatory signals [343]. T2DM-associated dysbiosis is often accompanied by depletion of butyrate-producing bacteria and reduced SCFA biosynthesis [344], which may weaken SCFA-mediated activation of GPR41/43 on L cells and thereby reduce GLP-1 secretion [86]. Concurrently, dysbiosis-related disturbances in bile acid metabolism may disrupt TGR5-dependent incretin release, further blunting postprandial gut hormone responses [345]. In contrast, certain microbial metabolites, such as high concentrations of acetate, may stimulate ghrelin release, thereby promoting appetite [346]. These alterations attenuate satiety signaling, impair gastric emptying control, accelerate postprandial glucose entry into the circulation, and weaken hormonal regulation of nutrient handling and postprandial energy metabolism [347]. As the major incretin hormones linking intestinal and pancreatic endocrine function, GLP-1 and GIP are essential for glucose-dependent insulin secretion [347]. Thus, reduced secretion or impaired action of these hormones compromises β-cell compensation in response to postprandial glucose stimulation [348]. Gut microbial metabolites may also directly influence glucose homeostasis. Because GPR41/43 receptors are expressed on β-cells, SCFAs may directly influence β-cell function [349]. Elevated branched-chain amino acid (BCAA) levels have been associated with insulin resistance and increased T2DM risk [350], while altered microbial carbohydrate metabolism, characterized by increased host-accessible monosaccharides in individuals with insulin resistance, may further contribute to metabolic dysfunction [351]. Moreover, dysbiosis may disturb bile acid-related signaling, thereby reducing adipose thermogenesis and white adipose tissue browning, while altering hepatic fatty acid oxidation and metabolic reprogramming [352]. These changes may reduce host energy expenditure, promote lipid accumulation, aggravate insulin resistance, and further contribute to the development and progression of T2DM [352].

#### Other gut–organ axes

Beyond the gut–liver, gut–brain, and gut–endocrine axes, gut microbiota dysbiosis has also been implicated in a broad range of extra-intestinal diseases through interconnected mechanisms [74]. Microbial-derived metabolites, including SCFAs, bile acid derivatives, tryptophan metabolites, trimethylamine N-oxide, and uremic toxins, can enter the portal or systemic circulation and influence metabolic homeostasis, inflammatory responses, and cellular function in distant organs [353]. These effects are especially relevant to the gut–heart and gut–kidney axes. TMAO has been linked to vascular inflammation, atherosclerosis, hypertension, and heart failure [354, 355], whereas uremic toxins such as indoxyl sulfate and p-cresyl sulfate may aggravate chronic kidney disease and reinforce the vicious cycle between intestinal and renal dysfunction [356, 357].

Dysbiosis may also weaken intestinal barrier integrity and facilitate the translocation of bacteria and microbial components such as lipopolysaccharide into the circulation, which can amplify systemic inflammation and worsen distant organ injury [38]. In the gut–kidney axis, barrier disruption may accelerate systemic toxin exposure and promote renal damage [356, 358], while in the gut–heart axis, it may contribute to endothelial dysfunction and chronic vascular inflammation [359]. Within the gut–lung axis, translocated microbial products may act as gut-derived inflammatory signals, sustaining pulmonary immune dysregulation and disease progression [360, 361].

These alterations are frequently accompanied by immune dysregulation and persistent low-grade inflammation, which further translate gut-derived perturbations into organ-specific pathology [58]. This is especially apparent in the gut–lung axis, where dysbiosis and reduced protective metabolites may impair immune tolerance and reshape pulmonary innate and adaptive immunity [362, 363]. Such changes can promote airway inflammation and increase susceptibility to asthma, chronic obstructive pulmonary disease, and respiratory infections [362, 363]. Comparable immune remodeling has also been implicated in the gut–skin [364] and gut–bone axes [365], contributing to psoriasis and atopic dermatitis, as well as osteoporosis and bone loss.

Gut–organ communication is further modulated by neuroendocrine–metabolic reprogramming, including changes in autonomic and vagal signaling, gut hormone secretion, nutrient sensing, and host metabolic pathways [366]. Beyond the gut–brain and gut–endocrine axes, such mechanisms may also operate along the gut–reproductive axis, where gut microbiota dysbiosis has been linked to hormonal imbalance, ovarian dysfunction, and reproductive metabolic disturbances, thereby contributing to disorders such as polycystic ovary syndrome and gynecological cancer [367, 368]. Taken together, these examples point to an interconnected gut–organ network through which dysbiosis may affect extra-intestinal organs via metabolic, inflammatory, immune, and neuroendocrine signals.

## Clinical applications and translational perspectives of the gut microbiota in disease

**Fig. 5:**
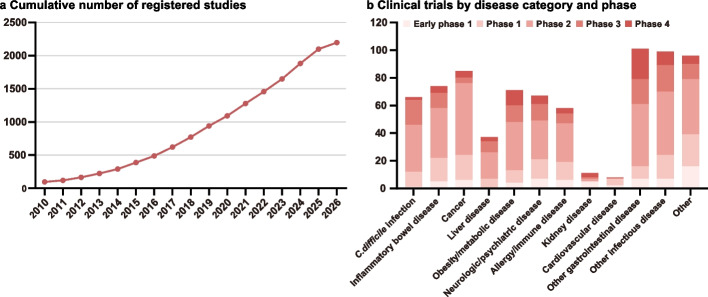
Clinical trials of gut microbiota-related research. This figure summarizes the overall landscape of gut microbiota-related clinical trials registered in ClinicalTrials.gov. Data were updated through April 2026. **a** Cumulative number of registered studies. Cumulative number of registered studies over time. **b** Clinical trials by disease category and phase. Number of registered studies across disease categories and clinical phases.

Building on mechanistic evidence linking the gut microbiota to disease, recent research has increasingly shifted toward clinical translation, with particular emphasis on biomarker development, microbiota-targeted interventions, and their potential in precision medicine. Consistent with this trend, microbiota-related clinical trials have increased substantially in recent years, reflecting growing translational interest across multiple disease areas (Fig. 5).

### Microbiota-based biomarkers

**Fig. 6:**
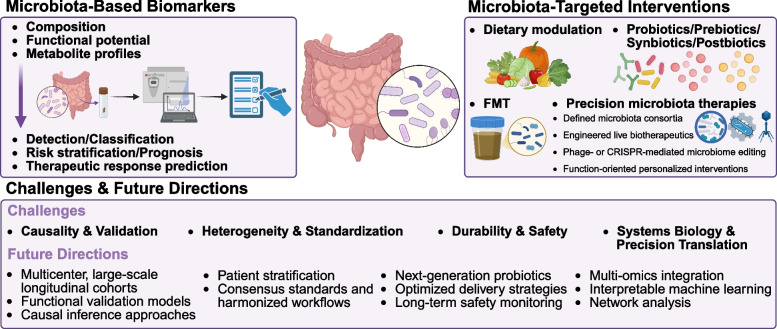
Translational potential of the gut microbiota in disease. Given its important role in a wide range of diseases, the gut microbiota has broad translational potential in the clinic, particularly as a source of biomarkers for disease detection, risk stratification, and response prediction, and as a therapeutic target for dietary modulation, biotic products, fecal microbiota transplantation, and precision microbiota therapies. At the same time, its further clinical application remains limited by challenges in causal validation, heterogeneity and standardization, durability and safety of interventions, and systems complexity, thereby driving future development toward more rigorous validation, improved harmonization and stratification, safer and more durable interventions, and increasingly integrative, precision microbiota medicine

Microbiota-based biomarkers represent a major area of microbiome clinical translation [11, 369] (Fig. 6). Compared with conventional single-molecule biomarkers, microbiota-based biomarkers can capture disease-associated alterations at multiple levels, including microbial composition, functional potential, and metabolite profiles, and therefore provide richer biological information [370]. Many of these signatures are also accessible through noninvasive fecal samples, which makes them attractive for disease screening, adjunctive diagnosis, patient stratification, and longitudinal monitoring [11, 369]. Their clinical value, however, does not lie simply in describing increases or decreases in specific microbial taxa, but in whether such signatures reliably reflect disease-relevant biological processes and complement existing clinical assessment tools [12, 371].

The clinical utility of gut microbiota-based biomarkers is currently primarily evident in disease detection and classification, risk stratification and prognostic assessment, and prediction of therapeutic response. For disease detection and classification, gut microbial signatures have shown potential as noninvasive tools, particularly in disorders such as IBD and CRC. Patients with IBD exhibit marked alterations in microbial composition and function compared with healthy individuals, while UC and CD may also display partially distinct microbial features [371–373]. For example, IBD is often characterized by depletion of beneficial commensals and SCFA-related metabolic disturbances, with some microbial alterations showing subtype preference, such as a stronger association of *Escherichia coli* with CD in certain cohorts [371, 372]. These findings support the use of microbiota-related signatures as adjunctive tools alongside endoscopy, imaging, and conventional inflammatory markers. In CRC, carcinogenesis-associated microbes and metabolic disturbances also show potential as biomarkers for early screening and risk identification, and may improve detection efficiency when integrated with established screening approaches [225, 374, 375].

Beyond diagnosis, microbiota-based biomarkers may reflect disease activity and prognostic risk. Patterns of dysbiosis, altered functional pathways, or disturbed metabolite profiles may correlate with disease activity, relapse tendency, complication risk, disease progression, or stage-specific differences, thereby supporting patient stratification and management [376–378]. For example, microbiome alterations have been implicated in the predisease and progressive phases of IBD [376, 377]. Reproducible microbial signatures have also been linked to CRC progression and stage-specific stratification, with *Parvimonas micra* and *Fusobacterium nucleatum* increasing from stage I onward, whereas *Akkermansia muciniphila* and *Parabacteroides distasonis* appear to be more enriched in late-stage CRC [378].

Another important application is prediction of therapeutic response. Baseline microbial states and metabolic capacity may partly explain heterogeneous responses to dietary interventions, metabolic therapies, and cancer immunotherapy [379–382]. For instance, gut microbiome features may help predict personalized responses to dietary fiber intervention in prediabetes [379] and are also being explored as biomarkers of response to immune checkpoint inhibitors across multiple cancers [380–382]. Overall, microbiota-based biomarkers may provide complementary biological information to refine clinical interpretation and individualized disease management.

### Microbiota-targeted interventions

Beyond biomarker applications, the gut microbiota has emerged as a promising therapeutic target (Fig. 6). Current microbiota-targeted interventions aim to restore microbial balance, recover beneficial microbial functions, and re-establish host–microbiota homeostasis. Representative completed clinical trials across different disease contexts and intervention strategies are summarized in Table 1, highlighting the translational potential of microbiota-based therapies.

**Table 1:** Representative completed clinical trials of microbiota-targeted interventions

Study (NCT)	Disease	Microbiota intervention	Study design	Phase	Key clinical outcome	Main findings	Translational significance	Ref
Fresh vs frozen FMT for recurrent CDI (NCT01398969)	Recurrent *Clostridioides difficile* infection	Fresh FMT vs frozen-and-thawed FMT	Randomized, double-blind, noninferiority trial (*n* = 232)	PHASE2	Clinical resolution of diarrhea without relapse at 13 weeks and adverse events	Frozen FMT was non-inferior to fresh FMT for clinical resolution of CDI, with no significant differences in adverse events	Supports the transition from fresh to standardized FMT, advancing the clinical translation of microbiota-based therapies in rCDI	[383]
RBX2660 for recurrent CDI (PUNCHCD3) (NCT03244644)	Recurrent *Clostridioides difficile* infection	Microbiota-based therapy (RBX2660) vs placebo	Randomized, double-blind, placebo-controlled trial (*n* = 289)	PHASE3	Absence of CDI diarrhea within 8 weeks	RBX2660 improved treatment success compared with placebo and showed sustained response through 6 months, with an acceptable safety profile	Represents an FDA-approved standardized microbiota-based therapeutic for preventing rCDI recurrence, highlighting the clinical maturation of microbiota-based therapy	[384, 385]
SER-109 vs placebo for recurrent CDI (ECOSPOR III) (NCT03183128)	Recurrent *Clostridioides difficile* infection	Microbiota-based therapy (SER-109) vs placebo	Randomized, double-blind, placebo-controlled trial (*n* = 182)	PHASE3	Recurrence of CDI up to 8 weeks	SER-109 significantly reduced CDI recurrence compared with placebo, with an acceptable safety profile	Demonstrates the efficacy of an FDA-approved oral microbiota-based therapeutic for preventing rCDI recurrence, further advancing standardized microbiota therapy beyond conventional delivery approaches	[386]
VE303 for prevention of recurrent CDI (NCT03788434)	Recurrent *Clostridioides difficile* infection	Defined bacterial consortium (VE303) vs placebo	Randomized, double-blind, placebo-controlled, dose-ranging trial (*n* = 79)	PHASE2	Recurrence of CDI at 8 weeks using a combined clinical and laboratory definition	High-dose VE303 reduced CDI recurrence compared with placebo, while a further phase 3 validation is needed	Supports defined bacterial consortia as a standardized next-generation microbiota therapy for preventing CDI recurrence	[387]
FMT for ulcerative colitis (NCT01896635)	Ulcerative colitis	FMT vs placebo	Multicentre, randomized, double-blind, placebo-controlled trial (*n* = 85)	PHASE2	Steroid-free clinical remission with endoscopic remission or response (Mayo score-based) at week 8	FMT increased clinical remission and endoscopic response compared with placebo in active ulcerative colitis	Supports FMT as a potential microbiota-based therapy for UC	[388, 389]
Fecal biotherapy for induction of remission in ulcerative colitis (NCT01545908)	Ulcerative colitis	FMT vs placebo	Randomized, double-blind, placebo-controlled trial (*n* = 75)	PHASE2	remission of UC (Mayo score-based) at week 7	FMT induced remission in a higher proportion of patients with active ulcerative colitis than placebo, with similar adverse event rates	Supports FMT as an investigational microbiota-based therapy for UC, while highlighting the potential importance of donor- and disease-duration-related effects	[390]
FMT plus dietary fiber in ulcerative colitis (NCT03998488)	Ulcerative colitis	FMT vs placebo, with or without psyllium fiber	Randomized, triple-blind, crossover trial (*n* = 27)	PHASE2	Clinical response (Mayo score-based) at week 8	FMT improved clinical response, remission, and endoscopic outcomes compared with placebo, while psyllium fiber did not further improve clinical outcomes	Evaluates the transition from FMT alone to combined microbiota–dietary modulation strategies in UC, while highlighting donor-dependent engraftment as a key determinant of response	[391]
FMT to enhance immune checkpoint therapy in renal cell carcinoma (NCT04758507)	Renal cell carcinoma	FMT vs placebo, with pembrolizumab and axitinib	Randomized, double-blind placebo-controlled trial (*n* = 50)	PHASE2	12-month progression-free survival (PFS), with median PFS and median overall survival, objective response rate (ORR), safety and microbiome changes as secondary outcomes	Donor FMT from complete responders to immunotherapy did not significantly improve the primary endpoint of 12-month PFS but did significantly improve median PFS versus placebo FMT	Highlights the potential of microbiota modulation to improve immune checkpoint inhibitor therapy in cancer	[392]
FMT plus pembrolizumab in melanoma (NCT03341143)	Melanoma	FMT with Pembrolizumab	Single-arm clinical trial (*n* = 16)	PHASE2	Objective response rate (ORR) up to 3 years	FMT with pembrolizumab induced clinical responses in a subset of anti-PD-1–refractory melanoma patients, accompanied by donor microbiota engraftment and tumor immune remodeling	Suggests a potential role for FMT in improving anti-PD-1 responsiveness in selected patients, while supporting larger trials to define predictive biomarkers and responsive microbial consortia	[393]
Probiotic for prevention of irinotecan-induced diarrhea in CRC (NCT01410955)	Colorectal cancer	Probiotics vs placebo	Multicentre, randomized, double-blind, placebo-controlled trial (*n* = 46)	PHASE3	Prevention of grade 3–4 diarrhea up to 2 years	Probiotics in patients with CRC treated with irinotecan-based chemotherapy were safe and could lead to a reduction in the incidence and severity of gastrointestinal toxicity, while further phase 3 with adequate sample size is needed	Provides early randomized evidence for microbiota-based strategies to mitigate chemotherapy-induced gastrointestinal toxicity in colorectal cancer	[394]
Synbiotic therapy for radiation-induced gut injury in rectal cancer (NCT03420443)	Colorectal cancer	Prebiotic/synbiotic supplementation vs no supplementation	Randomized, triple-blind, parallel-group trial (*n* = 30)	N/A	Radiation-induced gastrointestinal mucosal response (assessed by microbial diversity and inflammation) at 2 weeks	Synbiotic supplementation reduced radiation-induced mucosal inflammation and microbiota disruption, with effects primarily localized to intestinal tissue	Supports synbiotic intervention as a preventive strategy to mitigate radiotherapy-induced intestinal injury through microbiota modulation in rectal cancer	[395]
Probiotic supplementation for inflammatory modulation in CRC (NCT03782428)	Colorectal cancer	Probiotics vs placebo	Randomized, double-blind, placebo-controlled trial (*n* = 52)	N/A	Changes in circulating inflammatory cytokine levels at 6 months	Probiotics were safe and reduced pro-inflammatory cytokine levels in CRC patients after surgery	Suggests a potential role for probiotics as a microbiota-based adjunct to modulate persistent inflammation in colorectal cancer	[396]
*Akkermansia muciniphila* supplementation in metabolic syndrome (NCT02637115)	Overweight/obesity with insulin resistance	Live or pasteurized *Akkermansia muciniphila* vs placebo	Randomized, double-blind, placebo-controlled trial (*n* = 40)	N/A	safety, tolerability and metabolic parameters (insulin resistance, circulating lipids, visceral adiposity and body mass) at 3 months	*Akkermansia muciniphila* supplementation was safe and well tolerated. Pasteurized *A. muciniphila* improved insulin sensitivity and reduced selected metabolic parameters	Supports next-generation probiotics as a potential microbiota-based strategy for metabolic syndrome	[397]
FMT and fiber supplementation in obesity and metabolic syndrome (NCT03477916)	Obesity and metabolic syndrome	Oral lean-donor FMT vs placebo FMT, with cellulose or prebiotic fiber	Randomized, double-blind, placebo-controlled trial (*n* = 70)	PHASE2	Change in insulin sensitivity at 6 weeks	Oral FMT followed by low-fermentable fiber improved insulin sensitivity at 6 weeks, with associated changes in enteroendocrine responses, microbial ecology, and donor-microbe engraftment	Supports rational combination strategies integrating FMT with dietary substrates to guide defined microbiota-based therapeutics for metabolic disease	[398]
Low-FODMAP diet in IBS (NCT02107625)	Irritable bowel syndrome	Low-FODMAP diet vs traditional IBS dietary advice	Multicenter, randomized, single-blind, parallel-group trial (*n* = 75)	N/A	Symptom alleviation through the questionnaire IBS-SSS at 4 weeks	A diet low in FODMAPs reduced IBS symptoms as well as traditional IBS dietary advice. And subsequent fecal bacterial profiling showed that low-FODMAP diet altered bacterial profiles and that microbiota patterns were associated with dietary response	Supports dietary counselling as a feasible IBS management strategy and highlights the potential to integrate microbiota profiling with individualized nutritional guidance	[399, 400]
*Bifidobacterium longum* CECT 7347 (ES1) in IBS-D (NCT05339243)	Irritable bowel syndrome with diarrhea	Live or heat-treated ES1 vs placebo	Randomized double-blind, placebo-controlled trial (*n* = 200)	N/A	Change in total IBS-Symptom Severity Scale (IBS-SSS) score at 12 weeks	Both live ES1 and heat-treated ES1 significantly reduced IBS symptom severity, with improvements in stool consistency, quality of life, abdominal pain and anxiety scores	Supports strain-specific probiotic and postbiotic approaches as standardized microbiota-modulating strategies for IBS-D symptom management	[401]
Probiotic therapy for prevention of hepatic encephalopathy (NCT01110447)	Hepatic encephalopathy	Probiotic VSL#3 vs placebo	Randomized, double-blind, placebo-controlled trial (*n* = 130)	PHASE2/PHASE3	Development of overt HE in 6 months	VSL#3 reduced the risk of hospitalization for HE, as well as Child-Turcotte-Pugh and model for end-stage liver disease scores in patients with cirrhosis	Supports probiotic modulation of the gut–liver–brain axis as a feasible adjunctive strategy to reduce clinically relevant complications in cirrhosis	[402]
Synbiotic therapy for NAFLD (NCT01680640)	Non-alcoholic fatty liver disease	Synbiotic vs placebo	Randomized, double-blind, placebo-controlled trial (*n* = 104)	PHASE2	Change in liver fat, fibrosis scores, and the composition of the fecal microbiome at 12 months	Synbiotic supplementation altered the fecal microbiome but did not reduce liver fat content or fibrosis biomarkers	Provides clinical evidence that synbiotics can modulate the gut microbiota in NAFLD, informing the design of more targeted microbiota-based strategies with functionally relevant liver and inflammatory endpoints	[403]
FMT for Parkinson’s disease (NCT03808389)	Parkinson's disease	Donor FMT vs autologous FMT	Randomized, double-blind, placebo-controlled trial (*n* = 46)	PHASE2	Changes in clinical symptoms as scored on the MDS-UPDRS (Movement Disorder Society—Unified Parkinson's Disease Rating Scale) at 12 months	Healthy-donor FMT produced a greater improvement in MDS-UPDRS motor score than autologous FMT, with adverse events limited mainly to transient abdominal discomfort	Provides early clinical evidence for microbiome modulation in Parkinson’s disease and supports further evaluation of FMT across larger and more diverse patient cohorts	[404]
Adjunctive probiotic in depression (NCT03893162)	Major depressive disorder	Multi-strain probiotic “BioKult” vs placebo	Randomized, double-blind, placebo-controlled trial (*n* = 50)	N/A	Retention, acceptability, tolerability, and depressive/anxiety symptom scores at 8 weeks	Adjunctive probiotic treatment was acceptable and well tolerated, with improvements in depressive and anxiety symptoms	Supports microbiota–gut–brain axis modulation as a feasible adjunctive strategy for depression	[405]
Engineered microbial therapy (SYNB1618/SYNB1934) for phenylketonuria (NCT04534842)	Phenylketonuria	Engineered *E. coli* Nissle synthetic biotics SYNB1618 or SYNB1934	Open-label dose-escalation trial (*n* = 20)	PHASE2	Changes from baseline in labeled Phe (D5-Phe) in plasma at day 14	Both SYNB1618 and SYNB1934 reduced plasma Phe levels, and SYNB1934 also reduced fasting plasma Phe, with no serious adverse events or infections	Supports the clinical translation of engineered probiotics as programmable, standardized microbiota-based therapeutics for metabolic disease	[406]

This table summarizes representative completed microbiota-targeted clinical trials across different disease contexts and intervention types, providing an overview of their clinical outcomes, main findings, and translational implications

#### Dietary modulation

Dietary modulation is one of the most common and readily applicable microbiota-targeted interventions [407–409]. By changing luminal nutrient availability, diet can reshape microbial composition and function, thereby influencing intestinal barrier integrity, immune homeostasis, and microbiota-related metabolic pathways involved in disease [407–409]. Consistent with this, a recent large-scale population-based metagenomic study further demonstrated that diet is strongly associated with gut microbial composition, diversity, and functional pathways, and highlighted its potential to guide personalized nutritional intervention [410].

Dietary intervention has attracted increasing attention in diseases associated with gut microbial dysbiosis [411, 412]. In IBD, studies have shown that enteral nutrition, specific carbohydrate restriction, and dietary patterns characterized by high fiber intake and low consumption of processed foods may help alleviate intestinal inflammation and are accompanied by changes in microbial composition and metabolic profiles [413, 414]. In IBS, a diet low in fermentable oligosaccharides, disaccharides, monosaccharides, and polyols (FODMAP) can reduce fermentable substrate load and relieve bloating, abdominal pain, and altered bowel habits [415, 416]. In metabolic disorders, healthy dietary patterns such as the Mediterranean diet are generally associated with greater microbial diversity, enrichment of SCFA-producing bacteria, reduced inflammatory tone, and improvements in insulin sensitivity, lipid metabolism, and energy homeostasis [417–419]. This suggests that dietary intervention may have therapeutic effects not only by changing microbial composition, but also by reshaping microbial function and host metabolic responses. Functional dietary components such as resistant starch, polyphenols, and dietary fiber may further enhance the microbiota-modulating effects by promoting SCFA production and regulating metabolic pathways involving bile acids and tryptophan [420–422]. Overall, dietary modulation represents an accessible microbiota-targeted strategy with considerable translational potential. However, its clinical effects are still shaped by interindividual variability, dietary adherence, and disease context.

#### Probiotics, prebiotics, synbiotics, and postbiotics

Probiotics, prebiotics, synbiotics, and postbiotics represent widely used biotic and biotic-derived microbiota-targeted interventions [423, 424]. Compared with overall dietary management, these approaches generally have more clearly defined components, more standardized modes of administration, and greater feasibility for clinical implementation. For this reason, they have been widely studied in gastrointestinal, metabolic, and certain extraintestinal diseases [423, 424].

Probiotics are live microorganisms that, when administered in adequate amounts, confer a health benefit on the host, with commonly used genera such as *Lactobacillus* and *Bifidobacterium* [425]. Their most established clinical applications are in diarrhea-related conditions, particularly antibiotic-associated diarrhea and some post-infectious functional gastrointestinal disorders [426, 427]. In IBD, some probiotic formulations have shown potential for maintaining remission in mild-to-moderate UC, whereas their efficacy in CD appears more limited [428, 429]. In IBS, probiotics may also help alleviate symptoms such as bloating, abdominal pain, and altered bowel habits, although their effects vary considerably across strains and formulations [430].

Prebiotics are substrates that are selectively utilized by host microorganisms and confer health benefits [431]. Common examples include inulin, fructooligosaccharides, galactooligosaccharides, resistant starch, and other fermentable dietary fibers [431, 432]. By promoting beneficial microbes such as Bifidobacterium, prebiotics can improve the intestinal microenvironment and support bowel and metabolic function [431, 432]. In individuals with constipation or irregular bowel habits, certain prebiotic formulations may increase stool frequency and improve stool consistency [433]. In metabolic disorders, prebiotics have been investigated as adjunctive approaches for improving glucose and lipid metabolism and reducing low-grade inflammation [434, 435]. Their effects, however, vary substantially among individuals, and in patients with IBS, some highly fermentable substrates may exacerbate symptoms such as bloating.

Synbiotics combine probiotics and prebiotics with the aim of enhancing the colonization, survival, and functional activity of beneficial microbes by providing functional strains together with their growth substrates, which may generate synergistic effects [436]. They have been investigated in diseases such as IBD, IBS, MASLD, and metabolic syndrome, with some studies suggesting improvements in symptom scores, inflammatory markers, or metabolic parameters [437, 438]. Before broader clinical use, however, stronger evidence is still needed, especially because of the substantial heterogeneity in strain combinations, substrate composition, and intervention duration across studies.

Postbiotics are preparations of inanimate microorganisms and/or their components that confer a health benefit on the host [439]. Compared with live biotic preparations, postbiotics generally offer advantages in stability and standardization and may be particularly attractive in settings where the use of live microorganisms raises safety concerns [440]. Postbiotics have shown promise in areas such as infant nutrition, intestinal inflammation, infection prevention, and metabolic intervention, although they remain at a relatively early stage of translation into standardized clinical application [441–443].

Taken together, these approaches offer a more targeted way to modulate microbial composition and function than broad dietary approaches. Their clinical translation will depend on stronger evidence, standardized formulations, and better alignment among specific products, disease contexts, and patient populations.

#### Fecal Microbiota Transplantation (FMT)

Fecal microbiota transplantation (FMT) refers to the transfer of gut microbial communities and their associated components from rigorously screened healthy donors to recipients, with the aim of restoring microbial homeostasis, reinforcing colonization resistance, and modulating host immune and metabolic functions [444, 445]. Compared with relatively mild interventions such as dietary modulation, probiotics, and prebiotics, FMT provides a more direct and comprehensive reconstruction of a disrupted gut ecosystem [446]. As donor screening, manufacturing protocols, delivery routes, and quality control have improved, FMT has evolved from an empirical procedure into a more regulated microbiome-based therapeutic strategy across multiple diseases [444, 445, 447].

At present, the strongest evidence and clearest clinical application of FMT remain in recurrent *Clostridioides difficile* infection (rCDI). FMT can markedly reduce recurrence risk by restoring microbial diversity, reconstituting SCFA and bile acid metabolism, reinforcing colonization resistance, and suppressing pathogen expansion [448, 449]. The 2024 AGA guideline has incorporated fecal microbiota-based therapies in the management of recurrent CDI [447], and the U.S. FDA has approved REBYOTA and VOWST for prevention of recurrence following antibacterial treatment [450].

FMT has also been actively explored in IBD, particularly UC. In some patients, FMT can induce clinical remission and is accompanied by remodeling of microbial composition and function [451–453]. However, the durability of response, long-term maintenance effects, and improvements in endoscopic and histological outcomes remain inconsistent across studies [454, 455]. Heterogeneity in donors, recipients, and treatment protocols further limits cross-study comparability [454, 455]. Accordingly, FMT is not yet considered part of standard therapy for UC [447].

Beyond gastrointestinal diseases, FMT has been explored in several extra-intestinal conditions. In hepatic encephalopathy, FMT has been associated with improved cognitive function and related clinical outcomes in patients with cirrhosis, supporting a therapeutic role through modulation of the gut–liver–brain axis [456, 457]. FMT has also been explored in acute gastrointestinal graft-versus-host disease, where it may improve clinical manifestations and promote microbiota restoration in some steroid-refractory patients [458, 459]. In addition, FMT has been investigated as a strategy to improve insulin resistance and cardiometabolic abnormalities, although this application remains largely exploratory [398, 460].

In oncology, FMT has emerged as a potential strategy to enhance responsiveness to immune checkpoint inhibitors. In advanced melanoma, FMT from immunotherapy responders can restore anti-PD-1 responsiveness in a subset of resistant patients, alongside remodeling of the gut microbiota and tumor immune microenvironment [393, 461]. Its application is being extended to other solid tumors, including gastrointestinal cancers, reflecting the potential of microbiota remodeling in precision oncology [462].

Overall, FMT has clear clinical value in rCDI and broader therapeutic potential in selected disease settings. Although broader clinical implementation still requires progress in standardization, management of interindividual response variability, and safety evaluation, FMT is becoming increasingly established as a microbiota-based intervention.

#### Emerging precision microbiota therapies

With advances in microbiota-targeted therapeutics, gut microbiota interventions are shifting from broad microbial modulation toward more precise, controllable, and standardized strategies [11, 463]. Emerging precision microbiota therapies emphasize defined composition, focused targets, quality control, and reproducibility, making them better aligned with current drug development and clinical translation [463, 464].

Defined microbial consortia represent a characteristic form of emerging precision microbiota therapies [465]. Composed of selected commensal strains, they are designed to restore key microbiota functions with greater reproducibility [465]. rCDI remains the most mature clinical setting for this strategy, with VE303 serving as a representative example [387, 466]. This approach is also being explored in chronic inflammatory diseases, as illustrated by the clinical development of VE202 for UC [467]. In addition, selected commensal consortia may enable the targeted suppression of pathobionts such as *Enterobacteriaceae*, supporting their potential application in decolonization of antimicrobial-resistant organisms and in microbiota states associated with increased risk of infection or other adverse clinical outcomes [468].

In parallel, engineered live biotherapeutics further exemplify the programmable nature of microbiota-based therapy [469]. These strategies involve the modification of commensal or probiotic chassis to perform predefined therapeutic functions in the gut [469]. In phenylketonuria, SYNB1618 and its optimized successor SYNB1934 are both engineered *Escherichia coli* Nissle strains designed to metabolize phenylalanine in the intestinal lumen [406, 470]. These candidates represent examples of clinical translation in this field, highlighting the shift of engineered bacteria from proof-of-concept studies toward indication-driven clinical development [406, 470]. Beyond inherited metabolic disorders, engineered bacteria are being developed for inflammatory diseases, infections, and cancer [469, 471].

In addition, phage therapy and CRISPR-mediated microbiome editing offer new strategies for the targeted elimination of specific pathogens or pathobionts [472, 473]. Unlike broad-spectrum antibiotics, these approaches may selectively suppress pathobionts, antimicrobial-resistant organisms, or strains carrying specific virulence determinants while minimizing disruption to the broader commensal microbiota [472–474]. At the same time, personalized microbiota-directed interventions targeting specific metabolic or ecological functional deficits represent another important direction [475, 476]. Rather than merely altering microbial composition, these approaches aim to restore key microbiota-related functions more precisely according to the patient’s microbial profile, metabolic features, and disease context [475, 476].

Defined microbial consortia, engineered live biotherapeutics, phage- or CRISPR-based microbiome editing, and function-oriented personalized interventions together form the main framework of emerging precision microbiota therapies. Although most of these strategies remain at the preclinical or early clinical stage, their development marks a gradual shift from empirical microbiota modulation toward more precise, standardized, and clinically translatable therapeutic approaches.

### Challenges and future perspectives in clinical translation

Although considerable progress has been made in elucidating disease mechanisms, developing microbiota-based biomarkers, and exploring microbiota-targeted interventions, the clinical translation of gut microbiota research still faces substantial challenges [11, 477] (Fig. 6). The key bottlenecks are no longer limited to identifying disease-associated microbial alterations, but lie in whether these findings have robust causal interpretability, are reproducible across populations and platforms, can be translated into standardized diagnostic or therapeutic strategies, and meet requirements for long-term safety and regulatory feasibility [11, 369, 478].

First, insufficient evidence for causality remains a major barrier to the clinical translation of gut microbiota research. Many existing studies, including those on CRC, IBD, and various extraintestinal diseases, are still based primarily on cross-sectional analyses or case–control comparisons, making it difficult to determine whether specific microbial alterations are drivers of disease, consequences of the disease state, or secondary changes related to diet, medication exposure, inflammation, or host behavior [478, 479]. This limitation not only weakens the robustness of microbiota-based biomarkers, but also increases the risk of target misidentification in microbiota-directed interventions [11, 478]. To address this issue, recent studies have increasingly emphasized multicenter, large-scale, longitudinal prospective cohorts, together with functional validation of candidate strains and metabolites using germ-free animal models, colonization models, culturomics, functional metagenomics, and metabolite complementation [11, 478, 480, 481]. Causal inference approaches, such as Mendelian randomization, are also being introduced into population-based studies to prioritize microbial features with greater potential pathogenic or protective relevance [479]. Together, these approaches are important for moving the field from association-based observations toward more actionable microbiome-based medicine [478].

In addition to causal uncertainty, marked interindividual heterogeneity and the lack of methodological standardization further limit the reproducibility and clinical generalizability of gut microbiota research. Age, genetic background, dietary patterns, lifestyle, medication exposure, and host immune status can all shape the baseline microbial ecosystem, causing the same microbial signal or intervention to perform differently across individuals [11, 369, 477]. Technical variation in sample collection, storage, DNA extraction, sequencing platforms, and bioinformatic workflows also compromises cross-study consistency [369, 482, 483]. To address this, recent international consensus statements, reporting guidelines, and reference materials have begun to promote a more standardized framework for microbiome testing, with increasing emphasis on both technical harmonization and standardized interpretation [369, 482, 483]. At the same time, growing efforts are being made to stratify patients according to baseline microbiota profiles, metabolomic features, and immune phenotypes, rather than relying on a universal standard [477, 484, 485]. These developments suggest that gut microbiota-based medicine is more likely to enter clinical practice through stratified and individualized approaches rather than universal application.

Beyond issues of evidence and standardization, current microbiota-based interventions still face major limitations, including colonization resistance, limited durability, and insufficient long-term safety evaluation. Although FMT has established clinical value in rCDI, its broader use in non-infectious indications remains constrained by unresolved issues related to donor screening, long-term safety, and potential transmission of antimicrobial resistance genes [486]. Likewise, many probiotics, prebiotics, and related products fail to achieve stable engraftment or durable functional effects, and the ecological effects of some substrates may be broader and less specific than intended [487, 488]. Emerging precision approaches, such as phages, engineered bacteria, and metabolite-based therapies, offer greater specificity, but their in vivo efficacy remains constrained by challenges related to stability, delivery, host compatibility, and potential long-term ecological effects [489]. In response, recent efforts have focused on developing next-generation probiotics with improved ecological fitness, optimizing delivery strategies such as sporulation, encapsulation, and sustained-release formulations, and exploring combination-based reconstruction approaches to enhance the durability and controllability of microbiota-directed interventions [469, 490]. In parallel, long-term follow-up registries and systematic assessment of delayed adverse events are increasingly recognized as important priorities [491].

More fundamentally, the gut microbiota exerts its effects through multilayered networks involving metabolism, immunity, barrier function, and neuroendocrine signaling, making single-taxon or single-pathway explanations and interventions inherently limited. This may explain why biomarker models based on individual microbes or metabolites often show limited external validity, and why interventions that alter microbial composition do not always yield consistent clinical benefit [485]. To address this limitation, recent research has increasingly emphasized multi-omics integration, combining metagenomics, metabolomics, proteomics, host transcriptomics, and immune phenotyping to identify functionally relevant nodes and regulatory pathways [492, 493]. Interpretable machine learning, causal inference, and network analysis are also being applied to improve the integration of complex multimodal data [494, 495]. These advances suggest that future microbiota-based medicine will likely depend less on simply adding or removing specific microbes and more on the precise and dynamic modulation of key functional networks.

In this context, progress in causal validation, methodological standardization, safety assessment, and precision intervention will be essential for translating gut microbiota research into clinically useful diagnostics and therapeutics.

## Conclusion

The gut microbiota is now recognized as an important regulator of human health and disease, linking intestinal ecology to host metabolism, barrier integrity, immunity, and systemic organ function. Rather than acting through isolated taxa or single pathways, its effects are exerted through interconnected functional networks that can maintain homeostasis or, when disrupted, contribute to disease development and progression across both intestinal and extra-intestinal systems. The studies reviewed here indicate that microbiota dysbiosis is not simply a compositional abnormality, but a broader disturbance of host–microbe interactions with important mechanistic and clinical implications.

The field is also shifting from descriptive profiling toward clinical translation. Microbiota-based biomarkers, dietary modulation, biotic products, fecal microbiota transplantation, and emerging precision microbiota therapies are broadening the clinical application of microbiome research. Even so, several barriers still limit translation, including unresolved causality, marked interindividual heterogeneity, insufficient methodological standardization, and the need for durable efficacy and long-term safety evaluation. Future progress will likely depend on integrating longitudinal human studies with functional validation, multi-omics analysis, and more precise intervention strategies that target microbial functions and host–microbe networks rather than composition alone. Taken together, the gut microbiota represents not only an important determinant of health and pathophysiology, but also a promising entry point for more precise, mechanism-informed diagnostics and therapeutics.

## Data Availability

No new data were generated in this study. Data used to generate Fig. 5 were retrieved from the publicly accessible ClinicalTrials.gov database.

## Abbreviations

3-sucCA: 3-Succinylated cholic acid
4-EPS: 4-Ethylphenyl sulfate
5-AVA: 5-Aminovaleric acid
5-HT: 5-Hydroxytryptamine
AhR: Aryl hydrocarbon receptor
AIEC: *adherent-invasive Escherichia coli*
ALPK1: Alpha-kinase 1
AMP: Antimicrobial peptide
*APC*: *Adenomatous Polyposis Coli*
ASD: Autism spectrum disorder
BCAA: Branched-chain amino acid
BCL2: B-cell lymphoma-2
BFT: *Bacteroides fragilis* Toxin
CD: Crohn’s disease
CDT: Cytolethal distending toxin
CEACAM1: Carcinoembryonic antigen-related cell adhesion molecule 1
CRC: Colorectal cancer
CYP7A1: Cholesterol 7α-hydroxylase
DC: Immature dendritic cell
*E. coli*: *Escherichia coli*
EEC: Enteroendocrine cell
EMT: Epithelial–mesenchymal transition
ERK: Extracellular signal-regulated kinase
ETBF: Enterotoxigenic *Bacteroides fragilis*
*F. nucleatum*: *Fusobacterium nucleatum*
FadA: *Fusobacterium* Adhesin A
FFAR2: Free fatty acid receptor 2
FGF19: Fibroblast growth factor 19
FMT: Fecal microbiota transplantation
FODMAP: Fermentable oligosaccharides, disaccharides, monosaccharides, and polyols
FXR: Farnesoid X receptor
GABA: γ-Aminobutyric acid
Gal-GalNAc: Galactose-N-acetyl-D-galactosamine
GALT: Gut-associated lymphoid tissue
GIP: Glucose-dependent insulinotropic polypeptide
GLP-1: Glucagon-like peptide-1
GPCR: G protein-coupled receptor
GSDME: Gasdermin E
HDAC: Histone deacetylase
HDL: High-density lipoprotein
HIF-1α: Hypoxia-inducible factor 1α
HPA: Hypothalamic–pituitary–adrenal
HPG: Hypothalamic–pituitary–gonadal
HPT: Hypothalamic–pituitary–thyroid
IAA: Indole acetic acid
IBD: Inflammatory bowel disease
IBS: Irritable bowel syndrome
IBS-C: Constipation-predominant IBS
IBS-D: Diarrhea-predominant IBS
ICA: Indole-3-carboxylic acid
ICAM1: Intercellular adhesion molecule 1
IDO: Indoleamine 2,3-dioxygenase
IL-22: Interleukin-22
ILA: Indole-3-lactic acid
ILC: Innate lymphoid cell
IPA: Indole-3-propionic acid
ISC: Intestinal stem cells
KLF4: Krüppel-like factor 4
LBP: LPS-binding protein
LCA: Lithocholic acid
LPS: Lipopolysaccharides
LRRC19: Leucine-rich repeat containing 19
MAMP: Microbe-associated molecular pattern
MASH: Metabolic dysfunction-associated steatohepatitis
MASLD: Metabolic dysfunction-associated steatotic liver disease
MCT1: Monocarboxylate transporter 1
MDD: Major depressive disorder
MDSC: Myeloid-derived suppressor cell
MGBA: Microbiota-gut-brain axis
miR: MicroRNA
MUC2: Mucin 2
NAFLD: Nonalcoholic fatty liver disease
NAM: N-acetylmuramic acid
NF-κB: Nuclear factor kappa B
NK: Natural killer cell
NLR: NOD-like receptor
PAMP: Pathogen-associated molecular pattern
pCS: P-cresol sulfate
PCWBR2: Putative cell wall binding repeat 2
PD: Parkinson’s disease
PD-1: Programmed cell death 1
PI3K: Phosphoinositide 3-kinase
PRR: Pattern recognition receptor
PSC: Primary sclerosing cholangitis
PYY: Peptide YY
RadD: Radiation resistance protein D
RAS: Rat sarcoma viral oncogene
rCDI: Recurrent *Clostridioides difficile* infection
ROS: Reactive oxygen species
SBA: Secondary bile acid
SCFA: Short-chain fatty acids
SFB: segmented filamentous bacteria
sIgA: Secretory IgA
Siglec: Sialic-acid-binding immunoglobulin-like lectin
SLC26A3: Solute carrier family 26 member 3
STAT3: Signal transducer and activator of transcription 3
T2DM: Type 2 diabetes mellitus
TAK1: Transforming growth factor-β-activated kinase 1
TAM: Tumor-associated macrophage
TAN: Tumor-associated neutrophil
TGR5: Takeda G protein-coupled receptor 5
Th17: T helper 17 cell
THDOC: Tetrahydrodeoxycorticosterone
THP: Tetrahydroprogesterone
TIGHT: T-cell immunoglobulin and immunoreceptor tyrosine-based inhibitory motif domain
TLR: Toll-like receptor
TMA: Trimethylamine
TMAO: Trimethylamine N-oxide
TRAF6: Tumor necrosis factor receptor-associated factor 6
Treg: Regulatory T cell
UC: Ulcerative colitis
UDCA: Ursodeoxycholic acid
YAP: Yes-associated protein

## References

[1] Hou K, Wu ZX, Chen XY, Wang JQ, Zhang D, Xiao C, et al. Microbiota in health and diseases. Signal Transduction Targeted Ther. 2022;7:135. 10.1038/s41392-022-00974-4. 10.1038/s41392-022-00974-4 PMC9034083 35461318

[2] Ma Z, Zuo T, Frey N, Rangrez AY. A systematic framework for understanding the microbiome in human health and disease: from basic principles to clinical translation. Signal Transduction Targeted Ther. 2024;9(1):237. 10.1038/s41392-024-01946-6. 10.1038/s41392-024-01946-6 PMC11418828 39307902

[3] de Vos WM, Tilg H, Van Hul M, Cani PD. Gut microbiome and health: mechanistic insights. Gut. 2022;71(5):1020–32. 10.1136/gutjnl-2021-326789. 35105664 10.1136/gutjnl-2021-326789 PMC8995832

[4] Tanca A, Palomba A, Fiorito G, Abbondio M, Pagnozzi D, Uzzau S. Metaproteomic portrait of the healthy human gut microbiota. npj Biofilms Microbiomes. 2024;10(1):54. 10.1038/s41522-024-00526-4. 10.1038/s41522-024-00526-4 PMC11214629 38944645

[5] Van Hul M, Cani PD, Petitfils C, De Vos WM, Tilg H, El-Omar EM. What defines a healthy gut microbiome? Gut. 2024;73(11):e333378. 10.1136/gutjnl-2024-333378. 39322314 10.1136/gutjnl-2024-333378 PMC11503168

[6] Joos R, Boucher K, Lavelle A, Arumugam M, Blaser MJ, Claesson MJ, et al. Examining the healthy human microbiome concept. Nat Rev Microbiol. 2025;23(3):192–205. 10.1038/s41579-024-01107-0. 39443812 10.1038/s41579-024-01107-0

[7] Hrncir T. Gut microbiota dysbiosis: triggers, consequences, diagnostic and therapeutic options. Microorganisms. 2022;10(3):578. 10.3390/microorganisms10030578. 35336153 10.3390/microorganisms10030578 PMC8954387

[8] Ni J, Wu GD, Albenberg L, Tomov VT. Gut microbiota and IBD: causation or correlation? Nat Rev, Gastroenterol Hepatol. 2017;14(10):573–84. 10.1038/nrgastro.2017.88. 28743984 10.1038/nrgastro.2017.88 PMC5880536

[9] Grant ET, Monzel E, Desai MS. Navigating the duality of akkermansia muciniphila. Nat Microbiol. 2026;11(1):20–30. 10.1038/s41564-025-02222-1. 41492068 10.1038/s41564-025-02222-1

[10] Zuo S, Ma J, Li X, Fan Z, Li X, Luo Y, et al. The dual role of gut microbiota and their metabolites in hepatocellular carcinoma: a context-dependent framework. Microorganisms. 2025;14(1):73. 10.3390/microorganisms14010073. 41597593 10.3390/microorganisms14010073 PMC12844408

[11] Porcari S, Ng SC, Zitvogel L, Sokol H, Weersma RK, Elinav E, et al. The microbiome for clinicians. Cell. 2025;188(11):2836–44. 10.1016/j.cell.2025.04.016. 40446358 10.1016/j.cell.2025.04.016

[12] Wu G, Xu T, Zhao N, Lam YY, Ding X, Wei D, et al. A core microbiome signature as an indicator of health. Cell. 2024;187(23):6550-6565.e11. 10.1016/j.cell.2024.09.019. 39378879 10.1016/j.cell.2024.09.019

[13] Yatsunenko T, Rey FE, Manary MJ, Trehan I, Dominguez-Bello MG, Contreras M, et al. Human gut microbiome viewed across age and geography. Nature. 2012;486(7402):222–7. 10.1038/nature11053. 22699611 10.1038/nature11053 PMC3376388

[14] Hoegenauer C, Hammer HF, Mahnert A, Moissl-Eichinger C. Methanogenic archaea in the human gastrointestinal tract. Nat Rev, Gastroenterol Hepatol. 2022;19(12):805–13. 10.1038/s41575-022-00673-z. 36050385 10.1038/s41575-022-00673-z

[15] Berg G, Rybakova D, Fischer D, Cernava T, Vergès MCC, Charles T, et al. Microbiome definition re-visited: old concepts and new challenges. Microbiome. 2020;8:103. 10.1186/s40168-020-00875-0. 32605663 10.1186/s40168-020-00875-0 PMC7329523

[16] Cani PD. Human gut microbiome: hopes, threats and promises. Gut. 2018;67(9):1716–25. 10.1136/gutjnl-2018-316723. 29934437 10.1136/gutjnl-2018-316723 PMC6109275

[17] Lozupone CA, Stombaugh JI, Gordon JI, Jansson JK, Knight R. Diversity, stability and resilience of the human gut microbiota. Nature. 2012;489(7415):220–30. 10.1038/nature11550. 22972295 10.1038/nature11550 PMC3577372

[18] Moya A, Ferrer M. Functional redundancy-induced stability of gut microbiota subjected to disturbance. Trends Microbiol. 2016;24(5):402–13. 10.1016/j.tim.2016.02.002. 26996765 10.1016/j.tim.2016.02.002

[19] Tian L, Wang XW, Wu AK, Fan Y, Friedman J, Dahlin A, et al. Deciphering functional redundancy in the human microbiome. Nat Commun. 2020;11(1):6217. 10.1038/s41467-020-19940-1. 33277504 10.1038/s41467-020-19940-1 PMC7719190

[20] Fassarella M, Blaak EE, Penders J, Nauta A, Smidt H, Zoetendal EG. Gut microbiome stability and resilience: elucidating the response to perturbations in order to modulate gut health. Gut. 2021;70(3):595–605. 10.1136/gutjnl-2020-321747. 33051190 10.1136/gutjnl-2020-321747

[21] Fan Y, Pedersen O. Gut microbiota in human metabolic health and disease. Nat Rev Microbiol. 2021;19(1):55–71. 10.1038/s41579-020-0433-9. 32887946 10.1038/s41579-020-0433-9

[22] Mukhopadhya I, Louis P. Gut microbiota-derived short-chain fatty acids and their role in human health and disease. Nat Rev Microbiol. 2025;23(10):635–51. 10.1038/s41579-025-01183-w. 40360779 10.1038/s41579-025-01183-w

[23] Fagundes RR, Belt SC, Bakker BM, Dijkstra G, Harmsen HJM, Faber KN. Beyond butyrate: microbial fiber metabolism supporting colonic epithelial homeostasis. Trends Microbiol. 2024;32(2):178–89. 10.1016/j.tim.2023.07.014. 37596118 10.1016/j.tim.2023.07.014

[24] Arnoldini M, Sharma R, Moresi C, Chure G, Chabbey J, Slack E, et al. Quantifying the varying harvest of fermentation products from the human gut microbiota. Cell. 2025;188(19):5332-5342.e16. 10.1016/j.cell.2025.07.005. 40744013 10.1016/j.cell.2025.07.005 PMC12556654

[25] Donohoe DR, Garge N, Zhang X, Sun W, O’Connell TM, Bunger MK, et al. The microbiome and butyrate regulate energy metabolism and autophagy in the mammalian colon. Cell Metab. 2011;13(5):517–26. 10.1016/j.cmet.2011.02.018. 21531334 10.1016/j.cmet.2011.02.018 PMC3099420

[26] Nicholson JK, Holmes E, Kinross J, Burcelin R, Gibson G, Jia W, et al. Host-gut microbiota metabolic interactions. Science. 2012;336(6086):1262–7. 10.1126/science.1223813. 22674330 10.1126/science.1223813

[27] Cummings JH, Pomare EW, Branch WJ, Naylor CP, Macfarlane GT. Short chain fatty acids in human large intestine, portal, hepatic and venous blood. Gut. 1987;28(10):1221–7. 10.1136/gut.28.10.1221. 3678950 10.1136/gut.28.10.1221 PMC1433442

[28] LeBlanc JG, Milani C, de Giori GS, Sesma F, van Sinderen D, Ventura M. Bacteria as vitamin suppliers to their host: a gut microbiota perspective. Curr Opin Biotechnol. 2013;24(2):160–8. 10.1016/j.copbio.2012.08.005. 22940212 10.1016/j.copbio.2012.08.005

[29] Tarracchini C, Lordan C, Milani C, Moreira LPD, Alabedallat QM, de Moreno de LeBlanc A, et al. Vitamin biosynthesis in the gut: interplay between mammalian host and its resident microbiota. Microbiol Mol Biol Rev: MMBR. 2025;89(2):e0018423. 10.1128/mmbr.00184-23. 10.1128/mmbr.00184-23 PMC12188732 40172109

[30] Han Z, Zhao L, Hu Q, Hung I, Liu C, Liu S, et al. Gut microbiota-mediated modulation of host amino acid availability and metabolism. Gut Microbes. 2025;17(1):2552345. 10.1080/19490976.2025.2552345. 40878016 10.1080/19490976.2025.2552345 PMC12407847

[31] Cani PD, Van Hul M, Lefort C, Depommier C, Rastelli M, Everard A. Microbial regulation of organismal energy homeostasis. Nat Metab. 2019;1(1):34–46. 10.1038/s42255-018-0017-4. 32694818 10.1038/s42255-018-0017-4

[32] Kimura I, Ichimura A, Ohue-Kitano R, Igarashi M. Free fatty acid receptors in health and disease. Physiol Rev. 2020;100(1):171–210. 10.1152/physrev.00041.2018. 31487233 10.1152/physrev.00041.2018

[33] Lei J, Wang X, Liu X. Microbiota-derived metabolites in the epigenetic regulation of the host. Sci Bull. 2025;70(21):3667–78. 10.1016/j.scib.2025.09.030. 10.1016/j.scib.2025.09.030 41047314

[34] Collins SL, Stine JG, Bisanz JE, Okafor CD, Patterson AD. Bile acids and the gut microbiota: metabolic interactions and impacts on disease. Nat Rev Microbiol. 2023;21(4):236–47. 10.1038/s41579-022-00805-x. 36253479 10.1038/s41579-022-00805-x PMC12536349

[35] Lin S, Wang S, Wang P, Tang C, Wang Z, Chen L, et al. Bile acids and their receptors in regulation of gut health and diseases. Prog Lipid Res. 2023;89:101210. 10.1016/j.plipres.2022.101210. 36577494 10.1016/j.plipres.2022.101210

[36] Nie Q, Luo X, Wang K, Ding Y, Jia S, Zhao Q, et al. Gut symbionts alleviate MASH through a secondary bile acid biosynthetic pathway. Cell. 2024;187(11):2717-2734.e33. 10.1016/j.cell.2024.03.034. 38653239 10.1016/j.cell.2024.03.034

[37] Muñoz VR, Moreau F, Soto M, Watanabe Y, Pham LD, Zhong J, et al. Portal vein-enriched metabolites as intermediate regulators of the gut microbiome in insulin resistance. Cell Metab. 2025;37(10):2048-2065.e6. 10.1016/j.cmet.2025.08.005. 40914155 10.1016/j.cmet.2025.08.005 PMC12529558

[38] Neurath MF, Artis D, Becker C. The intestinal barrier: a pivotal role in health, inflammation, and cancer. Lancet Gastroenterol Hepatol. 2025;10(6):573–92. 10.1016/S2468-1253(24)00390-X. 40086468 10.1016/S2468-1253(24)00390-X

[39] Chen K, Wang H, Yang Y, Tang C, Sun X, Zhou J, et al. Common mechanisms of gut microbe-based strategies for the treatment of intestine-related diseases: Based on multi-target interactions with the intestinal barrier. Cell Commun Signal. 2025;23:288. 10.1186/s12964-025-02299-5. 40528179 10.1186/s12964-025-02299-5 PMC12175372

[40] Bilotta AJ, Ma C, Yang W, Yu Y, Yu Y, Zhao X, et al. Propionate enhances cell speed and persistence to promote intestinal epithelial turnover and repair. Cell Mol Gastroenterol Hepatol. 2021;11(4):1023–44. 10.1016/j.jcmgh.2020.11.011. 33238220 10.1016/j.jcmgh.2020.11.011 PMC7898181

[41] Ma L, Ni Y, Wang Z, Tu W, Ni L, Zhuge F, et al. Spermidine improves gut barrier integrity and gut microbiota function in diet-induced obese mice. Gut Microbes. 2020;12(1):1–19. 10.1080/19490976.2020.1832857. 33151120 10.1080/19490976.2020.1832857 PMC7668533

[42] Abdulqadir R, Al-Sadi R, Gupta Y, Rawat M, Ma T. Probiotic bacteria bifidobacterium bifidum upregulation of intestinal epithelial tight junction barrier is mediated by TLR-2/TLR-6 receptor complex activation of occludin gene. npj Biofilms Microbiomes. 2026;12(1):37. 10.1038/s41522-025-00903-7. 10.1038/s41522-025-00903-7 PMC12881538 41559082

[43] Paone P, Cani PD. Mucus barrier, mucins and gut microbiota: the expected slimy partners? Gut. 2020;69(12):2232–43. 10.1136/gutjnl-2020-322260. 32917747 10.1136/gutjnl-2020-322260 PMC7677487

[44] Luis AS, Hansson GC. Intestinal mucus and their glycans: A habitat for thriving microbiota. Cell Host Microbe. 2023;31(7):1087–100. 10.1016/j.chom.2023.05.026. 37442097 10.1016/j.chom.2023.05.026 PMC10348403

[45] Inaba R, Vujakovic S, Bergstrom K. The gut mucus network: a dynamic liaison between microbes and the immune system. Semin Immunol. 2023;69:101807. 10.1016/j.smim.2023.101807. 37478802 10.1016/j.smim.2023.101807

[46] Fu J, Zong X, Jin M, Min J, Wang F, Wang Y. Mechanisms and regulation of defensins in host defense. Signal Transduction Targeted Ther. 2023;8(1):300. 10.1038/s41392-023-01553-x. 10.1038/s41392-023-01553-x PMC10423725 37574471

[47] Okumura R, Takeda K. The role of the mucosal barrier system in maintaining gut symbiosis to prevent intestinal inflammation. Semin Immunopathol. 2024;47(1):2. 10.1007/s00281-024-01026-5. 39589551 10.1007/s00281-024-01026-5 PMC11599372

[48] Huus KE, Petersen C, Finlay BB. Diversity and dynamism of IgA-microbiota interactions. Nat Rev Immunol. 2021;21(8):514–25. 10.1038/s41577-021-00506-1. 33568782 10.1038/s41577-021-00506-1

[49] Hapfelmeier S, Lawson MAE, Slack E, Kirundi JK, Stoel M, Heikenwalder M, et al. Reversible microbial colonization of germ-free mice reveals the dynamics of IgA immune responses. Science. 2010;328(5986):1705–9. 10.1126/science.1188454. 20576892 10.1126/science.1188454 PMC3923373

[50] Caballero-Flores G, Pickard JM, Núñez G. Microbiota-mediated colonization resistance: mechanisms and regulation. Nat Rev Microbiol. 2023;21(6):347–60. 10.1038/s41579-022-00833-7. 36539611 10.1038/s41579-022-00833-7 PMC10249723

[51] Woelfel S, Silva MS, Stecher B. Intestinal colonization resistance in the context of environmental, host, and microbial determinants. Cell Host Microbe. 2024;32(6):820–36. 10.1016/j.chom.2024.05.002. 38870899 10.1016/j.chom.2024.05.002

[52] Spragge F, Bakkeren E, Jahn MT, B N Araujo E, Pearson CF, Wang X, et al. Microbiome diversity protects against pathogens by nutrient blocking. Science. 2023;382(6676):eadj3502. 10.1126/science.adj3502. 10.1126/science.adj3502 PMC7616675 38096285

[53] Deng L, Wang S. Colonization resistance: the role of gut microbiota in preventing salmonella invasion and infection. Gut Microbes. 2024;16(1):2424914. 10.1080/19490976.2024.2424914. 39514544 10.1080/19490976.2024.2424914 PMC11552263

[54] Heilbronner S, Krismer B, Brötz-Oesterhelt H, Peschel A. The microbiome-shaping roles of bacteriocins. Nat Rev Microbiol. 2021;19(11):726–39. 10.1038/s41579-021-00569-w. 34075213 10.1038/s41579-021-00569-w

[55] Knaus UG, Hertzberger R, Pircalabioru GG, Yousefi SPM, Branco Dos Santos F. Pathogen control at the intestinal mucosa - H_2_O_2_ to the rescue. Gut Microbes. 2017;8(1):67–74. 10.1080/19490976.2017.1279378. 28080210 10.1080/19490976.2017.1279378 PMC5341913

[56] Ducarmon QR, Zwittink RD, Hornung BVH, van Schaik W, Young VB, Kuijper EJ. Gut microbiota and colonization resistance against bacterial enteric infection. Microbiol mol biol rev: MMBR. 2019;83(3):e00007-19. 10.1128/MMBR.00007-19. 31167904 10.1128/MMBR.00007-19 PMC6710460

[57] Sorbara MT, Dubin K, Littmann ER, Moody TU, Fontana E, Seok R, et al. Inhibiting antibiotic-resistant enterobacteriaceae by microbiota-mediated intracellular acidification. J Exp Med. 2019;216(1):84–98. 10.1084/jem.20181639. 30563917 10.1084/jem.20181639 PMC6314524

[58] Zheng D, Liwinski T, Elinav E. Interaction between microbiota and immunity in health and disease. Cell Res. 2020;30(6):492–506. 10.1038/s41422-020-0332-7. 32433595 10.1038/s41422-020-0332-7 PMC7264227

[59] Gensollen T, Iyer SS, Kasper DL, Blumberg RS. How colonization by microbiota in early life shapes the immune system. Sci (N Y NY). 2016;352(6285):539–44. 10.1126/science.aad9378. 10.1126/science.aad9378 PMC5050524 27126036

[60] Donald K, Finlay BB. Early-life interactions between the microbiota and immune system: impact on immune system development and atopic disease. Nat Rev Immunol. 2023;23(11):735–48. 10.1038/s41577-023-00874-w. 37138015 10.1038/s41577-023-00874-w

[61] Bemark M, Pitcher MJ, Dionisi C, Spencer J. Gut-associated lymphoid tissue: a microbiota-driven hub of B cell immunity. Trends Immunol. 2024;45(3):211–23. 10.1016/j.it.2024.01.006. 38402045 10.1016/j.it.2024.01.006 PMC11227984

[62] Ivanov II, Atarashi K, Manel N, Brodie EL, Shima T, Karaoz U, et al. Induction of intestinal Th17 cells by segmented filamentous bacteria. Cell. 2009;139(3):485–98. 10.1016/j.cell.2009.09.033. 19836068 10.1016/j.cell.2009.09.033 PMC2796826

[63] Goto Y, Panea C, Nakato G, Cebula A, Lee C, Diez MG, et al. Segmented filamentous bacteria antigens presented by intestinal dendritic cells drive mucosal Th17 cell differentiation. Immunity. 2014;40(4):594–607. 10.1016/j.immuni.2014.03.005. 24684957 10.1016/j.immuni.2014.03.005 PMC4084624

[64] Henrick BM, Rodriguez L, Lakshmikanth T, Pou C, Henckel E, Arzoomand A, et al. Bifidobacteria-mediated immune system imprinting early in life. Cell. 2021;184(15):3884-3898.e11. 10.1016/j.cell.2021.05.030. 34143954 10.1016/j.cell.2021.05.030

[65] Smith PM, Howitt MR, Panikov N, Michaud M, Gallini CA, Bohlooly-Y M, et al. The microbial metabolites, short-chain fatty acids, regulate colonic treg cell homeostasis. Science. 2013;341(6145):569–73. 10.1126/science.1241165. 23828891 10.1126/science.1241165 PMC3807819

[66] Furusawa Y, Obata Y, Fukuda S, Endo TA, Nakato G, Takahashi D, et al. Commensal microbe-derived butyrate induces the differentiation of colonic regulatory T cells. Nature. 2013;504(7480):446–50. 10.1038/nature12721. 24226770 10.1038/nature12721

[67] Schulthess J, Pandey S, Capitani M, Rue-Albrecht KC, Arnold I, Franchini F, et al. The short chain fatty acid butyrate imprints an antimicrobial program in macrophages. Immunity. 2019;50(2):432-445.e7. 10.1016/j.immuni.2018.12.018. 30683619 10.1016/j.immuni.2018.12.018 PMC6382411

[68] Chang PV, Hao L, Offermanns S, Medzhitov R. The microbial metabolite butyrate regulates intestinal macrophage function via histone deacetylase inhibition. Proc Natl Acad Sci U S A. 2014;111(6):2247–52. 10.1073/pnas.1322269111. 24390544 10.1073/pnas.1322269111 PMC3926023

[69] Seo SK, Kwon B. Immune regulation through tryptophan metabolism. Exp Mol Med. 2023;55(7):1371–9. 10.1038/s12276-023-01028-7. 37394584 10.1038/s12276-023-01028-7 PMC10394086

[70] Zelante T, Iannitti RG, Cunha C, De Luca A, Giovannini G, Pieraccini G, et al. Tryptophan catabolites from microbiota engage aryl hydrocarbon receptor and balance mucosal reactivity via interleukin-22. Immunity. 2013;39(2):372–85. 10.1016/j.immuni.2013.08.003. 23973224 10.1016/j.immuni.2013.08.003

[71] Cai J, Sun L, Gonzalez FJ. Gut microbiota-derived bile acids in intestinal immunity, inflammation, and tumorigenesis. Cell Host Microbe. 2022;30(3):289–300. 10.1016/j.chom.2022.02.004. 35271802 10.1016/j.chom.2022.02.004 PMC8923532

[72] Fleishman JS, Kumar S. Bile acid metabolism and signaling in health and disease: molecular mechanisms and therapeutic targets. Signal Transduction Targeted Ther. 2024;9(1):97. 10.1038/s41392-024-01811-6. 10.1038/s41392-024-01811-6 PMC11045871 38664391

[73] Dong X, Qi M, Cai C, Zhu Y, Li Y, Coulter S, et al. Farnesoid X receptor mediates macrophage-intrinsic responses to suppress colitis-induced colon cancer progression. JCI Insight. 2024;9(2):e170428. 10.1172/jci.insight.170428. 38258906 10.1172/jci.insight.170428 PMC10906220

[74] Lin X, Yu Z, Liu Y, Li C, Hu H, Hu JC, et al. Gut–X axis. iMeta. 2025;4(1):e270. 10.1002/imt2.270. 10.1002/imt2.270 PMC11865426 40027477

[75] Ahlawat S, Asha N, Sharma KK. Gut-organ axis: a microbial outreach and networking. Lett Appl Microbiol. 2021;72(6):636–68. 10.1111/lam.13333. 32472555 10.1111/lam.13333

[76] Pant K, Venugopal SK, Lorenzo Pisarello MJ, Gradilone SA. The role of gut microbiome-derived short-chain fatty acid butyrate in hepatobiliary diseases. Am J Pathol. 2023;193(10):1455–67. 10.1016/j.ajpath.2023.06.007. 37422149 10.1016/j.ajpath.2023.06.007 PMC10548274

[77] Yoshida H, Ishii M, Akagawa M. Propionate suppresses hepatic gluconeogenesis via GPR43/AMPK signaling pathway. Arch Biochem Biophys. 2019;672:108057. 10.1016/j.abb.2019.07.022. 31356781 10.1016/j.abb.2019.07.022

[78] Maruyama D, Tian X, Doan TNM, Liao WI, Chaki T, Taenaka H, et al. Gut microbiome-derived propionate reprograms alveolar macrophages metabolically and regulates lung injury responses in mice. Gut Microbes. 2026;18(1):2606486. 10.1080/19490976.2025.2606486. 41467904 10.1080/19490976.2025.2606486 PMC12758369

[79] Pluznick JL, Protzko RJ, Gevorgyan H, Peterlin Z, Sipos A, Han J, et al. Olfactory receptor responding to gut microbiota-derived signals plays a role in renin secretion and blood pressure regulation. Proc Natl Acad Sci U S A. 2013;110(11):4410–5. 10.1073/pnas.1215927110. 23401498 10.1073/pnas.1215927110 PMC3600440

[80] Aburto MR, Cryan JF. Gastrointestinal and brain barriers: unlocking gates of communication across the microbiota-gut-brain axis. Nat Rev Gastroenterol Hepatol. 2024;21(4):222–47. 10.1038/s41575-023-00890-0. 38355758 10.1038/s41575-023-00890-0

[81] Erny D, Hrabě de Angelis AL, Jaitin D, Wieghofer P, Staszewski O, David E, et al. Host microbiota constantly control maturation and function of microglia in the CNS. Nat Neurosci. 2015;18(7):965–77. 10.1038/nn.4030. 10.1038/nn.4030 PMC5528863 26030851

[82] Lyu Z, Hu Y, Guo Y, Liu D. Modulation of bone remodeling by the gut microbiota: a new therapy for osteoporosis. Bone Res. 2023;11(1):31. 10.1038/s41413-023-00264-x. 37296111 10.1038/s41413-023-00264-x PMC10256815

[83] Özçam M, Lynch SV. The gut-airway microbiome axis in health and respiratory diseases. Nat Rev Microbiol. 2024;22(8):492–506. 10.1038/s41579-024-01048-8. 38778224 10.1038/s41579-024-01048-8 PMC12051635

[84] Brown RL, Sequeira RP, Clarke TB. The microbiota protects against respiratory infection via GM-CSF signaling. Nat Commun. 2017;8(1):1512. 10.1038/s41467-017-01803-x. 29142211 10.1038/s41467-017-01803-x PMC5688119

[85] Bany Bakar R, Reimann F, Gribble FM. The intestine as an endocrine organ and the role of gut hormones in metabolic regulation. Nat Rev, Gastroenterol Hepatol. 2023;20(12):784–96. 10.1038/s41575-023-00830-y. 37626258 10.1038/s41575-023-00830-y

[86] Tolhurst G, Heffron H, Lam YS, Parker HE, Habib AM, Diakogiannaki E, et al. Short-chain fatty acids stimulate glucagon-like peptide-1 secretion via the G-protein-coupled receptor FFAR2. Diabetes. 2012;61(2):364–71. 10.2337/db11-1019. 22190648 10.2337/db11-1019 PMC3266401

[87] Hu S, Ding Q, Zhang W, Kang M, Ma J, Zhao L. Gut microbial beta-glucuronidase: a vital regulator in female estrogen metabolism. Gut Microbes. 2023;15(1):2236749. 10.1080/19490976.2023.2236749. 37559394 10.1080/19490976.2023.2236749 PMC10416750

[88] Ervin SM, Li H, Lim L, Roberts LR, Liang X, Mani S, et al. Gut microbial β-glucuronidases reactivate estrogens as components of the estrobolome that reactivate estrogens. J Biol Chem. 2019;294(49):18586–99. 10.1074/jbc.RA119.010950. 31636122 10.1074/jbc.RA119.010950 PMC6901331

[89] McCurry MD, D’Agostino GD, Walsh JT, Bisanz JE, Zalosnik I, Dong X, et al. Gut bacteria convert glucocorticoids into progestins in the presence of hydrogen gas. Cell. 2024;187(12):2952-2968.e13. 10.1016/j.cell.2024.05.005. 38795705 10.1016/j.cell.2024.05.005 PMC11179439

[90] Ohara TE, Hsiao EY. Microbiota-neuroepithelial signalling across the gut-brain axis. Nat Rev Microbiol. 2025;23(6):371–84. 10.1038/s41579-024-01136-9. 39743581 10.1038/s41579-024-01136-9

[91] Yano JM, Yu K, Donaldson GP, Shastri GG, Ann P, Ma L, et al. Indigenous bacteria from the gut microbiota regulate host serotonin biosynthesis. Cell. 2015;161(2):264–76. 10.1016/j.cell.2015.02.047. 25860609 10.1016/j.cell.2015.02.047 PMC4393509

[92] Bravo JA, Forsythe P, Chew MV, Escaravage E, Savignac HM, Dinan TG, et al. Ingestion of lactobacillus strain regulates emotional behavior and central GABA receptor expression in a mouse via the vagus nerve. Proc Natl Acad Sci U S A. 2011;108(38):16050–5. 10.1073/pnas.1102999108. 21876150 10.1073/pnas.1102999108 PMC3179073

[93] Carías Domínguez AM, de Jesús Rosa Salazar D, Stefanolo JP, Cruz Serrano MC, Casas IC, Zuluaga Peña JR. Intestinal dysbiosis: exploring definition, associated symptoms, and perspectives for a comprehensive understanding — a scoping review. Probiotics Antimicrob Proteins. 2025;17(1):440–9. 10.1007/s12602-024-10353-w. 10.1007/s12602-024-10353-w PMC11832579 39235661

[94] Shen Y, Fan N, Ma S xia, Cheng X, Yang X, Wang G. Gut microbiota dysbiosis: pathogenesis, diseases, prevention, and therapy. MedComm. 2025;6(5):e70168. 10.1002/mco2.70168. 10.1002/mco2.70168 PMC12006732 40255918

[95] Bidell MR, Hobbs ALV, Lodise TP. Gut microbiome health and dysbiosis: a clinical primer. Pharmacotherapy. 2022;42(11):849–57. 10.1002/phar.2731. 36168753 10.1002/phar.2731 PMC9827978

[96] Wei S, Bahl MI, Baunwall SMD, Hvas CL, Licht TR. Determining gut microbial dysbiosis: a review of applied indexes for assessment of intestinal microbiota imbalances. Appl Environ Microbiol. 2021;87(11):e00395-e421. 10.1128/AEM.00395-21. 33741632 10.1128/AEM.00395-21 PMC8208139

[97] Alagiakrishnan K, Morgadinho J, Halverson T. Approach to the diagnosis and management of dysbiosis. Front Nutr. 2024;11:1330903. 10.3389/fnut.2024.1330903. 38706561 10.3389/fnut.2024.1330903 PMC11069313

[98] H B. Problems with the concept of gut microbiota dysbiosis. Microb Biotechnol. 2020;13(2). 10.1111/1751-7915.13479. Cited 2026 Mar 1. 10.1111/1751-7915.13479 PMC7017827 31448542

[99] Safarchi A, Al-Qadami G, Tran CD, Conlon M. Understanding dysbiosis and resilience in the human gut microbiome: biomarkers, interventions, and challenges. Front Microbiol. 2025;16:1559521. 10.3389/fmicb.2025.1559521. 40104586 10.3389/fmicb.2025.1559521 PMC11913848

[100] Qiu P, Ishimoto T, Fu L, Zhang J, Zhang Z, Liu Y. The gut microbiota in inflammatory bowel disease. Front Cell Infect Microbiol. 2022;12:733992. 10.3389/fcimb.2022.733992. 35273921 10.3389/fcimb.2022.733992 PMC8902753

[101] Janney A, Powrie F, Mann EH. Host-microbiota maladaptation in colorectal cancer. Nature. 2020;585(7826):509–17. 10.1038/s41586-020-2729-3. 32968260 10.1038/s41586-020-2729-3

[102] Wang N, Fang JY. Fusobacterium nucleatum, a key pathogenic factor and microbial biomarker for colorectal cancer. Trends Microbiol. 2023;31(2):159–72. 10.1016/j.tim.2022.08.010. 36058786 10.1016/j.tim.2022.08.010

[103] Anthony WE, Burnham CAD, Dantas G, Kwon JH. The gut microbiome as a reservoir for antimicrobial resistance. J Infect Dis. 2021;223(12 Suppl 2):S209–13. 10.1093/infdis/jiaa497. 33326581 10.1093/infdis/jiaa497 PMC8206794

[104] Crits-Christoph A, Hallowell HA, Koutouvalis K, Suez J. Good microbes, bad genes? The dissemination of antimicrobial resistance in the human microbiome. Gut Microbes. 2022;14(1):2055944. 10.1080/19490976.2022.2055944. 35332832 10.1080/19490976.2022.2055944 PMC8959533

[105] Fishbein SRS, Mahmud B, Dantas G. Antibiotic perturbations to the gut microbiome. Nat Rev Microbiol. 2023;21(12):772–88. 10.1038/s41579-023-00933-y. 37491458 10.1038/s41579-023-00933-y PMC12087466

[106] Parizadeh M, Arrieta MC. The global human gut microbiome: genes, lifestyles, and diet. Trends Mol Med. 2023;29(10):789–801. 10.1016/j.molmed.2023.07.002. 37516570 10.1016/j.molmed.2023.07.002

[107] Perler BK, Friedman ES, Wu GD. The role of the gut microbiota in the relationship between diet and human health. Annu Rev Physiol. 2023;85:449–68. 10.1146/annurev-physiol-031522-092054. 36375468 10.1146/annurev-physiol-031522-092054

[108] Shin NR, Whon TW, Bae JW. Proteobacteria: microbial signature of dysbiosis in gut microbiota. Trends Biotechnol. 2015;33(9):496–503. 10.1016/j.tibtech.2015.06.011. 26210164 10.1016/j.tibtech.2015.06.011

[109] Martinez KB, Leone V, Chang EB. Western diets, gut dysbiosis, and metabolic diseases: are they linked? Gut Microbes. 2017;8(2):130–42. 10.1080/19490976.2016.1270811. 28059614 10.1080/19490976.2016.1270811 PMC5390820

[110] Vich Vila A, Collij V, Sanna S, Sinha T, Imhann F, Bourgonje AR, et al. Impact of commonly used drugs on the composition and metabolic function of the gut microbiota. Nat Commun. 2020;11(1):362. 10.1038/s41467-019-14177-z. 31953381 10.1038/s41467-019-14177-z PMC6969170

[111] Lindell AE, Zimmermann-Kogadeeva M, Patil KR. Multimodal interactions of drugs, natural compounds and pollutants with the gut microbiota. Nat Rev Microbiol. 2022;20(7):431–43. 10.1038/s41579-022-00681-5. 35102308 10.1038/s41579-022-00681-5 PMC7615390

[112] Szajewska H, Scott KP, de Meij T, Forslund-Startceva SK, Knight R, Koren O, et al. Antibiotic-perturbed microbiota and the role of probiotics. Nat Rev, Gastroenterol Hepatol. 2025;22(3):155–72. 10.1038/s41575-024-01023-x. 39663462 10.1038/s41575-024-01023-x

[113] Dethlefsen L, Relman DA. Incomplete recovery and individualized responses of the human distal gut microbiota to repeated antibiotic perturbation. Proc Natl Acad Sci U S A. 2011;108 Suppl 1(Suppl 1):4554–61. 10.1073/pnas.1000087107. 10.1073/pnas.1000087107 PMC3063582 20847294

[114] Ruiz VE, Battaglia T, Kurtz ZD, Bijnens L, Ou A, Engstrand I, et al. A single early-in-life macrolide course has lasting effects on murine microbial network topology and immunity. Nat Commun. 2017;8(1):518. 10.1038/s41467-017-00531-6. 28894149 10.1038/s41467-017-00531-6 PMC5593929

[115] Imhann F, Bonder MJ, Vich Vila A, Fu J, Mujagic Z, Vork L, et al. Proton pump inhibitors affect the gut microbiome. Gut. 2016;65(5):740–8. 10.1136/gutjnl-2015-310376. 26657899 10.1136/gutjnl-2015-310376 PMC4853569

[116] Wu H, Esteve E, Tremaroli V, Khan MT, Caesar R, Mannerås-Holm L, et al. Metformin alters the gut microbiome of individuals with treatment-naive type 2 diabetes, contributing to the therapeutic effects of the drug. Nat Med. 2017;23(7):850–8. 10.1038/nm.4345. 28530702 10.1038/nm.4345

[117] Maier L, Pruteanu M, Kuhn M, Zeller G, Telzerow A, Anderson EE, et al. Extensive impact of non-antibiotic drugs on human gut bacteria. Nature. 2018;555(7698):623–8. 10.1038/nature25979. 29555994 10.1038/nature25979 PMC6108420

[118] Zeng MY, Inohara N, Nuñez G. Mechanisms of inflammation-driven bacterial dysbiosis in the gut. Mucosal Immunol. 2017;10(1):18–26. 10.1038/mi.2016.75. 27554295 10.1038/mi.2016.75 PMC5788567

[119] Qin Y, Havulinna AS, Liu Y, Jousilahti P, Ritchie SC, Tokolyi A, et al. Combined effects of host genetics and diet on human gut microbiota and incident disease in a single population cohort. Nat Genet. 2022;54(2):134–42. 10.1038/s41588-021-00991-z. 35115689 10.1038/s41588-021-00991-z PMC9883041

[120] DeJong EN, Surette MG, Bowdish DME. The gut microbiota and unhealthy aging: disentangling cause from consequence. Cell Host Microbe. 2020;28(2):180–9. 10.1016/j.chom.2020.07.013. 32791111 10.1016/j.chom.2020.07.013

[121] Ghosh TS, Shanahan F, O’Toole PW. The gut microbiome as a modulator of healthy ageing. Nat Rev, Gastroenterol Hepatol. 2022;19(9):565–84. 10.1038/s41575-022-00605-x. 35468952 10.1038/s41575-022-00605-x PMC9035980

[122] Buttó LF, Haller D. Dysbiosis in intestinal inflammation: cause or consequence. Int J Med Microbiol. 2016;306(5):302–9. 10.1016/j.ijmm.2016.02.010. 27012594 10.1016/j.ijmm.2016.02.010

[123] Mouries J, Brescia P, Silvestri A, Spadoni I, Sorribas M, Wiest R, et al. Microbiota-driven gut vascular barrier disruption is a prerequisite for non-alcoholic steatohepatitis development. J Hepatol. 2019;71(6):1216–28. 10.1016/j.jhep.2019.08.005. 31419514 10.1016/j.jhep.2019.08.005 PMC6880766

[124] Di Vincenzo F, Del Gaudio A, Petito V, Lopetuso LR, Scaldaferri F. Gut microbiota, intestinal permeability, and systemic inflammation: a narrative review. Intern Emerg Med. 2024;19(2):275–93. 10.1007/s11739-023-03374-w. 37505311 10.1007/s11739-023-03374-w PMC10954893

[125] Lavelle A, Sokol H. Gut microbiota-derived metabolites as key actors in inflammatory bowel disease. Nat Rev, Gastroenterol Hepatol. 2020;17(4):223–37. 10.1038/s41575-019-0258-z. 32076145 10.1038/s41575-019-0258-z

[126] Koeth RA, Wang Z, Levison BS, Buffa JA, Org E, Sheehy BT, et al. Intestinal microbiota metabolism of L-carnitine, a nutrient in red meat, promotes atherosclerosis. Nat Med. 2013;19(5):576–85. 10.1038/nm.3145. 23563705 10.1038/nm.3145 PMC3650111

[127] Agus A, Clément K, Sokol H. Gut microbiota-derived metabolites as central regulators in metabolic disorders. Gut. 2021;70(6):1174–82. 10.1136/gutjnl-2020-323071. 33272977 10.1136/gutjnl-2020-323071 PMC8108286

[128] Krautkramer KA, Fan J, Bäckhed F. Gut microbial metabolites as multi-kingdom intermediates. Nat Rev Microbiol. 2021;19(2):77–94. 10.1038/s41579-020-0438-4. 32968241 10.1038/s41579-020-0438-4

[129] Yang W, Cong Y. Gut microbiota-derived metabolites in the regulation of host immune responses and immune-related inflammatory diseases. Cell Mol Immunol. 2021;18(4):866–77. 10.1038/s41423-021-00661-4. 33707689 10.1038/s41423-021-00661-4 PMC8115644

[130] Arifuzzaman M, Collins N, Guo CJ, Artis D. Nutritional regulation of microbiota-derived metabolites: implications for immunity and inflammation. Immunity. 2024;57(1):14–27. 10.1016/j.immuni.2023.12.009. 38198849 10.1016/j.immuni.2023.12.009 PMC10795735

[131] Margolis KG, Cryan JF, Mayer EA. The microbiota-gut-brain axis: from motility to mood. Gastroenterology. 2021;160(5):1486–501. 10.1053/j.gastro.2020.10.066. 33493503 10.1053/j.gastro.2020.10.066 PMC8634751

[132] Wong SH, Zhao L, Zhang X, Nakatsu G, Han J, Xu W, et al. Gavage of fecal samples from patients with colorectal cancer promotes intestinal carcinogenesis in germ-free and conventional mice. Gastroenterology. 2017;153(6):1621-1633.e6. 10.1053/j.gastro.2017.08.022. 28823860 10.1053/j.gastro.2017.08.022

[133] Li L, Li X, Zhong W, Yang M, Xu M, Sun Y, et al. Gut microbiota from colorectal cancer patients enhances the progression of intestinal adenoma in Apcmin/+ mice. EBioMedicine. 2019;48:301–15. 10.1016/j.ebiom.2019.09.021. 31594750 10.1016/j.ebiom.2019.09.021 PMC6838415

[134] Graillot V, Dormoy I, Dupuy J, Shay JW, Huc L, Mirey G, et al. Genotoxicity of Cytolethal Distending Toxin (CDT) on isogenic human colorectal cell lines: potential promoting effects for colorectal carcinogenesis. Front Cell Infect Microbiol. 2016;6:34. 10.3389/fcimb.2016.00034. 27047802 10.3389/fcimb.2016.00034 PMC4803749

[135] He Z, Yu J, Gong J, Wu J, Zong X, Luo Z, et al. Campylobacter jejuni-derived cytolethal distending toxin promotes colorectal cancer metastasis. Cell Host Microbe. 2024;32(12):2080-2091.e6. 10.1016/j.chom.2024.11.006. 39626677 10.1016/j.chom.2024.11.006

[136] He Z, Gharaibeh RZ, Newsome RC, Pope JL, Dougherty MW, Tomkovich S, et al. Campylobacter jejuni promotes colorectal tumorigenesis through the action of cytolethal distending toxin. Gut. 2019;68(2):289–300. 10.1136/gutjnl-2018-317200. 30377189 10.1136/gutjnl-2018-317200 PMC6352414

[137] Nougayrède JP, Homburg S, Taieb F, Boury M, Brzuszkiewicz E, Gottschalk G, et al. Escherichia coli induces DNA double-strand breaks in eukaryotic cells. Science. 2006;313(5788):848–51. 10.1126/science.1127059. 16902142 10.1126/science.1127059

[138] Dziubańska-Kusibab PJ, Berger H, Battistini F, Bouwman BAM, Iftekhar A, Katainen R, et al. Colibactin DNA-damage signature indicates mutational impact in colorectal cancer. Nat Med. 2020;26(7):1063–9. 10.1038/s41591-020-0908-2. 32483361 10.1038/s41591-020-0908-2

[139] Wilson MR, Jiang Y, Villalta PW, Stornetta A, Boudreau PD, Carrá A, et al. The human gut bacterial genotoxin colibactin alkylates DNA. Science. 2019;363(6428):eaar7785. 10.1126/science.aar7785. 10.1126/science.aar7785 PMC6407708 30765538

[140] Xue M, Kim CS, Healy AR, Wernke KM, Wang Z, Frischling MC, et al. Structure elucidation of colibactin and its DNA cross-links. Science. 2019;365(6457):eaax2685. 10.1126/science.aax2685. 10.1126/science.aax2685 PMC6820679 31395743

[141] Iftekhar A, Berger H, Bouznad N, Heuberger J, Boccellato F, Dobrindt U, et al. Genomic aberrations after short-term exposure to colibactin-producing E. coli transform primary colon epithelial cells. Nat Commun. 2021;12(1):1003. 10.1038/s41467-021-21162-y. 10.1038/s41467-021-21162-y PMC7881031 33579932

[142] Lucas C, Salesse L, Hoang MHT, Bonnet M, Sauvanet P, Larabi A, et al. Autophagy of intestinal epithelial cells inhibits colorectal carcinogenesis induced by colibactin-producing escherichia coli in ApcMin/+ Mice. Gastroenterology. 2020;158(5):1373–88. 10.1053/j.gastro.2019.12.026. 31917256 10.1053/j.gastro.2019.12.026

[143] Dalmasso G, Cougnoux A, Faïs T, Bonnin V, Mottet-Auselo B, Nguyen HT, et al. Colibactin-producing Escherichia coli enhance resistance to chemotherapeutic drugs by promoting epithelial to mesenchymal transition and cancer stem cell emergence. Gut Microbes. 2024;16(1):2310215. 10.1080/19490976.2024.2310215. 38374654 10.1080/19490976.2024.2310215 PMC10880512

[144] Lopès A, Billard E, Casse AH, Villéger R, Veziant J, Roche G, et al. Colibactin-positive Escherichia coli induce a procarcinogenic immune environment leading to immunotherapy resistance in colorectal cancer. Int J Cancer. 2020;146(11):3147–59. 10.1002/ijc.32920. 32037530 10.1002/ijc.32920

[145] Cornish AJ, Gruber AJ, Kinnersley B, Chubb D, Frangou A, Caravagna G, et al. The genomic landscape of 2,023 colorectal cancers. Nature. 2024;633(8028):127–36. 10.1038/s41586-024-07747-9. 39112709 10.1038/s41586-024-07747-9 PMC11374690

[146] Dougherty MW, Valdés-Mas R, Wernke KM, Gharaibeh RZ, Yang Y, Brant JO, et al. The microbial genotoxin colibactin exacerbates mismatch repair mutations in colorectal tumors. Neoplasia. 2023;43:100918. 10.1016/j.neo.2023.100918. 37499275 10.1016/j.neo.2023.100918 PMC10413156

[147] Volpe MR, Velilla JA, Daniel-Ivad M, Yao JJ, Stornetta A, Villalta PW, et al. A small molecule inhibitor prevents gut bacterial genotoxin production. Nat Chem Biol. 2023;19(2):159–67. 10.1038/s41589-022-01147-8. 36253549 10.1038/s41589-022-01147-8 PMC9889270

[148] Jans M, Kolata M, Blancke G, D’Hondt A, Gräf C, Ciers M, et al. Colibactin-driven colon cancer requires adhesin-mediated epithelial binding. Nature. 2024;635(8038):472–80. 10.1038/s41586-024-08135-z. 39506107 10.1038/s41586-024-08135-z

[149] Cao Y, Oh J, Xue M, Huh WJ, Wang J, Gonzalez-Hernandez JA, et al. Commensal microbiota from patients with inflammatory bowel disease produce genotoxic metabolites. Science. 2022;378(6618):eabm3233. 10.1126/science.abm3233. 10.1126/science.abm3233 PMC9993714 36302024

[150] Goodwin AC, Destefano Shields CE, Wu S, Huso DL, Wu X, Murray-Stewart TR, et al. Polyamine catabolism contributes to enterotoxigenic Bacteroides fragilis-induced colon tumorigenesis. Proc Natl Acad Sci U S A. 2011;108(37):15354–9. 10.1073/pnas.1010203108. 21876161 10.1073/pnas.1010203108 PMC3174648

[151] Clay SL, Fonseca-Pereira D, Garrett WS. Colorectal cancer: the facts in the case of the microbiota. J Clin Invest. 2022;132(4):e155101. 10.1172/JCI155101. 35166235 10.1172/JCI155101 PMC8843708

[152] Wang X, Huycke MM. Extracellular superoxide production by Enterococcus faecalis promotes chromosomal instability in mammalian cells. Gastroenterology. 2007;132(2):551–61. 10.1053/j.gastro.2006.11.040. 17258726 10.1053/j.gastro.2006.11.040

[153] Rubinstein MR, Wang X, Liu W, Hao Y, Cai G, Han YW. Fusobacterium nucleatum promotes colorectal carcinogenesis by modulating E-cadherin/β-catenin signaling via its FadA adhesin. Cell Host Microbe. 2013;14(2):195–206. 10.1016/j.chom.2013.07.012. 23954158 10.1016/j.chom.2013.07.012 PMC3770529

[154] Rubinstein MR, Baik JE, Lagana SM, Han RP, Raab WJ, Sahoo D, et al. Fusobacterium nucleatum promotes colorectal cancer by inducing Wnt/β‐catenin modulator Annexin A1. EMBO Rep. 2019;20(4):e47638. 10.15252/embr.201847638. 10.15252/embr.201847638 PMC6446206 30833345

[155] Yan X, Qu X, Wang J, Lu L, Wu W, Mao J, et al. *Fusobacterium nucleatum* promotes the growth and metastasis of colorectal cancer by activating E-Cadherin/Krüppel-Like Factor 4/Integrin α5 signaling in a calcium-dependent manner. MedComm. 2020;6(3):e70137. 10.1002/mco2.70137. 10.1002/mco2.70137 PMC11892016 40066228

[156] Zhang L, Leng XX, Qi J, Wang N, Han JX, Tao ZH, et al. The adhesin RadD enhances Fusobacterium nucleatum tumour colonization and colorectal carcinogenesis. Nat Microbiol. 2024;9(9):2292–307. 10.1038/s41564-024-01784-w. 39169124 10.1038/s41564-024-01784-w

[157] Wang N, Zhang L, Leng XX, Xie YL, Kang ZR, Zhao LC, et al. Fusobacterium nucleatum induces chemoresistance in colorectal cancer by inhibiting pyroptosis via the Hippo pathway. Gut Microbes. 2024;16(1):2333790. 10.1080/19490976.2024.2333790. 38533566 10.1080/19490976.2024.2333790 PMC10978024

[158] Zhang Y, Zhang L, Zheng S, Li M, Xu C, Jia D, et al. Fusobacterium nucleatum promotes colorectal cancer cells adhesion to endothelial cells and facilitates extravasation and metastasis by inducing ALPK1/NF-κB/ICAM1 axis. Gut Microbes. 2022;14(1):2038852. 10.1080/19490976.2022.2038852. 35220887 10.1080/19490976.2022.2038852 PMC8890384

[159] Chang Y, Huang Z, Hou F, Liu Y, Wang L, Wang Z, et al. Parvimonas micra activates the Ras/ERK/c-Fos pathway by upregulating miR-218-5p to promote colorectal cancer progression. J Exp Clin Cancer Res. 2023;42(1):13. 10.1186/s13046-022-02572-2. 36627634 10.1186/s13046-022-02572-2 PMC9830783

[160] Long X, Wong CC, Tong L, Chu ESH, Ho Szeto C, Go MYY, et al. Peptostreptococcus anaerobius promotes colorectal carcinogenesis and modulates tumour immunity. Nat Microbiol. 2019;4(12):2319–30. 10.1038/s41564-019-0541-3. 31501538 10.1038/s41564-019-0541-3

[161] Dong Y, Meng F, Wang J, Wei J, Zhang K, Qin S, et al. Desulfovibrio vulgaris flagellin exacerbates colorectal cancer through activating LRRC19/TRAF6/TAK1 pathway. Gut Microbes. 2025;17(1):2446376. 10.1080/19490976.2024.2446376. 39718561 10.1080/19490976.2024.2446376 PMC12931710

[162] Lu Y, Cui A, Zhang X. Commensal microbiota-derived metabolite agmatine triggers inflammation to promote colorectal tumorigenesis. Gut Microbes. 2024;16(1):2348441. 10.1080/19490976.2024.2348441. 38706224 10.1080/19490976.2024.2348441 PMC11086030

[163] Deng J, Zhang J, Su M, Li J, Su Y, Zhong Q, et al. Fusobacterium mortiferum and its metabolite 5-aminovaleric acid promote the development of colorectal cancer in obese individuals through wnt/β-catenin pathway by DKK2. Gut Microbes. 2025;17(1):2502138. 10.1080/19490976.2025.2502138. 40340623 10.1080/19490976.2025.2502138 PMC12064068

[164] Chen D, Jin D, Huang S, Wu J, Xu M, Liu T, et al. Clostridium butyricum, a butyrate-producing probiotic, inhibits intestinal tumor development through modulating Wnt signaling and gut microbiota. Cancer Lett. 2020;469:456–67. 10.1016/j.canlet.2019.11.019. 31734354 10.1016/j.canlet.2019.11.019

[165] Zhang S, Peng L, Goswami S, Li Y, Dang H, Xing S, et al. Intestinal crypt microbiota modulates intestinal stem cell turnover and tumorigenesis via indole acetic acid. Nat Microbiol. 2025;10(3):765–83. 10.1038/s41564-025-01937-5. 39972061 10.1038/s41564-025-01937-5

[166] Hu M, Xu Y, Wang Y, Huang Z, Wang L, Zeng F, et al. Gut microbial-derived N-acetylmuramic acid alleviates colorectal cancer via the AKT1 pathway. Gut. 2025;gutjnl-2024-332891. 10.1136/gutjnl-2024-332891. 10.1136/gutjnl-2024-332891 40015949

[167] Schmitt M, Greten FR. The inflammatory pathogenesis of colorectal cancer. Nat Rev Immunol. 2021;21(10):653–67. 10.1038/s41577-021-00534-x. 33911231 10.1038/s41577-021-00534-x

[168] Kostic AD, Chun E, Robertson L, Glickman JN, Gallini CA, Michaud M, et al. Fusobacterium nucleatum potentiates intestinal tumorigenesis and modulates the tumor immune microenvironment. Cell Host Microbe. 2013;14(2):207–15. 10.1016/j.chom.2013.07.007. 23954159 10.1016/j.chom.2013.07.007 PMC3772512

[169] Wu S, Rhee KJ, Albesiano E, Rabizadeh S, Wu X, Yen HR, et al. A human colonic commensal promotes colon tumorigenesis via activation of T helper type 17 T cell responses. Nat Med. 2009;15(9):1016–22. 10.1038/nm.2015. 19701202 10.1038/nm.2015 PMC3034219

[170] Brennan CA, Clay SL, Lavoie SL, Bae S, Lang JK, Fonseca-Pereira D, et al. Fusobacterium nucleatum drives a pro-inflammatory intestinal microenvironment through metabolite receptor-dependent modulation of IL-17 expression. Gut Microbes. 2021;13(1):1987780. 10.1080/19490976.2021.1987780. 34781821 10.1080/19490976.2021.1987780 PMC8604392

[171] Geis AL, Fan H, Wu X, Wu S, Huso DL, Wolfe JL, et al. Regulatory T-cell response to enterotoxigenic bacteroides fragilis colonization triggers IL17-dependent colon carcinogenesis. Cancer Discov. 2015;5(10):1098–109. 10.1158/2159-8290.CD-15-0447. 26201900 10.1158/2159-8290.CD-15-0447 PMC4592451

[172] Hurtado CG, Wan F, Housseau F, Sears CL. Roles for interleukin 17 and adaptive immunity in pathogenesis of colorectal cancer. Gastroenterology. 2018;155(6):1706–15. 10.1053/j.gastro.2018.08.056. 30218667 10.1053/j.gastro.2018.08.056 PMC6441974

[173] Wang K, Kim MK, Di Caro G, Wong J, Shalapour S, Wan J, et al. Interleukin-17 receptor a signaling in transformed enterocytes promotes early colorectal tumorigenesis. Immunity. 2014;41(6):1052–63. 10.1016/j.immuni.2014.11.009. 25526314 10.1016/j.immuni.2014.11.009 PMC4272447

[174] Yang Y, Weng W, Peng J, Hong L, Yang L, Toiyama Y, et al. Fusobacterium nucleatum increases proliferation of colorectal cancer cells and tumor development in mice by activating TLR4 signaling to NFκB, upregulating expression of microRNA-21. Gastroenterology. 2017;152(4):851-866.e24. 10.1053/j.gastro.2016.11.018. 27876571 10.1053/j.gastro.2016.11.018 PMC5555435

[175] Abed J, Emgård JEM, Zamir G, Faroja M, Almogy G, Grenov A, et al. Fap2 mediates fusobacterium nucleatum colorectal adenocarcinoma enrichment by binding to tumor-expressed Gal-GalNAc. Cell Host Microbe. 2016;20(2):215–25. 10.1016/j.chom.2016.07.006. 27512904 10.1016/j.chom.2016.07.006 PMC5465824

[176] Casasanta MA, Yoo CC, Udayasuryan B, Sanders BE, Umaña A, Zhang Y, et al. Fusobacterium nucleatum host-cell binding and invasion induces IL-8 and CXCL1 secretion that drives colorectal cancer cell migration. Sci Signal. 2020;13(641):eaba9157. 10.1126/scisignal.aba9157. 10.1126/scisignal.aba9157 PMC7454160 32694172

[177] Martin-Gallausiaux C, Salesse L, Garcia-Weber D, Marinelli L, Beguet-Crespel F, Brochard V, et al. Fusobacterium nucleatum promotes inflammatory and anti-apoptotic responses in colorectal cancer cells via ADP-heptose release and ALPK1/TIFA axis activation. Gut Microbes. 2024;16(1):2295384. 10.1080/19490976.2023.2295384. 38126163 10.1080/19490976.2023.2295384 PMC10761154

[178] Chung L, Thiele Orberg E, Geis AL, Chan JL, Fu K, DeStefano Shields CE, et al. *Bacteroides fragilis* toxin coordinates a pro-carcinogenic inflammatory cascade via targeting of colonic epithelial cells. Cell Host Microbe. 2018;23(2):203-214.e5. 10.1016/j.chom.2018.01.007. 29398651 10.1016/j.chom.2018.01.007 PMC5954996

[179] Thiele Orberg E, Fan H, Tam AJ, Dejea CM, Destefano Shields CE, Wu S, et al. The myeloid immune signature of enterotoxigenic Bacteroides fragilis-induced murine colon tumorigenesis. Mucosal Immunol. 2017;10(2):421–33. 10.1038/mi.2016.53. 27301879 10.1038/mi.2016.53 PMC5159334

[180] Zea AH, Rodriguez PC, Atkins MB, Hernandez C, Signoretti S, Zabaleta J, et al. Arginase-producing myeloid suppressor cells in renal cell carcinoma patients: a mechanism of tumor evasion. Cancer Res. 2005;65(8):3044–8. 10.1158/0008-5472.CAN-04-4505. 15833831 10.1158/0008-5472.CAN-04-4505

[181] Huang B, Pan PY, Li Q, Sato AI, Levy DE, Bromberg J, et al. Gr-1+CD115+ immature myeloid suppressor cells mediate the development of tumor-induced T regulatory cells and T-cell anergy in tumor-bearing host. Cancer Res. 2006;66(2):1123–31. 10.1158/0008-5472.CAN-05-1299. 16424049 10.1158/0008-5472.CAN-05-1299

[182] Gur C, Ibrahim Y, Isaacson B, Yamin R, Abed J, Gamliel M, et al. Binding of the Fap2 protein of Fusobacterium nucleatum to human inhibitory receptor TIGIT protects tumors from immune cell attack. Immunity. 2015;42(2):344–55. 10.1016/j.immuni.2015.01.010. 25680274 10.1016/j.immuni.2015.01.010 PMC4361732

[183] Galaski J, Shhadeh A, Umaña A, Yoo CC, Arpinati L, Isaacson B, et al. Fusobacterium nucleatum CbpF mediates inhibition of T cell function through CEACAM1 activation. Front Cell Infect Microbiol. 2021;11:692544. 10.3389/fcimb.2021.692544. 34336716 10.3389/fcimb.2021.692544 PMC8319768

[184] Galaski J, Rishiq A, Liu M, Bsoul R, Bergson A, Lux R, et al. *Fusobacterium nucleatum* subsp. *nucleatum* RadD binds siglec-7 and inhibits NK cell-mediated cancer cell killing. iScience. 2024;27(6):110157. 10.1016/j.isci.2024.110157. 38952680 10.1016/j.isci.2024.110157 PMC11215305

[185] Xu C, Fan L, Lin Y, Shen W, Qi Y, Zhang Y, et al. Fusobacterium nucleatum promotes colorectal cancer metastasis through miR-1322/CCL20 axis and M2 polarization. Gut Microbes. 2021;13(1):1980347. 10.1080/19490976.2021.1980347. 34632963 10.1080/19490976.2021.1980347 PMC8510564

[186] Chen T, Li Q, Wu J, Wu Y, Peng W, Li H, et al. Fusobacterium nucleatum promotes M2 polarization of macrophages in the microenvironment of colorectal tumours via a TLR4-dependent mechanism. Cancer Immunol Immunother: CII. 2018;67(10):1635–46. 10.1007/s00262-018-2233-x. 30121899 10.1007/s00262-018-2233-x PMC11028377

[187] Park SY, Pylaeva E, Bhuria V, Gambardella AR, Schiavoni G, Mougiakakos D, et al. Harnessing myeloid cells in cancer. Mol Cancer. 2025;24(1):69. 10.1186/s12943-025-02249-2. 40050933 10.1186/s12943-025-02249-2 PMC11887392

[188] Hexun Z, Miyake T, Maekawa T, Mori H, Yasukawa D, Ohno M, et al. High abundance of Lachnospiraceae in the human gut microbiome is related to high immunoscores in advanced colorectal cancer. Cancer Immunol Immunother. 2023;72(2):315–26. 10.1007/s00262-022-03256-8. 35869338 10.1007/s00262-022-03256-8 PMC10991469

[189] Zhang X, Yu D, Wu D, Gao X, Shao F, Zhao M, et al. Tissue-resident Lachnospiraceae family bacteria protect against colorectal carcinogenesis by promoting tumor immune surveillance. Cell Host Microbe. 2023;31(3):418-432.e8. 10.1016/j.chom.2023.01.013. 36893736 10.1016/j.chom.2023.01.013

[190] Nan K, Zhong Z, Yue Y, Shen Y, Zhang H, Wang Z, et al. Fasting-mimicking diet-enriched bifidobacterium pseudolongum suppresses colorectal cancer by inducing memory CD8+ T cells. Gut. 2025;74(5):775–86. 10.1136/gutjnl-2024-333020. 39870395 10.1136/gutjnl-2024-333020

[191] Fong W, Li Q, Ji F, Liang W, Lau HCH, Kang X, et al. Lactobacillus gallinarum-derived metabolites boost anti-PD1 efficacy in colorectal cancer by inhibiting regulatory T cells through modulating IDO1/Kyn/AHR axis. Gut. 2023;72(12):2272–85. 10.1136/gutjnl-2023-329543. 37770127 10.1136/gutjnl-2023-329543 PMC10715476

[192] Guo J, Meng F, Hu R, Chen L, Chang J, Zhao K, et al. Inhibition of the NF-κB/HIF-1α signaling pathway in colorectal cancer by tyrosol: a gut microbiota-derived metabolite. J Immunother Cancer. 2024;12(9):e008831. 10.1136/jitc-2024-008831. 39343509 10.1136/jitc-2024-008831 PMC11440206

[193] Sun Y, Wang Q, Jiang Y, He J, Jia D, Luo M, et al. Lactobacillus intestinalis facilitates tumor-derived CCL5 to recruit dendritic cell and suppress colorectal tumorigenesis. Gut Microbes. 2025;17(1):2449111. 10.1080/19490976.2024.2449111. 39773173 10.1080/19490976.2024.2449111 PMC11730368

[194] Wang X, Fang Y, Liang W, Wong CC, Qin H, Gao Y, et al. Fusobacterium nucleatum facilitates anti-PD-1 therapy in microsatellite stable colorectal cancer. Cancer Cell. 2024;42(10):1729-1746.e8. 10.1016/j.ccell.2024.08.019. 39303724 10.1016/j.ccell.2024.08.019

[195] Sorrenti E, Governa V, Bressan D, Cremonesi E, Formaggio N, Latino I, et al. Intratumoral microbiota and host genotype cooperatively shape neutrophil cytotoxic functions in colorectal cancer. Cell Host Microbe. 2026;34(3):425-443.e11. 10.1016/j.chom.2026.02.006. 41819084 10.1016/j.chom.2026.02.006

[196] Louis P, Hold GL, Flint HJ. The gut microbiota, bacterial metabolites and colorectal cancer. Nat Rev Microbiol. 2014;12(10):661–72. 10.1038/nrmicro3344. 25198138 10.1038/nrmicro3344

[197] Liu Y, Lau HCH, Yu J. Microbial metabolites in colorectal tumorigenesis and cancer therapy. Gut Microbes. 2023;15(1):2203968. 10.1080/19490976.2023.2203968. 37095682 10.1080/19490976.2023.2203968 PMC10132243

[198] Flint HJ, Scott KP, Duncan SH, Louis P, Forano E. Microbial degradation of complex carbohydrates in the gut. Gut Microbes. 2012;3(4):289–306. 10.4161/gmic.19897. 22572875 10.4161/gmic.19897 PMC3463488

[199] Hou H, Chen D, Zhang K, Zhang W, Liu T, Wang S, et al. Gut microbiota-derived short-chain fatty acids and colorectal cancer: ready for clinical translation? Cancer Lett. 2022;526:225–35. 10.1016/j.canlet.2021.11.027. 34843863 10.1016/j.canlet.2021.11.027

[200] Lavoie S, Chun E, Bae S, Brennan CA, Gallini Comeau CA, Lang JK, et al. Expression of free fatty acid receptor 2 by dendritic cells prevents their expression of interleukin 27 and is required for maintenance of mucosal barrier and immune response against colorectal tumors in mice. Gastroenterology. 2020;158(5):1359-1372.e9. 10.1053/j.gastro.2019.12.027. 31917258 10.1053/j.gastro.2019.12.027 PMC7291292

[201] Singh N, Gurav A, Sivaprakasam S, Brady E, Padia R, Shi H, et al. Activation of Gpr109a, receptor for niacin and the commensal metabolite butyrate, suppresses colonic inflammation and carcinogenesis. Immunity. 2014;40(1):128–39. 10.1016/j.immuni.2013.12.007. 24412617 10.1016/j.immuni.2013.12.007 PMC4305274

[202] Park J, Kim M, Kang SG, Jannasch AH, Cooper B, Patterson J, et al. Short-chain fatty acids induce both effector and regulatory T cells by suppression of histone deacetylases and regulation of the mTOR-S6K pathway. Mucosal Immunol. 2015;8(1):80–93. 10.1038/mi.2014.44. 24917457 10.1038/mi.2014.44 PMC4263689

[203] Yang Y, Misra BB, Liang L, Bi D, Weng W, Wu W, et al. Integrated microbiome and metabolome analysis reveals a novel interplay between commensal bacteria and metabolites in colorectal cancer. Theranostics. 2019;9(14):4101–14. 10.7150/thno.35186. 31281534 10.7150/thno.35186 PMC6592169

[204] Alvandi E, Wong WKM, Joglekar MV, Spring KJ, Hardikar AA. Short-chain fatty acid concentrations in the incidence and risk-stratification of colorectal cancer: a systematic review and meta-analysis. BMC Med. 2022;20(1):323. 10.1186/s12916-022-02529-4. 36184594 10.1186/s12916-022-02529-4 PMC9528142

[205] Kang X, Liu C, Ding Y, Ni Y, Ji F, Lau HCH, et al. *Roseburia intestinalis* generated butyrate boosts anti-PD-1 efficacy in colorectal cancer by activating cytotoxic CD8+ T cells. Gut. 2023;72(11):2112–22. 10.1136/gutjnl-2023-330291. 37491158 10.1136/gutjnl-2023-330291 PMC10579466

[206] He Y, Ling Y, Zhang Z, Mertens RT, Cao Q, Xu X, et al. Butyrate reverses ferroptosis resistance in colorectal cancer by inducing c-Fos-dependent xCT suppression. Redox Biol. 2023;65:102822. 10.1016/j.redox.2023.102822. 37494767 10.1016/j.redox.2023.102822 PMC10388208

[207] Okumura S, Konishi Y, Narukawa M, Sugiura Y, Yoshimoto S, Arai Y, et al. Gut bacteria identified in colorectal cancer patients promote tumourigenesis via butyrate secretion. Nat Commun. 2021;12(1):5674. 10.1038/s41467-021-25965-x. 34584098 10.1038/s41467-021-25965-x PMC8479117

[208] Bultman SJ, Jobin C. Microbial-derived butyrate: an oncometabolite or tumor-suppressive metabolite? Cell Host Microbe. 2014;16(2):143–5. 10.1016/j.chom.2014.07.011. 25121740 10.1016/j.chom.2014.07.011 PMC4179296

[209] Donohoe DR, Collins LB, Wali A, Bigler R, Sun W, Bultman SJ. The Warburg effect dictates the mechanism of butyrate-mediated histone acetylation and cell proliferation. Mol Cell. 2012;48(4):612–26. 10.1016/j.molcel.2012.08.033. 23063526 10.1016/j.molcel.2012.08.033 PMC3513569

[210] Jia B, Park D, Hahn Y, Jeon CO. Metagenomic analysis of the human microbiome reveals the association between the abundance of gut bile salt hydrolases and host health. Gut Microbes. 2020;11(5):1300–13. 10.1080/19490976.2020.1748261. 32329665 10.1080/19490976.2020.1748261 PMC7524343

[211] Ridlon JM, Hylemon PB. Identification and characterization of two bile acid coenzyme A transferases from Clostridium scindens, a bile acid 7α-dehydroxylating intestinal bacterium. J Lipid Res. 2012;53(1):66–76. 10.1194/jlr.M020313. 22021638 10.1194/jlr.M020313 PMC3243482

[212] Ajouz H, Mukherji D, Shamseddine A. Secondary bile acids: an underrecognized cause of colon cancer. World J Surg Oncol. 2014;12:164. 10.1186/1477-7819-12-164. 24884764 10.1186/1477-7819-12-164 PMC4041630

[213] Jia W, Xie G, Jia W. Bile acid–microbiota cross-talk in gastrointestinal inflammation and carcinogenesis. Nat Rev Gastroenterol Hepatol. 2018;15(2):111–28. 10.1038/nrgastro.2017.119. 29018272 10.1038/nrgastro.2017.119 PMC5899973

[214] Yao Y, Li X, Xu B, Luo L, Guo Q, Wang X, et al. Cholecystectomy promotes colon carcinogenesis by activating the Wnt signaling pathway by increasing the deoxycholic acid level. Cell Commun Signal. 2022;20(1):71. 10.1186/s12964-022-00890-8. 35614513 10.1186/s12964-022-00890-8 PMC9131663

[215] Bai X, Wei H, Liu W, Coker OO, Gou H, Liu C, et al. Cigarette smoke promotes colorectal cancer through modulation of gut microbiota and related metabolites. Gut. 2022;71(12):2439–50. 10.1136/gutjnl-2021-325021. 35387878 10.1136/gutjnl-2021-325021 PMC9664112

[216] Payne CM, Bernstein C, Dvorak K, Bernstein H. Hydrophobic bile acids, genomic instability, Darwinian selection, and colon carcinogenesis. Clin Exp Gastroenterol. 2008;1:19–47. 10.2147/ceg.s4343. 21677822 10.2147/ceg.s4343 PMC3108627

[217] Bernstein H, Bernstein C, Payne CM, Dvorak K. Bile acids as endogenous etiologic agents in gastrointestinal cancer. World J Gastroenterol. 2009;15(27):3329–40. 10.3748/wjg.15.3329. 19610133 10.3748/wjg.15.3329 PMC2712893

[218] Hang S, Paik D, Yao L, Kim E, Trinath J, Lu J, et al. Bile acid metabolites control TH17 and Treg cell differentiation. Nature. 2019;576(7785):143–8. 10.1038/s41586-019-1785-z. 31776512 10.1038/s41586-019-1785-z PMC6949019

[219] Cong J, Liu P, Han Z, Ying W, Li C, Yang Y, et al. Bile acids modified by the intestinal microbiota promote colorectal cancer growth by suppressing CD8+ T cell effector functions. Immunity. 2024;57(4):876-889.e11. 10.1016/j.immuni.2024.02.014. 38479384 10.1016/j.immuni.2024.02.014

[220] Cao H, Xu M, Dong W, Deng B, Wang S, Zhang Y, et al. Secondary bile acid-induced dysbiosis promotes intestinal carcinogenesis. Int J Cancer. 2017;140(11):2545–56. 10.1002/ijc.30643. 28187526 10.1002/ijc.30643

[221] Kim M, Vogtmann E, Ahlquist DA, Devens ME, Kisiel JB, Taylor WR, et al. Fecal metabolomic signatures in colorectal adenoma patients are associated with gut microbiota and early events of colorectal cancer pathogenesis. mBio. 2020;11(1):e03186–19. 10.1128/mBio.03186-19. 10.1128/mBio.03186-19 PMC7029137 32071266

[222] Ocvirk S, O’Keefe SJD. Dietary fat, bile acid metabolism and colorectal cancer. Semin Cancer Biol. 2021;73:347–55. 10.1016/j.semcancer.2020.10.003. 33069873 10.1016/j.semcancer.2020.10.003

[223] Gadaleta RM, Garcia-Irigoyen O, Moschetta A. Bile acids and colon cancer: is FXR the solution of the conundrum? Mol Aspects Med. 2017;56:66–74. 10.1016/j.mam.2017.04.002. 28400119 10.1016/j.mam.2017.04.002

[224] O’Keefe SJD, Li JV, Lahti L, Ou J, Carbonero F, Mohammed K, et al. Fat, fibre and cancer risk in African Americans and rural Africans. Nat Commun. 2015;6:6342. 10.1038/ncomms7342. 25919227 10.1038/ncomms7342 PMC4415091

[225] Wirbel J, Pyl PT, Kartal E, Zych K, Kashani A, Milanese A, et al. Meta-analysis of fecal metagenomes reveals global microbial signatures that are specific for colorectal cancer. Nat Med. 2019;25(4):679–89. 10.1038/s41591-019-0406-6. 30936547 10.1038/s41591-019-0406-6 PMC7984229

[226] Lee JY, Arai H, Nakamura Y, Fukiya S, Wada M, Yokota A. Contribution of the 7β-hydroxysteroid dehydrogenase from Ruminococcus gnavus N53 to ursodeoxycholic acid formation in the human colon. J Lipid Res. 2013;54(11):3062–9. 10.1194/jlr.M039834. 23729502 10.1194/jlr.M039834 PMC3793610

[227] Liu S, Zhou M, Huang X, Chen P, Li Q, Wang Y, et al. A mechanistic study of the feasibility of ursodeoxycholic acid in the treatment of colon adenocarcinoma. Drug Des Devel Ther. 2025;19:1839–52. 10.2147/DDDT.S500721. 40093647 10.2147/DDDT.S500721 PMC11910939

[228] Zhang H, Xu H, Zhang C, Tang Q, Bi F. Ursodeoxycholic acid suppresses the malignant progression of colorectal cancer through TGR5-YAP axis. Cell Death Discov. 2021;7(1):207. 10.1038/s41420-021-00589-8. 34365464 10.1038/s41420-021-00589-8 PMC8349355

[229] Sun F, Wang K, Dong X, Secaira-Morocho H, Hui A, Cai C, et al. The microbial bile acid metabolite 3-oxo-LCA inhibits colorectal cancer progression. Cancer Res. 2025;85(24):4937–57. 10.1158/0008-5472.CAN-24-3898. 40960502 10.1158/0008-5472.CAN-24-3898 PMC12871761

[230] Lin H, Yu Y, Zhu L, Lai N, Zhang L, Guo Y, et al. Implications of hydrogen sulfide in colorectal cancer: Mechanistic insights and diagnostic and therapeutic strategies. Redox Biol. 2023;59:102601. 10.1016/j.redox.2023.102601. 36630819 10.1016/j.redox.2023.102601 PMC9841368

[231] Nguyen LH, Ma W, Wang DD, Cao Y, Mallick H, Gerbaba TK, et al. Association between sulfur-metabolizing bacterial communities in stool and risk of distal colorectal cancer in men. Gastroenterology. 2020;158(5):1313–25. 10.1053/j.gastro.2019.12.029. 31972239 10.1053/j.gastro.2019.12.029 PMC7384232

[232] Han JX, Tao ZH, Wang JL, Zhang L, Yu CY, Kang ZR, et al. Microbiota-derived tryptophan catabolites mediate the chemopreventive effects of statins on colorectal cancer. Nat Microbiol. 2023;8(5):919–33. 10.1038/s41564-023-01363-5. 37069401 10.1038/s41564-023-01363-5

[233] Cui W, Guo M, Liu D, Xiao P, Yang C, Huang H, et al. Gut microbial metabolite facilitates colorectal cancer development via ferroptosis inhibition. Nat Cell Biol. 2024;26(1):124–37. 10.1038/s41556-023-01314-6. 38168770 10.1038/s41556-023-01314-6

[234] Zhou S, Wang K, Huang J, Xu Z, Yuan Q, Liu L, et al. Indole-3-lactic acid suppresses colorectal cancer via metabolic reprogramming. Gut Microbes. 2025;17(1):2508949. 10.1080/19490976.2025.2508949. 40409349 10.1080/19490976.2025.2508949 PMC12118437

[235] Coni S, Bordone R, Ivy DM, Yurtsever ZN, Di Magno L, D’Amico R, et al. Combined inhibition of polyamine metabolism and eIF5A hypusination suppresses colorectal cancer growth through a converging effect on MYC translation. Cancer Lett. 2023;559:216120. 10.1016/j.canlet.2023.216120. 36893894 10.1016/j.canlet.2023.216120

[236] Guo Y, Ye Q, Deng P, Cao Y, He D, Zhou Z, et al. Spermine synthase and MYC cooperate to maintain colorectal cancer cell survival by repressing Bim expression. Nat Commun. 2020;11(1):3243. 10.1038/s41467-020-17067-x. 32591507 10.1038/s41467-020-17067-x PMC7320137

[237] Shan Y, Lee M, Chang EB. The gut microbiome and inflammatory bowel diseases. Annu Rev Med. 2022;73:455–68. 10.1146/annurev-med-042320-021020. 34555295 10.1146/annurev-med-042320-021020 PMC10012812

[238] Franzosa EA, Sirota-Madi A, Avila-Pacheco J, Fornelos N, Haiser HJ, Reinker S, et al. Gut microbiome structure and metabolic activity in inflammatory bowel disease. Nat Microbiol. 2019;4(2):293–305. 10.1038/s41564-018-0306-4. 30531976 10.1038/s41564-018-0306-4 PMC6342642

[239] Peter Rimmer N, Zhang F, Scott G, Microbiome Data Provision Group, Hold GL, Gordon M, et al. The gut microbiome at the onset of inflammatory bowel disease: a systematic review and unified bioinformatic synthesis. Gastroenterology. 2026;170(3):539–56. 10.1053/j.gastro.2025.09.014. 10.1053/j.gastro.2025.09.014 41432650

[240] Sheikh IA, Bianchi-Smak J, Laubitz D, Schiro G, Midura-Kiela MT, Besselsen DG, et al. Transplant of microbiota from crohn’s disease patients to germ-free mice results in colitis. Gut Microbes. 2024;16(1):2333483. 10.1080/19490976.2024.2333483. 38532703 10.1080/19490976.2024.2333483 PMC10978031

[241] Sokol H, Pigneur B, Watterlot L, Lakhdari O, Bermúdez-Humarán LG, Gratadoux JJ, et al. Faecalibacterium prausnitzii is an anti-inflammatory commensal bacterium identified by gut microbiota analysis of crohn disease patients. Proc Natl Acad Sci U S A. 2008;105(43):16731–6. 10.1073/pnas.0804812105. 18936492 10.1073/pnas.0804812105 PMC2575488

[242] Machiels K, Joossens M, Sabino J, De Preter V, Arijs I, Eeckhaut V, et al. A decrease of the butyrate-producing species roseburia hominis and faecalibacterium prausnitzii defines dysbiosis in patients with ulcerative colitis. Gut. 2014;63(8):1275–83. 10.1136/gutjnl-2013-304833. 24021287 10.1136/gutjnl-2013-304833

[243] Deleu S, Machiels K, Raes J, Verbeke K, Vermeire S. Short chain fatty acids and its producing organisms: an overlooked therapy for IBD? EBioMedicine. 2021;66:103293. 10.1016/j.ebiom.2021.103293. 33813134 10.1016/j.ebiom.2021.103293 PMC8047503

[244] Shawki A, McCole DF. Mechanisms of intestinal epithelial barrier dysfunction by adherent-invasive escherichia coli. Cell Mol Gastroenterol Hepatol. 2017;3(1):41–50. 10.1016/j.jcmgh.2016.10.004. 28174756 10.1016/j.jcmgh.2016.10.004 PMC5247418

[245] Palmela C, Chevarin C, Xu Z, Torres J, Sevrin G, Hirten R, et al. Adherent-invasive escherichia coli in inflammatory bowel disease. Gut. 2018;67(3):574–87. 10.1136/gutjnl-2017-314903. 29141957 10.1136/gutjnl-2017-314903

[246] Steck N, Hoffmann M, Sava IG, Kim SC, Hahne H, Tonkonogy SL, et al. Enterococcus faecalis metalloprotease compromises epithelial barrier and contributes to intestinal inflammation. Gastroenterology. 2011;141(3):959–71. 10.1053/j.gastro.2011.05.035. 21699778 10.1053/j.gastro.2011.05.035

[247] Iliev ID, Ananthakrishnan AN, Guo CJ. Microbiota in inflammatory bowel disease: mechanisms of disease and therapeutic opportunities. Nat Rev Microbiol. 2025;23(8):509–24. 10.1038/s41579-025-01163-0. 40065181 10.1038/s41579-025-01163-0 PMC12289240

[248] Bruder E, Nedjar H, Quenech’Du N, Chevarin C, Vazeille E, Granotier M, et al. Persistence mechanisms of crohn’s disease-associated adherent invasive escherichia coli within macrophages. Gut Microbes. 2025;17(1):2587402. 10.1080/19490976.2025.2587402. 10.1080/19490976.2025.2587402 PMC12931692 41287568

[249] Henke MT, Kenny DJ, Cassilly CD, Vlamakis H, Xavier RJ, Clardy J. Ruminococcus gnavus, a member of the human gut microbiome associated with crohn’s disease, produces an inflammatory polysaccharide. Proc Natl Acad Sci U S A. 2019;116(26):12672–7. 10.1073/pnas.1904099116. 31182571 10.1073/pnas.1904099116 PMC6601261

[250] Xiang Z, Li X, Wang X, Deng B, He H, Xu M, et al. Fusobacterium nucleatum exacerbates colitis via STAT3 activation induced by acetyl-CoA accumulation. Gut Microbes. 2025;17(1):2489070. 10.1080/19490976.2025.2489070. 40212016 10.1080/19490976.2025.2489070 PMC12931693

[251] Atarashi K, Tanoue T, Shima T, Imaoka A, Kuwahara T, Momose Y, et al. Induction of colonic regulatory T cells by indigenous clostridium species. Science. 2011;331(6015):337–41. 10.1126/science.1198469. 21205640 10.1126/science.1198469 PMC3969237

[252] Round JL, Mazmanian SK. Inducible Foxp3+ regulatory T-cell development by a commensal bacterium of the intestinal microbiota. Proc Natl Acad Sci U S A. 2010;107(27):12204–9. 10.1073/pnas.0909122107. 20566854 10.1073/pnas.0909122107 PMC2901479

[253] Jia L, Jiang Y, Wu L, Fu J, Du J, Luo Z, et al. Porphyromonas gingivalis aggravates colitis via a gut microbiota-linoleic acid metabolism-Th17/treg cell balance axis. Nat Commun. 2024;15(1):1617. 10.1038/s41467-024-45473-y. 38388542 10.1038/s41467-024-45473-y PMC10883948

[254] Shen Z, Luo W, Tan B, Nie K, Deng M, Wu S, et al. Roseburia intestinalis stimulates TLR5-dependent intestinal immunity against crohn’s disease. EBioMedicine. 2022;85:104285. 10.1016/j.ebiom.2022.104285. 36182776 10.1016/j.ebiom.2022.104285 PMC9526137

[255] Gao H, Sun M, Li A, Gu Q, Kang D, Feng Z, et al. Microbiota-derived IPA alleviates intestinal mucosal inflammation through upregulating Th1/Th17 cell apoptosis in inflammatory bowel disease. Gut Microbes. 2025;17(1):2467235. 10.1080/19490976.2025.2467235. 39956891 10.1080/19490976.2025.2467235 PMC11834480

[256] Xu J, Li J, Guo X, Huang C, Peng Y, Xu H, et al. Secondary bile acids modified by odoribacter splanchnicus alleviate colitis by suppressing neutrophil extracellular trap formation. Adv Sci (Weinh). 2025;12(46):e09073. 10.1002/advs.202509073. 40990446 10.1002/advs.202509073 PMC12697870

[257] Luo W, Zhao M, Dwidar M, Gao Y, Xiang L, Wu X, et al. Microbial assimilatory sulfate reduction-mediated H2S: an overlooked role in crohn’s disease development. Microbiome. 2024;12(1):152. 10.1186/s40168-024-01873-2. 39152482 10.1186/s40168-024-01873-2 PMC11328384

[258] Ford AC, Sperber AD, Corsetti M, Camilleri M. Irritable bowel syndrome. Lancet. 2020;396(10263):1675–88. 10.1016/S0140-6736(20)31548-8. 33049223 10.1016/S0140-6736(20)31548-8

[259] Camilleri M, Boeckxstaens G. Irritable bowel syndrome: treatment based on pathophysiology and biomarkers. Gut. 2023;72(3):590–9. 10.1136/gutjnl-2022-328515. 36307180 10.1136/gutjnl-2022-328515 PMC9990119

[260] Pittayanon R, Lau JT, Yuan Y, Leontiadis GI, Tse F, Surette M, et al. Gut microbiota in patients with irritable bowel syndrome—a systematic review. Gastroenterology. 2019;157(1):97–108. 10.1053/j.gastro.2019.03.049. 30940523 10.1053/j.gastro.2019.03.049

[261] Altomare A, Di Rosa C, Imperia E, Emerenziani S, Cicala M, Guarino MPL. Diarrhea predominant-irritable bowel syndrome (IBS-D): effects of different nutritional patterns on intestinal dysbiosis and symptoms. Nutrients. 2021;13(5):1506. 10.3390/nu13051506. 33946961 10.3390/nu13051506 PMC8146452

[262] Di Rosa C, Altomare A, Terrigno V, Carbone F, Tack J, Cicala M, et al. Constipation-predominant irritable bowel syndrome (IBS-C): effects of different nutritional patterns on intestinal dysbiosis and symptoms. Nutrients. 2023;15(7):1647. 10.3390/nu15071647. 37049488 10.3390/nu15071647 PMC10096616

[263] Holtmann GJ, Ford AC, Talley NJ. Pathophysiology of irritable bowel syndrome. Lancet Gastroenterol Hepatol. 2016;1(2):133–46. 10.1016/S2468-1253(16)30023-1. 28404070 10.1016/S2468-1253(16)30023-1

[264] Mayer EA, Savidge T, Shulman RJ. Brain gut microbiome interactions and functional bowel disorders. Gastroenterology. 2014;146(6):1500–12. 10.1053/j.gastro.2014.02.037. 24583088 10.1053/j.gastro.2014.02.037 PMC4114504

[265] Bednarska O, Walter SA, Casado-Bedmar M, Ström M, Salvo-Romero E, Vicario M, et al. Vasoactive intestinal polypeptide and mast cells regulate increased passage of colonic bacteria in patients with irritable bowel syndrome. Gastroenterology. 2017;153(4):948-960.e3. 10.1053/j.gastro.2017.06.051. 28711627 10.1053/j.gastro.2017.06.051 PMC5623149

[266] Barbara G, Wang B, Stanghellini V, de Giorgio R, Cremon C, Di Nardo G, et al. Mast cell-dependent excitation of visceral-nociceptive sensory neurons in irritable bowel syndrome. Gastroenterology. 2007;132(1):26–37. 10.1053/j.gastro.2006.11.039. 17241857 10.1053/j.gastro.2006.11.039

[267] Mayer EA, Ryu HJ, Bhatt RR. The neurobiology of irritable bowel syndrome. Mol Psychiatry. 2023;28(4):1451–65. 10.1038/s41380-023-01972-w. 36732586 10.1038/s41380-023-01972-w PMC10208985

[268] Yuan Y, Wang X, Huang S, Wang H, Shen G. Low-level inflammation, immunity, and brain-gut axis in IBS: unraveling the complex relationships. Gut Microbes. 2023;15(2):2263209. 10.1080/19490976.2023.2263209. 37786296 10.1080/19490976.2023.2263209 PMC10549202

[269] Balemans D, Mondelaers SU, Cibert-Goton V, Stakenborg N, Aguilera-Lizarraga J, Dooley J, et al. Evidence for long-term sensitization of the bowel in patients with post-infectious-IBS. Sci Rep. 2017;7(1):13606. 10.1038/s41598-017-12618-7. 29051514 10.1038/s41598-017-12618-7 PMC5648751

[270] Xiao L, Liu Q, Luo M, Xiong L. Gut microbiota-derived metabolites in irritable bowel syndrome. Front Cell Infect Microbiol. 2021;11:729346. 10.3389/fcimb.2021.729346. 34631603 10.3389/fcimb.2021.729346 PMC8495119

[271] Gu Y, Li L, Yang M, Liu T, Song X, Qin X, et al. Bile acid-gut microbiota crosstalk in irritable bowel syndrome. Crit Rev Microbiol. 2023;49(3):350–69. 10.1080/1040841X.2022.2058353. 35389754 10.1080/1040841X.2022.2058353

[272] Zhao L, Yang W, Chen Y, Huang F, Lu L, Lin C, et al. A clostridia-rich microbiota enhances bile acid excretion in diarrhea-predominant irritable bowel syndrome. J Clin Invest. 2020;130(1):438–50. 10.1172/JCI130976. 31815740 10.1172/JCI130976 PMC6934182

[273] Ghoshal U, Shukla R, Srivastava D, Ghoshal UC. Irritable bowel syndrome, particularly the constipation-predominant form, involves an increase in methanobrevibacter smithii, which is associated with higher methane production. Gut Liver. 2016;10(6):932–8. 10.5009/gnl15588. 27458176 10.5009/gnl15588 PMC5087933

[274] Pimentel M, Lin HC, Enayati P, van den Burg B, Lee HR, Chen JH, et al. Methane, a gas produced by enteric bacteria, slows intestinal transit and augments small intestinal contractile activity. Am J Physiol Gastrointest Liver Physiol. 2006;290(6):G1089-1095. 10.1152/ajpgi.00574.2004. 16293652 10.1152/ajpgi.00574.2004

[275] Ringel-Kulka T, Choi CH, Temas D, Kim A, Maier DM, Scott K, et al. Altered colonic bacterial fermentation as a potential pathophysiological factor in irritable bowel syndrome. Am J Gastroenterol. 2015;110(9):1339–46. 10.1038/ajg.2015.220. 26303129 10.1038/ajg.2015.220 PMC4983766

[276] Hsu CL, Schnabl B. The gut-liver axis and gut microbiota in health and liver disease. Nat Rev Microbiol. 2023;21(11):719–33. 10.1038/s41579-023-00904-3. 37316582 10.1038/s41579-023-00904-3 PMC10794111

[277] Albillos A, de Gottardi A, Rescigno M. The gut-liver axis in liver disease: pathophysiological basis for therapy. J Hepatol. 2020;72(3):558–77. 10.1016/j.jhep.2019.10.003. 31622696 10.1016/j.jhep.2019.10.003

[278] Tilg H, Adolph TE, Trauner M. Gut-liver axis: Pathophysiological concepts and clinical implications. Cell Metab. 2022;34(11):1700–18. 10.1016/j.cmet.2022.09.017. 36208625 10.1016/j.cmet.2022.09.017

[279] Pabst O, Hornef MW, Schaap FG, Cerovic V, Clavel T, Bruns T. Gut-liver axis: barriers and functional circuits. Nat Rev, Gastroenterol Hepatol. 2023;20(7):447–61. 10.1038/s41575-023-00771-6. 37085614 10.1038/s41575-023-00771-6

[280] Wahlström A, Sayin SI, Marschall HU, Bäckhed F. Intestinal crosstalk between bile acids and microbiota and its impact on host metabolism. Cell Metab. 2016;24(1):41–50. 10.1016/j.cmet.2016.05.005. 27320064 10.1016/j.cmet.2016.05.005

[281] Ridlon JM, Gaskins HR. Another renaissance for bile acid gastrointestinal microbiology. Nat Rev, Gastroenterol Hepatol. 2024;21(5):348–64. 10.1038/s41575-024-00896-2. 38383804 10.1038/s41575-024-00896-2 PMC11558780

[282] Lau HCH, Zhang X, Yu J. Gut microbiome in metabolic dysfunction-associated steatotic liver disease and associated hepatocellular carcinoma. Nat Rev Gastroenterol Hepatol. 2025;22(9):619–38. 10.1038/s41575-025-01089-1. 40624229 10.1038/s41575-025-01089-1

[283] Hammerich L, Tacke F. Hepatic inflammatory responses in liver fibrosis. Nat Rev, Gastroenterol Hepatol. 2023;20(10):633–46. 10.1038/s41575-023-00807-x. 37400694 10.1038/s41575-023-00807-x

[284] Schnabl B, Damman CJ, Carr RM. Metabolic dysfunction-associated steatotic liver disease and the gut microbiome: pathogenic insights and therapeutic innovations. J Clin Invest. 2025;135(7):e186423. 10.1172/JCI186423. 40166938 10.1172/JCI186423 PMC11957707

[285] Fuchs CD, Simbrunner B, Baumgartner M, Campbell C, Reiberger T, Trauner M. Bile acid metabolism and signalling in liver disease. J Hepatol. 2025;82(1):134–53. 10.1016/j.jhep.2024.09.032. 39349254 10.1016/j.jhep.2024.09.032

[286] Kuang J, Wang J, Li Y, Li M, Zhao M, Ge K, et al. Hyodeoxycholic acid alleviates non-alcoholic fatty liver disease through modulating the gut-liver axis. Cell Metab. 2023;35(10):1752-1766.e8. 10.1016/j.cmet.2023.07.011. 37591244 10.1016/j.cmet.2023.07.011

[287] Samuel BS, Shaito A, Motoike T, Rey FE, Backhed F, Manchester JK, et al. Effects of the gut microbiota on host adiposity are modulated by the short-chain fatty-acid binding G protein-coupled receptor, Gpr41. Proc Natl Acad Sci U S A. 2008;105(43):16767–72. 10.1073/pnas.0808567105. 18931303 10.1073/pnas.0808567105 PMC2569967

[288] Kim MH, Kang SG, Park JH, Yanagisawa M, Kim CH. Short-chain fatty acids activate GPR41 and GPR43 on intestinal epithelial cells to promote inflammatory responses in mice. Gastroenterology. 2013;145(2):396–406.e1–10. 10.1053/j.gastro.2013.04.056. 10.1053/j.gastro.2013.04.056 23665276

[289] Agus A, Planchais J, Sokol H. Gut microbiota regulation of tryptophan metabolism in health and disease. Cell Host Microbe. 2018;23(6):716–24. 10.1016/j.chom.2018.05.003. 29902437 10.1016/j.chom.2018.05.003

[290] Teunis C, Nieuwdorp M, Hanssen N. Interactions between tryptophan metabolism, the gut microbiome and the immune system as potential drivers of non-alcoholic fatty liver disease (NAFLD) and metabolic diseases. Metabolites. 2022;12(6):514. 10.3390/metabo12060514. 35736447 10.3390/metabo12060514 PMC9227929

[291] Yuan J, Chen C, Cui J, Lu J, Yan C, Wei X, et al. Fatty liver disease caused by high-alcohol-producing klebsiella pneumoniae. Cell Metab. 2019;30(4):675-688.e7. 10.1016/j.cmet.2019.08.018. 31543403 10.1016/j.cmet.2019.08.018

[292] Meijnikman AS, Davids M, Herrema H, Aydin O, Tremaroli V, Rios-Morales M, et al. Microbiome-derived ethanol in nonalcoholic fatty liver disease. Nat Med. 2022;28(10):2100–6. 10.1038/s41591-022-02016-6. 36216942 10.1038/s41591-022-02016-6

[293] Nian F, Chen Y, Xia Q, Zhu C, Wu L, Lu X. Gut microbiota metabolite trimethylamine N-oxide promoted NAFLD progression by exacerbating intestinal barrier disruption and intrahepatic cellular imbalance. Int Immunopharmacol. 2024;142(Pt B):113173. 10.1016/j.intimp.2024.113173. 39298816 10.1016/j.intimp.2024.113173

[294] Jang JW, Capaldi E, Smith T, Verma P, Varga J, Ho KJ. Trimethylamine N-oxide: a meta-organismal axis linking the gut and fibrosis. Mol Med. 2024;30(1):128. 10.1186/s10020-024-00895-8. 39180015 10.1186/s10020-024-00895-8 PMC11344357

[295] Swanson KV, Deng M, Ting JPY. The NLRP3 inflammasome: molecular activation and regulation to therapeutics. Nat Rev Immunol. 2019;19(8):477–89. 10.1038/s41577-019-0165-0. 31036962 10.1038/s41577-019-0165-0 PMC7807242

[296] Csak T, Ganz M, Pespisa J, Kodys K, Dolganiuc A, Szabo G. Fatty acid and endotoxin activate inflammasomes in mouse hepatocytes that release danger signals to stimulate immune cells. Hepatology. 2011;54(1):133–44. 10.1002/hep.24341. 21488066 10.1002/hep.24341 PMC4158408

[297] Han YH, Onufer EJ, Huang LH, Sprung RW, Davidson WS, Czepielewski RS, et al. Enterically derived high-density lipoprotein restrains liver injury through the portal vein. Science. 2021;373(6553):eabe6729. 10.1126/science.abe6729. 10.1126/science.abe6729 PMC8478306 34437091

[298] Kim YS, Hurley EH, Park Y, Ko S. Primary sclerosing cholangitis (PSC) and inflammatory bowel disease (IBD): a condition exemplifying the crosstalk of the gut-liver axis. Exp Mol Med. 2023;55(7):1380–7. 10.1038/s12276-023-01042-9. 37464092 10.1038/s12276-023-01042-9 PMC10394020

[299] Hov JR, Karlsen TH. The microbiota and the gut-liver axis in primary sclerosing cholangitis. Nat Rev, Gastroenterol Hepatol. 2023;20(3):135–54. 10.1038/s41575-022-00690-y. 36352157 10.1038/s41575-022-00690-y

[300] Eksteen B, Grant AJ, Miles A, Curbishley SM, Lalor PF, Hübscher SG, et al. Hepatic endothelial CCL25 mediates the recruitment of CCR9+ gut-homing lymphocytes to the liver in primary sclerosing cholangitis. J Exp Med. 2004;200(11):1511–7. 10.1084/jem.20041035. 15557349 10.1084/jem.20041035 PMC2211943

[301] de Krijger M, Visseren T, Wildenberg ME, Hooijer GKJ, Verstegen MMA, van der Laan LJW, et al. Characterization of gut-homing molecules in non-endstage livers of patients with primary sclerosing cholangitis and inflammatory bowel disease. J Transl Autoimmun. 2020;3:100054. 10.1016/j.jtauto.2020.100054. 32743534 10.1016/j.jtauto.2020.100054 PMC7388383

[302] Trivedi PJ, Hirschfield GM, Adams DH, Vierling JM. Immunopathogenesis of primary biliary cholangitis, primary sclerosing cholangitis and autoimmune hepatitis: themes and concepts. Gastroenterology. 2024;166(6):995–1019. 10.1053/j.gastro.2024.01.049. 38342195 10.1053/j.gastro.2024.01.049

[303] Greenman R, Segal-Salto M, Barashi N, Hay O, Katav A, Levi O, et al. CCL24 regulates biliary inflammation and fibrosis in primary sclerosing cholangitis. JCI insight. 2023;8(12):e162270. 10.1172/jci.insight.162270. 37345655 10.1172/jci.insight.162270 PMC10371243

[304] Henriksen EKK, Jørgensen KK, Kaveh F, Holm K, Hamm D, Olweus J, et al. Gut and liver T-cells of common clonal origin in primary sclerosing cholangitis-inflammatory bowel disease. J Hepatol. 2017;66(1):116–22. 10.1016/j.jhep.2016.09.002. 27647428 10.1016/j.jhep.2016.09.002

[305] Trebicka J, Bork P, Krag A, Arumugam M. Utilizing the gut microbiome in decompensated cirrhosis and acute-on-chronic liver failure. Nat Rev, Gastroenterol Hepatol. 2021;18(3):167–80. 10.1038/s41575-020-00376-3. 33257833 10.1038/s41575-020-00376-3

[306] Bajaj JS, Sikaroodi M, Shamsaddini A, Henseler Z, Santiago-Rodriguez T, Acharya C, et al. Interaction of bacterial metagenome and virome in patients with cirrhosis and hepatic encephalopathy. Gut. 2021;70(6):1162–73. 10.1136/gutjnl-2020-322470. 32998876 10.1136/gutjnl-2020-322470

[307] Qin N, Yang F, Li A, Prifti E, Chen Y, Shao L, et al. Alterations of the human gut microbiome in liver cirrhosis. Nature. 2014;513(7516):59–64. 10.1038/nature13568. 25079328 10.1038/nature13568

[308] Albillos A, Martin-Mateos R, Van der Merwe S, Wiest R, Jalan R, Álvarez-Mon M. Cirrhosis-associated immune dysfunction. Nat Rev, Gastroenterol Hepatol. 2022;19(2):112–34. 10.1038/s41575-021-00520-7. 34703031 10.1038/s41575-021-00520-7

[309] Trebicka J, Macnaughtan J, Schnabl B, Shawcross DL, Bajaj JS. The microbiota in cirrhosis and its role in hepatic decompensation. J Hepatol. 2021;75 Suppl 1(Suppl 1):S67–81. 10.1016/j.jhep.2020.11.013. 10.1016/j.jhep.2020.11.013 PMC8973011 34039493

[310] Sharma SP, Gupta H, Kwon GH, Lee SY, Song SH, Kim JS, et al. Gut microbiome and metabolome signatures in liver cirrhosis-related complications. Clin Mol Hepatol. 2024;30(4):845–62. 10.3350/cmh.2024.0349. 39048520 10.3350/cmh.2024.0349 PMC11540350

[311] He X, Hu M, Xu Y, Xia F, Tan Y, Wang Y, et al. The gut-brain axis underlying hepatic encephalopathy in liver cirrhosis. Nat Med. 2025;31(2):627–38. 10.1038/s41591-024-03405-9. 39779925 10.1038/s41591-024-03405-9

[312] Loh JS, Mak WQ, Tan LKS, Ng CX, Chan HH, Yeow SH, et al. Microbiota-gut-brain axis and its therapeutic applications in neurodegenerative diseases. Signal Transduct Target Ther. 2024;9(1):37. 10.1038/s41392-024-01743-1. 38360862 10.1038/s41392-024-01743-1 PMC10869798

[313] O’Riordan KJ, Moloney GM, Keane L, Clarke G, Cryan JF. The gut microbiota-immune-brain axis: therapeutic implications. Cell Rep Med. 2025;6(3):101982. 10.1016/j.xcrm.2025.101982. 40054458 10.1016/j.xcrm.2025.101982 PMC11970326

[314] Grundeken E, El Aidy S. Enteroendocrine cells: the gatekeepers of microbiome-gut-brain communication. npj Biofilms Microbiomes. 2025;11(1):179. 10.1038/s41522-025-00810-x. 10.1038/s41522-025-00810-x PMC12402511 40890205

[315] Wang Q, Yang Q, Liu X. The microbiota-gut-brain axis and neurodevelopmental disorders. Protein Cell. 2023;14(10):762–75. 10.1093/procel/pwad026. 37166201 10.1093/procel/pwad026 PMC10599644

[316] You M, Chen N, Yang Y, Cheng L, He H, Cai Y, et al. The gut microbiota-brain axis in neurological disorders. MedComm. 2024;5(8):e656. 10.1002/mco2.656. 39036341 10.1002/mco2.656 PMC11260174

[317] Park JC, Sim MA, Lee C, Park HE, Lee J, Choi SY, et al. Gut microbiota and brain-resident CD4+ T cells shape behavioral outcomes in autism spectrum disorder. Nat Commun. 2025;16(1):6422. 10.1038/s41467-025-61544-0. 40645945 10.1038/s41467-025-61544-0 PMC12254219

[318] Flynn CK, Adams JB, Krajmalnik-Brown R, Khoruts A, Sadowsky MJ, Nirmalkar K, et al. Review of elevated para-cresol in autism and possible impact on symptoms. Int J Mol Sci. 2025;26(4):1513. 10.3390/ijms26041513. 40003979 10.3390/ijms26041513 PMC11855632

[319] Giniatullin R, Cherubini E. Gabaergic signalling in autism spectrum disorders (ASD): role of glial cells and therapeutic perspectives. Brain Behav Immun. 2025;129:681–9. 10.1016/j.bbi.2025.07.003. 40623670 10.1016/j.bbi.2025.07.003

[320] Qu S, Yu Z, Zhou Y, Wang S, Jia M, Chen T, et al. Gut microbiota modulates neurotransmitter and gut-brain signaling. Microbiol Res. 2024;287:127858. 10.1016/j.micres.2024.127858. 39106786 10.1016/j.micres.2024.127858

[321] Ahrens AP, Hyötyläinen T, Petrone JR, Igelström K, George CD, Garrett TJ, et al. Infant microbes and metabolites point to childhood neurodevelopmental disorders. Cell. 2024;187(8):1853-1873.e15. 10.1016/j.cell.2024.02.035. 38574728 10.1016/j.cell.2024.02.035

[322] Tan AH, Lim SY, Lang AE. The microbiome–gut–brain axis in parkinson disease — from basic research to the clinic. Nat Rev Neurol. 2022;18(8):476–95. 10.1038/s41582-022-00681-2. 35750883 10.1038/s41582-022-00681-2

[323] Zhang X, Tang B, Guo J. Parkinson’s disease and gut microbiota: from clinical to mechanistic and therapeutic studies. Transl Neurodegener. 2023;12:59. 10.1186/s40035-023-00392-8. 38098067 10.1186/s40035-023-00392-8 PMC10722742

[324] Fasano A, Visanji NP, Liu LWC, Lang AE, Pfeiffer RF. Gastrointestinal dysfunction in parkinson’s disease. Lancet, Neurol. 2015;14(6):625–39. 10.1016/S1474-4422(15)00007-1. 25987282 10.1016/S1474-4422(15)00007-1

[325] Hirayama M, Nishiwaki H, Hamaguchi T, Ohno K. Gastrointestinal disorders in parkinson’s disease and other lewy body diseases. npj Parkinson’s Dis. 2023;9(1):71. 10.1038/s41531-023-00511-2. 10.1038/s41531-023-00511-2 PMC10160728 37147392

[326] Braak H, Del Tredici K, Rüb U, de Vos RAI, Jansen Steur ENH, Braak E. Staging of brain pathology related to sporadic parkinson’s disease. Neurobiol Aging. 2003;24(2):197–211. 10.1016/s0197-4580(02)00065-9. 12498954 10.1016/s0197-4580(02)00065-9

[327] Munoz-Pinto MF, Candeias E, Melo-Marques I, Esteves AR, Maranha A, Magalhães JD, et al. Gut-first parkinson’s disease is encoded by gut dysbiome. Mol Neurodegener. 2024;19(1):78. 10.1186/s13024-024-00766-0. 39449004 10.1186/s13024-024-00766-0 PMC11515425

[328] Chen SG, Stribinskis V, Rane MJ, Demuth DR, Gozal E, Roberts AM, et al. Exposure to the functional bacterial amyloid protein curli enhances alpha-synuclein aggregation in aged fischer 344 rats and caenorhabditis elegans. Sci Rep. 2016;6:34477. 10.1038/srep34477. 27708338 10.1038/srep34477 PMC5052651

[329] Kim S, Kwon SH, Kam TI, Panicker N, Karuppagounder SS, Lee S, et al. Transneuronal propagation of pathologic α-synuclein from the gut to the brain models parkinson’s disease. Neuron. 2019;103(4):627-641.e7. 10.1016/j.neuron.2019.05.035. 31255487 10.1016/j.neuron.2019.05.035 PMC6706297

[330] Kalyanaraman B, Cheng G, Hardy M. Gut microbiome, short-chain fatty acids, alpha-synuclein, neuroinflammation, and ROS/RNS: relevance to parkinson’s disease and therapeutic implications. Redox Biol. 2024;71:103092. 10.1016/j.redox.2024.103092. 38377788 10.1016/j.redox.2024.103092 PMC10891329

[331] Liu L, Wang H, Chen X, Zhang Y, Zhang H, Xie P. Gut microbiota and its metabolites in depression: from pathogenesis to treatment. eBioMedicine. 2023;90:104527. 10.1016/j.ebiom.2023.104527. 10.1016/j.ebiom.2023.104527 PMC10051028 36963238

[332] Akif A, Islam MR. The microbiota-gut-brain axis in the pathophysiology of major depressive disorder: a mechanistic review. Compr Physiol. 2026;16(1):e70100. 10.1002/cph4.70100. 41534880 10.1002/cph4.70100

[333] O’Connor JC, Lawson MA, André C, Moreau M, Lestage J, Castanon N, et al. Lipopolysaccharide-induced depressive-like behavior is mediated by indoleamine 2,3-dioxygenase activation in mice. Mol Psychiatry. 2009;14(5):511–22. 10.1038/sj.mp.4002148. 18195714 10.1038/sj.mp.4002148 PMC2683474

[334] Deng Y, Zhou M, Wang J, Yao J, Yu J, Liu W, et al. Involvement of the microbiota-gut-brain axis in chronic restraint stress: disturbances of the kynurenine metabolic pathway in both the gut and brain. Gut Microbes. 2021;13(1):1–16. 10.1080/19490976.2020.1869501. 33535879 10.1080/19490976.2020.1869501 PMC7872056

[335] Gao K, Mu CL, Farzi A, Zhu WY. Tryptophan metabolism: a link between the gut microbiota and brain. Adv Nutr. 2020;11(3):709–23. 10.1093/advances/nmz127. 31825083 10.1093/advances/nmz127 PMC7231603

[336] Zhou M, Fan Y, Xu L, Yu Z, Wang S, Xu H, et al. Microbiome and tryptophan metabolomics analysis in adolescent depression: roles of the gut microbiota in the regulation of tryptophan-derived neurotransmitters and behaviors in human and mice. Microbiome. 2023;11(1):145. 10.1186/s40168-023-01589-9. 37386523 10.1186/s40168-023-01589-9 PMC10311725

[337] Delgadillo DR, Borelli JL, Mayer EA, Labus JS, Cross MP, Pressman SD. Biological, environmental, and psychological stress and the human gut microbiome in healthy adults. Sci Rep. 2025;15(1):362. 10.1038/s41598-024-77473-9. 39747287 10.1038/s41598-024-77473-9 PMC11695967

[338] Vodička M, Ergang P, Hrnčíř T, Mikulecká A, Kvapilová P, Vagnerová K, et al. Microbiota affects the expression of genes involved in HPA axis regulation and local metabolism of glucocorticoids in chronic psychosocial stress. Brain Behav Immun. 2018;73:615–24. 10.1016/j.bbi.2018.07.007. 29990567 10.1016/j.bbi.2018.07.007

[339] Allen JM, Mackos AR, Jaggers RM, Brewster PC, Webb M, Lin CH, et al. Psychological stress disrupts intestinal epithelial cell function and mucosal integrity through microbe and host-directed processes. Gut Microbes. 2022;14(1):2035661. 10.1080/19490976.2022.2035661. 35184677 10.1080/19490976.2022.2035661 PMC8865257

[340] Tan S, Santolaya JL, Wright TF, Liu Q, Fujikawa T, Chi S, et al. Interaction between the gut microbiota and colonic enteroendocrine cells regulates host metabolism. Nat Metab. 2024;6(6):1076–91. 10.1038/s42255-024-01044-5. 38777856 10.1038/s42255-024-01044-5 PMC12001959

[341] Gribble FM, Reimann F. Enteroendocrine cells: chemosensors in the intestinal epithelium. Annu Rev Physiol. 2016;78:277–99. 10.1146/annurev-physiol-021115-105439. 26442437 10.1146/annurev-physiol-021115-105439

[342] Farzi A, Fröhlich EE, Holzer P. Gut microbiota and the neuroendocrine system. Neurother: J Am Soc Exp NeuroTher. 2018;15(1):5–22. 10.1007/s13311-017-0600-5. 10.1007/s13311-017-0600-5 PMC5794709 29380303

[343] Baars DP, Fondevila MF, Meijnikman AS, Nieuwdorp M. The central role of the gut microbiota in the pathophysiology and management of type 2 diabetes. Cell Host Microbe. 2024;32(8):1280–300. 10.1016/j.chom.2024.07.017. 39146799 10.1016/j.chom.2024.07.017

[344] Wu H, Tremaroli V, Schmidt C, Lundqvist A, Olsson LM, Krämer M, et al. The gut microbiota in prediabetes and diabetes: a population-based cross-sectional study. Cell Metab. 2020;32(3):379-390.e3. 10.1016/j.cmet.2020.06.011. 32652044 10.1016/j.cmet.2020.06.011

[345] Thomas C, Gioiello A, Noriega L, Strehle A, Oury J, Rizzo G, et al. TGR5-mediated bile acid sensing controls glucose homeostasis. Cell Metab. 2009;10(3):167–77. 10.1016/j.cmet.2009.08.001. 19723493 10.1016/j.cmet.2009.08.001 PMC2739652

[346] Perry RJ, Peng L, Barry NA, Cline GW, Zhang D, Cardone RL, et al. Acetate mediates a microbiome-brain-β-cell axis to promote metabolic syndrome. Nature. 2016;534(7606):213–7. 10.1038/nature18309. 27279214 10.1038/nature18309 PMC4922538

[347] McLean BA, Wong CK, Campbell JE, Hodson DJ, Trapp S, Drucker DJ. Revisiting the complexity of GLP-1 action from sites of synthesis to receptor activation. Endocr Rev. 2021;42(2):101–32. 10.1210/endrev/bnaa032. 33320179 10.1210/endrev/bnaa032 PMC7958144

[348] Vogt ÉL, Kowaltowski AJ. GLP-1, pancreatic β-cells, and insulin secretion: what we know and where we need to go. Diabetes. 2026;75(3):403–13. 10.2337/db25-0695. 41525103 10.2337/db25-0695

[349] McNelis JC, Lee YS, Mayoral R, van der Kant R, Johnson AMF, Wollam J, et al. GPR43 potentiates β-cell function in obesity. Diabetes. 2015;64(9):3203–17. 10.2337/db14-1938. 26023106 10.2337/db14-1938 PMC4542437

[350] Pedersen HK, Gudmundsdottir V, Nielsen HB, Hyotylainen T, Nielsen T, Jensen BAH, et al. Human gut microbes impact host serum metabolome and insulin sensitivity. Nature. 2016;535(7612):376–81. 10.1038/nature18646. 27409811 10.1038/nature18646

[351] Takeuchi T, Kubota T, Nakanishi Y, Tsugawa H, Suda W, Kwon ATJ, et al. Gut microbial carbohydrate metabolism contributes to insulin resistance. Nature. 2023;621(7978):389–95. 10.1038/s41586-023-06466-x. 37648852 10.1038/s41586-023-06466-x PMC10499599

[352] Canfora EE, Meex RCR, Venema K, Blaak EE. Gut microbial metabolites in obesity, NAFLD and T2DM. Nat Rev, Endocrinol. 2019;15(5):261–73. 10.1038/s41574-019-0156-z. 30670819 10.1038/s41574-019-0156-z

[353] McCarville JL, Chen GY, Cuevas VD, Troha K, Ayres JS. Microbiota metabolites in health and disease. Annu Rev Immunol. 2020;38:147–70. 10.1146/annurev-immunol-071219-125715. 32340573 10.1146/annurev-immunol-071219-125715

[354] Torres ER, Wilcox J, Tang WHW. Gut-heart axis: emerging therapies targeting trimethylamine N-oxide production. Gut Microbes. 18(1):2604868. 10.1080/19490976.2025.2604868. 10.1080/19490976.2025.2604868 PMC12758192 41454643

[355] Wang Z, Klipfell E, Bennett BJ, Koeth R, Levison BS, Dugar B, et al. Gut flora metabolism of phosphatidylcholine promotes cardiovascular disease. Nature. 2011;472(7341):57–63. 10.1038/nature09922. 21475195 10.1038/nature09922 PMC3086762

[356] Jin Y, Zhang SJ, Zhuang S, Li P, Miao H, Zhao YY. Microbiota-gut-kidney axis in health and renal disease. Int J Biol Sci. 2026;22(2):750–70. 10.7150/ijbs.125140. 41522358 10.7150/ijbs.125140 PMC12781074

[357] Lee JY, Mahan SP, Parente de Carvalho T, Nguyen H, Singai C, Camacho L, et al. Host-derived nitrate fuels indole production by escherichia coli to drive chronic kidney disease progression. Science. 2026;391(6791):1250–5. 10.1126/science.ady5217. 10.1126/science.ady5217 PMC13004173 41855331

[358] Sun ZH, Gong Q, Wang ZL, Li C, Wang JN, Yu JT, et al. Microbiota-gut-kidney axis and targeted therapeutic strategies in kidney diseases. Int J Biol Sci. 2026;22(3):1142–61. 10.7150/ijbs.124499. 41608641 10.7150/ijbs.124499 PMC12836503

[359] Snelson M, R Muralitharan R, Liu CF, Markó L, Forslund SK, Marques FZ, et al. Gut-heart axis: the role of gut microbiota and metabolites in heart failure. Circ Res. 2025;136(11):1382–406. 10.1161/CIRCRESAHA.125.325516. 10.1161/CIRCRESAHA.125.325516 PMC12101525 40403109

[360] Budden KF, Gellatly SL, Wood DLA, Cooper MA, Morrison M, Hugenholtz P, et al. Emerging pathogenic links between microbiota and the gut-lung axis. Nat Rev Microbiol. 2017;15(1):55–63. 10.1038/nrmicro.2016.142. 27694885 10.1038/nrmicro.2016.142

[361] Saint-Criq V, Lugo-Villarino G, Thomas M. Dysbiosis, malnutrition and enhanced gut-lung axis contribute to age-related respiratory diseases. Ageing Res Rev. 2021;66:101235. 10.1016/j.arr.2020.101235. 33321253 10.1016/j.arr.2020.101235

[362] Wang L, Cai Y, Garssen J, Henricks PAJ, Folkerts G, Braber S. The bidirectional gut-lung axis in chronic obstructive pulmonary disease. Am J Respir Crit Care Med. 2023;207(9):1145–60. 10.1164/rccm.202206-1066TR. 36883945 10.1164/rccm.202206-1066TR PMC10161745

[363] Wypych TP, Wickramasinghe LC, Marsland BJ. The influence of the microbiome on respiratory health. Nat Immunol. 2019;20(10):1279–90. 10.1038/s41590-019-0451-9. 31501577 10.1038/s41590-019-0451-9

[364] Mahmud MR, Akter S, Tamanna SK, Mazumder L, Esti IZ, Banerjee S, et al. Impact of gut microbiome on skin health: gut-skin axis observed through the lenses of therapeutics and skin diseases. Gut Microbes. 2022;14(1):2096995. 10.1080/19490976.2022.2096995. 35866234 10.1080/19490976.2022.2096995 PMC9311318

[365] You Y, Xiang T, Yang C, Xiao S, Tang Y, Luo G, et al. Interactions between the gut microbiota and immune cell dynamics: novel insights into the gut-bone axis. Gut Microbes. 2025;17(1):2545417. 10.1080/19490976.2025.2545417. 40873417 10.1080/19490976.2025.2545417 PMC12396131

[366] Qi X, Yun C, Pang Y, Qiao J. The impact of the gut microbiota on the reproductive and metabolic endocrine system. Gut Microbes. 2021;13(1):1–21. 10.1080/19490976.2021.1894070. 33722164 10.1080/19490976.2021.1894070 PMC7971312

[367] Senthilkumar H, Arumugam M. Gut microbiota: a hidden player in polycystic ovary syndrome. J Transl Med. 2025;23(1):443. 10.1186/s12967-025-06315-7. 40234859 10.1186/s12967-025-06315-7 PMC11998441

[368] Chen X, Zuo Z, Xiao B, Zhao F. Modulating the gut-reproductive tract axis: microbial influence on gynaecological cancer pathogenesis and treatment. Gut. 2026;gutjnl-2025–337778. 10.1136/gutjnl-2025-337778. 10.1136/gutjnl-2025-337778 41667242

[369] Porcari S, Mullish BH, Asnicar F, Ng SC, Zhao L, Hansen R, et al. International consensus statement on microbiome testing in clinical practice. lancet, Gastroenterol Hepatol. 2025;10(2):154–67. 10.1016/S2468-1253(24)00311-X. 10.1016/S2468-1253(24)00311-X PMC12343204 39647502

[370] Tegegne HA, Savidge TC. Gut microbiome metagenomics in clinical practice: bridging the gap between research and precision medicine. Gut Microbes. 2025;17(1):2569739. 10.1080/19490976.2025.2569739. 41137523 10.1080/19490976.2025.2569739 PMC12562794

[371] Zheng J, Sun Q, Zhang M, Liu C, Su Q, Zhang L, et al. Noninvasive, microbiome-based diagnosis of inflammatory bowel disease. Nat Med. 2024;30(12):3555–67. 10.1038/s41591-024-03280-4. 39367251 10.1038/s41591-024-03280-4 PMC11645270

[372] Akiyama S, Nishijima S, Kojima Y, Kimura M, Ohsugi M, Ueki K, et al. Multi-biome analysis identifies distinct gut microbial signatures and their crosstalk in ulcerative colitis and crohn’s disease. Nat Commun. 2024;15(1):10291. 10.1038/s41467-024-54797-8. 39604394 10.1038/s41467-024-54797-8 PMC11603027

[373] Vich Vila A, Zhang J, Liu M, Faber KN, Weersma RK. Untargeted faecal metabolomics for the discovery of biomarkers and treatment targets for inflammatory bowel diseases. Gut. 2024;73(11):1909–20. 10.1136/gutjnl-2023-329969. 39002973 10.1136/gutjnl-2023-329969 PMC11503092

[374] Thomas AM, Manghi P, Asnicar F, Pasolli E, Armanini F, Zolfo M, et al. Metagenomic analysis of colorectal cancer datasets identifies cross-cohort microbial diagnostic signatures and a link with choline degradation. Nat Med. 2019;25(4):667–78. 10.1038/s41591-019-0405-7. 30936548 10.1038/s41591-019-0405-7 PMC9533319

[375] Lee JWJ, Plichta DR, Asher S, Delsignore M, Jeong T, McGoldrick J, et al. Association of distinct microbial signatures with premalignant colorectal adenomas. Cell Host Microbe. 2023;31(5):827-838.e3. 10.1016/j.chom.2023.04.007. 37130517 10.1016/j.chom.2023.04.007 PMC10477964

[376] Turpin W, Lee SH, Croitoru K. Gut microbiome signature in predisease phase of inflammatory bowel disease: prediction to pathogenesis to prevention. Gastroenterology. 2025;168(5):902–13. 10.1053/j.gastro.2025.01.004. 39914464 10.1053/j.gastro.2025.01.004

[377] Kennedy MS, Chang EB. Emerging concepts and shifting paradigms for understanding the microbial basis of inflammatory bowel diseases. J Clin Invest. 135(17):e193969. 10.1172/JCI193969. 10.1172/JCI193969 PMC12404754 40892512

[378] Piccinno G, Thompson KN, Manghi P, Ghazi AR, Thomas AM, Blanco-Míguez A, et al. Pooled analysis of 3,741 stool metagenomes from 18 cohorts for cross-stage and strain-level reproducible microbial biomarkers of colorectal cancer. Nat Med. 2025;31(7):2416–29. 10.1038/s41591-025-03693-9. 40461820 10.1038/s41591-025-03693-9 PMC12283368

[379] Song D, Feng G, Ma Y, Shi Y, Qian C, Wang C, et al. Gut microbiome predicts personalized responses to dietary fiber in prediabetes: a randomized, open-label trial. Nat Commun. 2025;16(1):11506. 10.1038/s41467-025-66498-x. 41390484 10.1038/s41467-025-66498-x PMC12749054

[380] Silva CAC, Fidelle M, Almonte AA, Derosa L, Zitvogel L. Gut microbiota-related biomarkers in immuno-oncology. Annu Rev Pharmacol Toxicol. 2025;65(1):333–54. 10.1146/annurev-pharmtox-061124-102218. 39259979 10.1146/annurev-pharmtox-061124-102218

[381] Bolte LA, Björk JR, Gacesa R, Weersma RK. Pharmacomicrobiomics: the role of the gut microbiome in immunomodulation and cancer therapy. Gastroenterology. 2025;169(5):813–27. 10.1053/j.gastro.2025.04.025. 40381958 10.1053/j.gastro.2025.04.025

[382] Lin Y, Xie M, Lau HCH, Zeng R, Zhang R, Wang L, et al. Effects of gut microbiota on immune checkpoint inhibitors in multi-cancer and as microbial biomarkers for predicting therapeutic response. Med. 2025;6(3):100530. 10.1016/j.medj.2024.10.007. 39515321 10.1016/j.medj.2024.10.007

[383] Lee CH, Steiner T, Petrof EO, Smieja M, Roscoe D, Nematallah A, et al. Frozen vs fresh fecal microbiota transplantation and clinical resolution of diarrhea in patients with recurrent clostridium difficile infection: a randomized clinical trial. JAMA. 2016;315(2):142–9. 10.1001/jama.2015.18098. 26757463 10.1001/jama.2015.18098

[384] Khanna S, Assi M, Lee C, Yoho D, Louie T, Knapple W, et al. Efficacy and safety of RBX2660 in PUNCH CD3, a phase III, randomized, double-blind, placebo-controlled trial with a bayesian primary analysis for the prevention of recurrent clostridioides difficile infection. Drugs. 2022;82(15):1527–38. 10.1007/s40265-022-01797-x. 36287379 10.1007/s40265-022-01797-x PMC9607700

[385] Claypool J, Lindved G, Myers PN, Ward T, Nielsen HB, Blount KF. Microbiome compositional changes and clonal engraftment in a phase 3 trial of fecal microbiota, live-jslm for recurrent clostridioides difficile infection. Gut Microbes. 2025;17(1):2520412. 10.1080/19490976.2025.2520412. 40552763 10.1080/19490976.2025.2520412 PMC12931727

[386] Feuerstadt P, Louie TJ, Lashner B, Wang EEL, Diao L, Bryant JA, et al. SER-109, an oral microbiome therapy for recurrent clostridioides difficile infection. N Engl J Med. 2022;386(3):220–9. 10.1056/NEJMoa2106516. 35045228 10.1056/NEJMoa2106516

[387] Louie T, Golan Y, Khanna S, Bobilev D, Erpelding N, Fratazzi C, et al. VE303, a defined bacterial consortium, for prevention of recurrent clostridioides difficile infection: a randomized clinical trial. JAMA. 2023;329(16):1356–66. 10.1001/jama.2023.4314. 37060545 10.1001/jama.2023.4314 PMC10105904

[388] Paramsothy S, Kamm MA, Kaakoush NO, Walsh AJ, van den Bogaerde J, Samuel D, et al. Multidonor intensive faecal microbiota transplantation for active ulcerative colitis: a randomised placebo-controlled trial. Lancet. 2017;389(10075):1218–28. 10.1016/S0140-6736(17)30182-4. 28214091 10.1016/S0140-6736(17)30182-4

[389] Paramsothy S, Nielsen S, Kamm MA, Deshpande NP, Faith JJ, Clemente JC, et al. Specific bacteria and metabolites associated with response to fecal microbiota transplantation in patients with ulcerative colitis. Gastroenterology. 2019;156(5):1440-1454.e2. 10.1053/j.gastro.2018.12.001. 30529583 10.1053/j.gastro.2018.12.001

[390] Moayyedi P, Surette MG, Kim PT, Libertucci J, Wolfe M, Onischi C, et al. Fecal microbiota transplantation induces remission in patients with active ulcerative colitis in a randomized controlled trial. Gastroenterology. 2015;149(1):102-109.e6. 10.1053/j.gastro.2015.04.001. 25857665 10.1053/j.gastro.2015.04.001

[391] Gogokhia L, Tran N, Grier A, Nagayama M, Xiang G, Funez-dePagnier G, et al. Donor composition and fiber promote strain engraftment in a randomized controlled trial of fecal microbiota transplant for ulcerative colitis. Med. 2025;6(9):100707. 10.1016/j.medj.2025.100707. 40460824 10.1016/j.medj.2025.100707 PMC12353604

[392] S P, C C, V H, D R, G Q, A S, et al. Fecal microbiota transplantation plus pembrolizumab and axitinib in metastatic renal cell carcinoma: the randomized phase 2 TACITO trial. Nat Med. 2026;32(4). 10.1038/s41591-025-04189-2. Cited 2026 Apr 29. 10.1038/s41591-025-04189-2 PMC13099650 41606119

[393] Davar D, Dzutsev AK, McCulloch JA, Rodrigues RR, Chauvin JM, Morrison RM, et al. Fecal microbiota transplant overcomes resistance to anti-PD-1 therapy in melanoma patients. Science. 2021;371(6529):595–602. 10.1126/science.abf3363. 33542131 10.1126/science.abf3363 PMC8097968

[394] Mego M, Chovanec J, Vochyanova-Andrezalova I, Konkolovsky P, Mikulova M, Reckova M, et al. Prevention of irinotecan induced diarrhea by probiotics: a randomized double blind, placebo controlled pilot study. Complement Ther Med. 2015;23(3):356–62. 10.1016/j.ctim.2015.03.008. 26051570 10.1016/j.ctim.2015.03.008

[395] Stene C, Xu J, Fallone de Andrade S, Palmquist I, Molin G, Ahrné S, et al. Synbiotics protected radiation-induced tissue damage in rectal cancer patients: a controlled trial. Clin Nutr. 2025;49:33–41. 10.1016/j.clnu.2025.03.025. 40250086 10.1016/j.clnu.2025.03.025

[396] Zaharuddin L, Mokhtar NM, Muhammad Nawawi KN, Raja Ali RA. A randomized double-blind placebo-controlled trial of probiotics in post-surgical colorectal cancer. BMC Gastroenterol. 2019;19(1):131. 10.1186/s12876-019-1047-4. 31340751 10.1186/s12876-019-1047-4 PMC6657028

[397] Depommier C, Everard A, Druart C, Plovier H, Van Hul M, Vieira-Silva S, et al. Supplementation with akkermansia muciniphila in overweight and obese human volunteers: a proof-of-concept exploratory study. Nat Med. 2019;25(7):1096–103. 10.1038/s41591-019-0495-2. 31263284 10.1038/s41591-019-0495-2 PMC6699990

[398] Mocanu V, Zhang Z, Deehan EC, Kao DH, Hotte N, Karmali S, et al. Fecal microbial transplantation and fiber supplementation in patients with severe obesity and metabolic syndrome: a randomized double-blind, placebo-controlled phase 2 trial. Nat Med. 2021;27(7):1272–9. 10.1038/s41591-021-01399-2. 34226737 10.1038/s41591-021-01399-2

[399] Böhn L, Störsrud S, Liljebo T, Collin L, Lindfors P, Törnblom H, et al. Diet low in FODMAPs reduces symptoms of irritable bowel syndrome as well as traditional dietary advice: a randomized controlled trial. Gastroenterology. 2015;149(6):1399-1407.e2. 10.1053/j.gastro.2015.07.054. 26255043 10.1053/j.gastro.2015.07.054

[400] Bennet SMP, Böhn L, Störsrud S, Liljebo T, Collin L, Lindfors P, et al. Multivariate modelling of faecal bacterial profiles of patients with IBS predicts responsiveness to a diet low in FODMAPs. Gut. 2018;67(5):872–81. 10.1136/gutjnl-2016-313128. 28416515 10.1136/gutjnl-2016-313128

[401] Srivastava S, Basak U, Naghibi M, Vijayakumar V, Parihar R, Patel J, et al. A randomized double-blind, placebo-controlled trial to evaluate the safety and efficacy of live bifidobacterium longum CECT 7347 (ES1) and heat-treated bifidobacterium longum CECT 7347 (HT-ES1) in participants with diarrhea-predominant irritable bowel syndrome. Gut Microbes. 16(1):2338322. 10.1080/19490976.2024.2338322. 10.1080/19490976.2024.2338322 PMC11028008 38630015

[402] Dhiman RK, Rana B, Agrawal S, Garg A, Chopra M, Thumburu KK, et al. Probiotic VSL#3 reduces liver disease severity and hospitalization in patients with cirrhosis: a randomized, controlled trial. Gastroenterology. 2014;147(6):1327-1337.e3. 10.1053/j.gastro.2014.08.031. 25450083 10.1053/j.gastro.2014.08.031

[403] Scorletti E, Afolabi PR, Miles EA, Smith DE, Almehmadi A, Alshathry A, et al. Synbiotics alter fecal microbiomes, but not liver fat or fibrosis, in a randomized trial of patients with nonalcoholic fatty liver disease. Gastroenterology. 2020;158(6):1597-1610.e7. 10.1053/j.gastro.2020.01.031. 31987796 10.1053/j.gastro.2020.01.031 PMC7613160

[404] Bruggeman A, Vandendriessche C, Hamerlinck H, De Looze D, Tate DJ, Vuylsteke M, et al. Safety and efficacy of faecal microbiota transplantation in patients with mild to moderate parkinson’s disease (GUT-PARFECT): a double-blind, placebo-controlled, randomised, phase 2 trial. EClinicalMedicine. 2024;71:102563. 10.1016/j.eclinm.2024.102563. 38686220 10.1016/j.eclinm.2024.102563 PMC11056595

[405] Nikolova VL, Cleare AJ, Young AH, Stone JM. Acceptability, tolerability, and estimates of putative treatment effects of probiotics as adjunctive treatment in patients with depression: a randomized clinical trial. JAMA Psychiat. 2023;80(8):842–7. 10.1001/jamapsychiatry.2023.1817. 10.1001/jamapsychiatry.2023.1817 PMC10267847 37314797

[406] Vockley J, Sondheimer N, Puurunen M, Diaz GA, Ginevic I, Grange DK, et al. Efficacy and safety of a synthetic biotic for treatment of phenylketonuria: a phase 2 clinical trial. Nat Metab. 2023;5(10):1685–90. 10.1038/s42255-023-00897-6. 37770764 10.1038/s42255-023-00897-6

[407] Ross FC, Patangia D, Grimaud G, Lavelle A, Dempsey EM, Ross RP, et al. The interplay between diet and the gut microbiome: implications for health and disease. Nat Rev Microbiol. 2024;22(11):671–86. 10.1038/s41579-024-01068-4. 39009882 10.1038/s41579-024-01068-4

[408] Sanz Y, Cryan JF, Deschasaux-Tanguy M, Elinav E, Lambrecht R, Veiga P. The gut microbiome connects nutrition and human health. Nat Rev, Gastroenterol Hepatol. 2025;22(8):534–55. 10.1038/s41575-025-01077-5. 40468006 10.1038/s41575-025-01077-5

[409] Duncanson K, Williams G, Hoedt EC, Collins CE, Keely S, Talley NJ. Diet-microbiota associations in gastrointestinal research: a systematic review. Gut Microbes. 2024;16(1):2350785. 10.1080/19490976.2024.2350785. 38725230 10.1080/19490976.2024.2350785 PMC11093048

[410] Segev T, Barak D, Zahavi L, Godneva A, Rein M, Krongauz D, et al. Diet-microbiome associations in 10,068 individuals from the human phenotype project to guide personalized nutrition. Nat Med. 2026. 10.1038/s41591-026-04312-x. 10.1038/s41591-026-04312-x 41872600

[411] Koletic C, Mrad A, Martin A, Devkota S. Diet’s impact on gut microbial assemblage in health and disease. J Clin Invest. 135(11):e184319. 10.1172/JCI184319. 10.1172/JCI184319 PMC12126223 40454482

[412] Nakatsu G, Andreeva N, MacDonald MH, Garrett WS. Interactions between diet and gut microbiota in cancer. Nat Microbiol. 2024;9(7):1644–54. 10.1038/s41564-024-01736-4. 38907007 10.1038/s41564-024-01736-4

[413] Ananthakrishnan AN, Whelan K, Allegretti JR, Sokol H. Diet and microbiome-directed therapy 2.0 for IBD. Clin Gastroenterol Hepatol: Off Clin Pract J Am Gastroenterol Assoc. 2025;23(3):406–18. 10.1016/j.cgh.2024.05.049. 10.1016/j.cgh.2024.05.049 38992408

[414] Hashash JG, Elkins J, Lewis JD, Binion DG. AGA clinical practice update on diet and nutritional therapies in patients with inflammatory bowel disease: expert review. Gastroenterology. 2024;166(3):521–32. 10.1053/j.gastro.2023.11.303. 38276922 10.1053/j.gastro.2023.11.303

[415] Black CJ, Staudacher HM, Ford AC. Efficacy of a low FODMAP diet in irritable bowel syndrome: systematic review and network meta-analysis. Gut. 2022;71(6):1117–26. 10.1136/gutjnl-2021-325214. 34376515 10.1136/gutjnl-2021-325214

[416] Nybacka S, Törnblom H, Josefsson A, Hreinsson JP, Böhn L, Frändemark Å, et al. A low FODMAP diet plus traditional dietary advice versus a low-carbohydrate diet versus pharmacological treatment in irritable bowel syndrome (CARIBS): a single-centre, single-blind, randomised controlled trial. Lancet Gastroenterol Hepatol. 2024;9(6):507–20. 10.1016/S2468-1253(24)00045-1. 38643782 10.1016/S2468-1253(24)00045-1

[417] Meslier V, Laiola M, Roager HM, De Filippis F, Roume H, Quinquis B, et al. Mediterranean diet intervention in overweight and obese subjects lowers plasma cholesterol and causes changes in the gut microbiome and metabolome independently of energy intake. Gut. 2020;69(7):1258–68. 10.1136/gutjnl-2019-320438. 32075887 10.1136/gutjnl-2019-320438 PMC7306983

[418] Muscogiuri G, Verde L, Sulu C, Katsiki N, Hassapidou M, Frias-Toral E, et al. Mediterranean diet and obesity-related disorders: what is the evidence? Curr Obes Rep. 2022;11(4):287–304. 10.1007/s13679-022-00481-1. 36178601 10.1007/s13679-022-00481-1 PMC9729142

[419] Rinott E, Meir AY, Tsaban G, Zelicha H, Kaplan A, Knights D, et al. The effects of the green-mediterranean diet on cardiometabolic health are linked to gut microbiome modifications: a randomized controlled trial. Genome Med. 2022;14(1):29. 10.1186/s13073-022-01015-z. 35264213 10.1186/s13073-022-01015-z PMC8908597

[420] Li H, Zhang L, Li J, Wu Q, Qian L, He J, et al. Resistant starch intake facilitates weight loss in humans by reshaping the gut microbiota. Nat Metab. 2024;6(3):578–97. 10.1038/s42255-024-00988-y. 38409604 10.1038/s42255-024-00988-y PMC10963277

[421] Wu Z, Huang S, Li T, Li N, Han D, Zhang B, et al. Gut microbiota from green tea polyphenol-dosed mice improves intestinal epithelial homeostasis and ameliorates experimental colitis. Microbiome. 2021;9(1):184. 10.1186/s40168-021-01115-9. 34493333 10.1186/s40168-021-01115-9 PMC8424887

[422] Wang W, Fan Z, Yan Q, Pan T, Luo J, Wei Y, et al. Gut microbiota determines the fate of dietary fiber-targeted interventions in host health. Gut Microbes. 2024;16(1):2416915. 10.1080/19490976.2024.2416915. 39418223 10.1080/19490976.2024.2416915 PMC11487953

[423] Ji J, Jin W, Liu SJ, Jiao Z, Li X. Probiotics, prebiotics, and postbiotics in health and disease. MedComm. 2023;4(6):e420. 10.1002/mco2.420. 37929014 10.1002/mco2.420 PMC10625129

[424] Sanders ME, Hill C. The microbiome: an actor or stage for the beneficial action of probiotics, prebiotics, synbiotics, and postbiotics? Cell Host Microbe. 2025;33(6):777–89. 10.1016/j.chom.2025.04.017. 40505618 10.1016/j.chom.2025.04.017

[425] Hill C, Guarner F, Reid G, Gibson GR, Merenstein DJ, Pot B, et al. Expert consensus document. The international scientific association for probiotics and prebiotics consensus statement on the scope and appropriate use of the term probiotic. Nat Rev Gastroenterol Hepatol. 2014;11(8):506–14. 10.1038/nrgastro.2014.66. 24912386 10.1038/nrgastro.2014.66

[426] Lukasik J, Dierikx T, Besseling-van der Vaart I, de Meij T, Szajewska H, Multispecies Probiotic In Aad Study Group. Multispecies probiotic for the prevention of antibiotic-associated diarrhea in children: a randomized clinical trial. JAMA Pediatr. 2022;176(9):860–6. 10.1001/jamapediatrics.2022.1973. 10.1001/jamapediatrics.2022.1973 PMC9214631 35727573

[427] Esmaeilinezhad Z, Ghosh NR, Walsh CM, Steen JP, Burgman AM, Mertz D, et al. Probiotics for the prevention of clostridioides difficile-associated diarrhea in adults and children. Cochrane Database Syst Rev. 2025;9(9):CD006095. 10.1002/14651858.CD006095.pub5. 10.1002/14651858.CD006095.pub5 PMC12424122 40931979

[428] Vallejos OP, Bueno SM, Kalergis AM. Probiotics in inflammatory bowel disease: microbial modulation and therapeutic prospects. Trends Mol Med. 2025;31(8):731–42. 10.1016/j.molmed.2024.12.005. 39814640 10.1016/j.molmed.2024.12.005

[429] Estevinho MM, Yuan Y, Rodríguez-Lago I, Sousa-Pimenta M, Dias CC, Barreiro-de Acosta M, et al. Efficacy and safety of probiotics in IBD: an overview of systematic reviews and updated meta-analysis of randomized controlled trials. United Eur Gastroenterol J. 2024;12(7):960–81. 10.1002/ueg2.12636. 10.1002/ueg2.12636 PMC11497663 39106167

[430] Goodoory VC, Khasawneh M, Black CJ, Quigley EMM, Moayyedi P, Ford AC. Efficacy of probiotics in irritable bowel syndrome: systematic review and meta-analysis. Gastroenterology. 2023;165(5):1206–18. 10.1053/j.gastro.2023.07.018. 37541528 10.1053/j.gastro.2023.07.018

[431] Gibson GR, Hutkins R, Sanders ME, Prescott SL, Reimer RA, Salminen SJ, et al. Expert consensus document: the international scientific association for probiotics and prebiotics (ISAPP) consensus statement on the definition and scope of prebiotics. Nat Rev, Gastroenterol Hepatol. 2017;14(8):491–502. 10.1038/nrgastro.2017.75. 28611480 10.1038/nrgastro.2017.75

[432] Hutkins R, Walter J, Gibson GR, Bedu-Ferrari C, Scott K, Tancredi DJ, et al. Classifying compounds as prebiotics — scientific perspectives and recommendations. Nat Rev Gastroenterol Hepatol. 2025;22(1):54–70. 10.1038/s41575-024-00981-6. 39358591 10.1038/s41575-024-00981-6

[433] Lai H, Li Y, He Y, Chen F, Mi B, Li J, et al. Effects of dietary fibers or probiotics on functional constipation symptoms and roles of gut microbiota: a double-blinded randomized placebo trial. Gut Microbes. 15(1):2197837. 10.1080/19490976.2023.2197837. 10.1080/19490976.2023.2197837 PMC10120550 37078654

[434] Chalotra R, Gupta T, Kumar A, Gupta A, Kumar S, Singh TG, et al. Prebiotics, probiotics, and postbiotics in modulating gut microbiota: emerging therapeutic approaches for metabolic syndrome. Curr Obes Rep. 2026;15(1):9. 10.1007/s13679-026-00686-8. 41661460 10.1007/s13679-026-00686-8

[435] Paone P, Suriano F, Jian C, Korpela K, Delzenne NM, Van Hul M, et al. Prebiotic oligofructose protects against high-fat diet-induced obesity by changing the gut microbiota, intestinal mucus production, glycosylation and secretion. Gut Microbes. 2022;14(1):2152307. 10.1080/19490976.2022.2152307. 36448728 10.1080/19490976.2022.2152307 PMC9715274

[436] Swanson KS, Gibson GR, Hutkins R, Reimer RA, Reid G, Verbeke K, et al. The international scientific association for probiotics and prebiotics (ISAPP) consensus statement on the definition and scope of synbiotics. Nat Rev, Gastroenterol Hepatol. 2020;17(11):687–701. 10.1038/s41575-020-0344-2. 32826966 10.1038/s41575-020-0344-2 PMC7581511

[437] Wu Y, Zhang X, Liu X, Zhao Z, Tao S, Xu Q, et al. Galactooligosaccharides and limosilactobacillus reuteri synergistically alleviate gut inflammation and barrier dysfunction by enriching bacteroides acidifaciens for pentadecanoic acid biosynthesis. Nat Commun. 2024;15(1):9291. 10.1038/s41467-024-53144-1. 39468026 10.1038/s41467-024-53144-1 PMC11519483

[438] Bock PM, Telo GH, Ramalho R, Sbaraini M, Leivas G, Martins AF, et al. The effect of probiotics, prebiotics or synbiotics on metabolic outcomes in individuals with diabetes: a systematic review and meta-analysis. Diabetologia. 2021;64(1):26–41. 10.1007/s00125-020-05295-1. 33047170 10.1007/s00125-020-05295-1

[439] Salminen S, Collado MC, Endo A, Hill C, Lebeer S, Quigley EMM, et al. The international scientific association of probiotics and prebiotics (ISAPP) consensus statement on the definition and scope of postbiotics. Nat Rev, Gastroenterol Hepatol. 2021;18(9):649–67. 10.1038/s41575-021-00440-6. 33948025 10.1038/s41575-021-00440-6 PMC8387231

[440] Mosca A, Abreu Y Abreu AT, Gwee KA, Ianiro G, Tack J, Nguyen TVH, et al. The clinical evidence for postbiotics as microbial therapeutics. Gut Microbes. 2022;14(1):2117508. 10.1080/19490976.2022.2117508. 10.1080/19490976.2022.2117508 PMC9542959 36184735

[441] Arellano-García LI, Portillo MP, Martínez JA, Courtois A, Milton-Laskibar I. Postbiotics for the management of obesity, insulin resistance/type 2 diabetes and NAFLD. Beyond microbial viability. Crit Rev Food Sci Nutr. 2025;65(29):6209–32. 10.1080/10408398.2024.2437143. 39644489 10.1080/10408398.2024.2437143

[442] Guo S, Ma T, Kwok LY, Quan K, Li B, Wang H, et al. Effects of postbiotics on chronic diarrhea in young adults: a randomized, double-blind, placebo-controlled crossover trial assessing clinical symptoms, gut microbiota, and metabolite profiles. Gut Microbes. 2024;16(1):2395092. 10.1080/19490976.2024.2395092. 39189588 10.1080/19490976.2024.2395092 PMC11352714

[443] Vinderola G, Sanders ME, Salminen S, Szajewska H. Postbiotics: the concept and their use in healthy populations. Front Nutr. 2022;9:1002213. 10.3389/fnut.2022.1002213. 36570166 10.3389/fnut.2022.1002213 PMC9780264

[444] Porcari S, Benech N, Valles-Colomer M, Segata N, Gasbarrini A, Cammarota G, et al. Key determinants of success in fecal microbiota transplantation: from microbiome to clinic. Cell Host Microbe. 2023;31(5):712–33. 10.1016/j.chom.2023.03.020. 37167953 10.1016/j.chom.2023.03.020

[445] Hoffmann DE, Javitt GH, Kelly CR, Keller JJ, Baunwall SMD, Hvas CL. Fecal microbiota transplantation: a tale of two regulatory pathways. Gut Microbes. 17(1):2493901. 10.1080/19490976.2025.2493901. 10.1080/19490976.2025.2493901 PMC12054926 40302307

[446] Kim DY, Lee SY, Lee JY, Whon TW, Lee JY, Jeon CO, et al. Gut microbiome therapy: fecal microbiota transplantation vs live biotherapeutic products. Gut Microbes. 2024;16(1):2412376. 10.1080/19490976.2024.2412376. 39377231 10.1080/19490976.2024.2412376 PMC11469438

[447] Peery AF, Kelly CR, Kao D, Vaughn BP, Lebwohl B, Singh S, et al. AGA clinical practice guideline on fecal microbiota-based therapies for select gastrointestinal diseases. Gastroenterology. 2024;166(3):409–34. 10.1053/j.gastro.2024.01.008. 38395525 10.1053/j.gastro.2024.01.008

[448] Yadegar A, Pakpoor S, Ibrahim FF, Nabavi-Rad A, Cook L, Walter J, et al. Beneficial effects of fecal microbiota transplantation in recurrent clostridioides difficile infection. Cell Host Microbe. 2023;31(5):695–711. 10.1016/j.chom.2023.03.019. 37167952 10.1016/j.chom.2023.03.019 PMC10966711

[449] Hvas CL, Dahl Jørgensen SM, Jørgensen SP, Storgaard M, Lemming L, Hansen MM, et al. Fecal microbiota transplantation is superior to fidaxomicin for treatment of recurrent clostridium difficile infection. Gastroenterology. 2019;156(5):1324-1332.e3. 10.1053/j.gastro.2018.12.019. 30610862 10.1053/j.gastro.2018.12.019

[450] Benech N, Barbut F, Fitzpatrick F, Krutova M, Davies K, Druart C, et al. Update on microbiota-derived therapies for recurrent clostridioides difficile infections. Clin Microbiol Infect: Off Publ Eur Soc Clin Microbiol Infect Dis. 2024;30(4):462–8. 10.1016/j.cmi.2023.12.007. 10.1016/j.cmi.2023.12.007 38101472

[451] Haifer C, Paramsothy S, Kaakoush NO, Saikal A, Ghaly S, Yang T, et al. Lyophilised oral faecal microbiota transplantation for ulcerative colitis (LOTUS): a randomised, double-blind, placebo-controlled trial. Lancet, Gastroenterol Hepatol. 2022;7(2):141–51. 10.1016/S2468-1253(21)00400-3. 34863330 10.1016/S2468-1253(21)00400-3

[452] Costello SP, Hughes PA, Waters O, Bryant RV, Vincent AD, Blatchford P, et al. Effect of fecal microbiota transplantation on 8-week remission in patients with ulcerative colitis: a randomized clinical trial. JAMA. 2019;321(2):156–64. 10.1001/jama.2018.20046. 30644982 10.1001/jama.2018.20046 PMC6439766

[453] Luu LDW, Pandey A, Paramsothy S, Ngo C, Castaño-Rodríguez N, Liu C, et al. Profiling the colonic mucosal response to fecal microbiota transplantation identifies a role for GBP5 in colitis in humans and mice. Nat Commun. 2024;15(1):2645. 10.1038/s41467-024-46983-5. 38531874 10.1038/s41467-024-46983-5 PMC10965925

[454] Bénard MV, de Goffau MC, Blonk J, Hugenholtz F, van Buuren J, Paramsothy S, et al. Gut microbiota features in relation to fecal microbiota transplantation outcome in ulcerative colitis: a systematic review and meta-analysis. Clin Gastroenterol Hepatol: Off Clin Pract J Am Gastroenterol Assoc. 2025;23(10):1719–36. 10.1016/j.cgh.2024.10.001. 10.1016/j.cgh.2024.10.001 39442743

[455] Feng J, Chen Y, Liu Y, Lin L, Lin X, Gong W, et al. Efficacy and safety of fecal microbiota transplantation in the treatment of ulcerative colitis: a systematic review and meta-analysis. Sci Rep. 2023;13(1):14494. 10.1038/s41598-023-41182-6. 37661203 10.1038/s41598-023-41182-6 PMC10475461

[456] Bajaj JS, Fagan A, Gavis EA, Sterling RK, Gallagher ML, Lee H, et al. Microbiota transplant for hepatic encephalopathy in cirrhosis: the THEMATIC trial. J Hepatol. 2025;83(1):81–91. 10.1016/j.jhep.2024.12.047. 39800192 10.1016/j.jhep.2024.12.047

[457] Bajaj JS, Kassam Z, Fagan A, Gavis EA, Liu E, Cox IJ, et al. Fecal microbiota transplant from a rational stool donor improves hepatic encephalopathy: a randomized clinical trial. Hepatology. 2017;66(6):1727–38. 10.1002/hep.29306. 28586116 10.1002/hep.29306 PMC6102730

[458] S R, L S, M E, D P, Ss M, T G, et al. Fecal microbiota transplantation to prevent acute graft-versus-host disease: pre-planned interim analysis of donor effect. Nat Commun. 2025;16(1). 10.1038/s41467-025-56375-y. Cited 2026 Mar 29. 10.1038/s41467-025-56375-y PMC11762788 39863610

[459] van Lier YF, Davids M, Haverkate NJE, de Groot PF, Donker ML, Meijer E, et al. Donor fecal microbiota transplantation ameliorates intestinal graft-versus-host disease in allogeneic hematopoietic cell transplant recipients. Sci Transl Med. 2020;12(556):eaaz8926. 10.1126/scitranslmed.aaz8926. 10.1126/scitranslmed.aaz8926 32801142

[460] Fan L, Chen J, Zhang Q, Ren J, Chen Y, Yang J, et al. Fecal microbiota transplantation for hypertension: an exploratory, multicenter, randomized, blinded, placebo-controlled trial. Microbiome. 2025;13(1):133. 10.1186/s40168-025-02118-6. 40410854 10.1186/s40168-025-02118-6 PMC12100813

[461] Routy B, Lenehan JG, Miller WH, Jamal R, Messaoudene M, Daisley BA, et al. Fecal microbiota transplantation plus anti-PD-1 immunotherapy in advanced melanoma: a phase I trial. Nat Med. 2023;29(8):2121–32. 10.1038/s41591-023-02453-x. 37414899 10.1038/s41591-023-02453-x

[462] Yang Y, An Y, Dong Y, Chu Q, Wei J, Wang B, et al. Fecal microbiota transplantation: no longer cinderella in tumour immunotherapy. eBioMedicine. 2024;100:104967. 10.1016/j.ebiom.2024.104967. 10.1016/j.ebiom.2024.104967 PMC10831174 38241975

[463] Lynch LE, Lahowetz R, Maresso C, Terwilliger A, Pizzini J, Melendez Hebib V, et al. Present and future of microbiome-targeting therapeutics. J Clin Invest. 2025;135(11):e184323. 10.1172/JCI184323. 40454480 10.1172/JCI184323 PMC12126222

[464] Tseng CH, Wong S, Yu J, Lee YY, Terauchi J, Lai HC, et al. Development of live biotherapeutic products: a position statement of Asia-pacific microbiota consortium. Gut. 2025;74(5):706–13. 10.1136/gutjnl-2024-334501. 40011030 10.1136/gutjnl-2024-334501 PMC12013581

[465] Kim MS, Bisanz JE. Design and application of synthetic human gut microbial communities. Gut Microbes. 2025;17(1):2575923. 10.1080/19490976.2025.2575923. 41239968 10.1080/19490976.2025.2575923 PMC12622336

[466] Menon R, Bhattarai SK, Crossette E, Prince AL, Olle B, Silber JL, et al. Multi-omic profiling a defined bacterial consortium for treatment of recurrent clostridioides difficile infection. Nat Med. 2025;31(1):223–34. 10.1038/s41591-024-03337-4. 39747680 10.1038/s41591-024-03337-4

[467] Quiroga-Centeno AC, Atanasova K, Ebert MP, Thomann AK, Reindl W. Emerging microbiome-directed therapies in inflammatory bowel disease: beyond diet modification and FMT. Semin Immunopathol. 2025;47(1):42. 10.1007/s00281-025-01066-5. 41288714 10.1007/s00281-025-01066-5 PMC12647200

[468] Furuichi M, Kawaguchi T, Pust MM, Yasuma-Mitobe K, Plichta DR, Hasegawa N, et al. Commensal consortia decolonize enterobacteriaceae via ecological control. Nature. 2024;633(8031):878–86. 10.1038/s41586-024-07960-6. 39294375 10.1038/s41586-024-07960-6 PMC11424487

[469] Murali SK, Mansell TJ. Next generation probiotics: engineering live biotherapeutics. Biotechnol Adv. 2024;72:108336. 10.1016/j.biotechadv.2024.108336. 38432422 10.1016/j.biotechadv.2024.108336

[470] Puurunen MK, Vockley J, Searle SL, Sacharow SJ, Phillips JA, Denney WS, et al. Safety and pharmacodynamics of an engineered E. coli nissle for the treatment of phenylketonuria: a first-in-human phase 1/2a study. Nat Metab. 2021;3(8):1125–32. 10.1038/s42255-021-00430-7. 10.1038/s42255-021-00430-7 34294923

[471] Li N, Yin L, Wang J, Zhang J, Tong Y. Programmable probiotics as next-generation living therapeutics: bridging synthetic biology and precision medicine. Curr Opin Biotechnol. 2025;96:103375. 10.1016/j.copbio.2025.103375. 41187701 10.1016/j.copbio.2025.103375

[472] Xu M, Chen S, Pei H, Hu L, Zhang Y. Engineering bacteriophages for gut health: precision antimicrobials and beyond. J Nanobiotechnol. 2026;24(1):62. 10.1186/s12951-026-04038-5. 10.1186/s12951-026-04038-5 PMC12829040 41566387

[473] Gelsinger DR, Ronda C, Ma J, Kar OB, Edwards M, Huang Y, et al. Metagenomic editing of commensal bacteria in vivo using CRISPR-associated transposases. Science. 2025;390(6774):eadx7604. 10.1126/science.adx7604. 10.1126/science.adx7604 PMC12969935 41231980

[474] Gencay YE, Jasinskytė D, Robert C, Semsey S, Martínez V, Petersen AØ, et al. Engineered phage with antibacterial CRISPR–cas selectively reduce E. coli burden in mice. Nat Biotechnol. 2024;42(2):265–74. 10.1038/s41587-023-01759-y. 10.1038/s41587-023-01759-y PMC10869271 37142704

[475] Ticinesi A, Maggi S, Nouvenne A, Zuliani G, Franceschi C. The gut microbiome and ageing trajectories: mechanisms and clinical implications. Nat Rev Endocrinol. 2026. 10.1038/s41574-026-01236-x. 10.1038/s41574-026-01236-x 41776008

[476] Zhang F, Zhou G, Schewe M, Kulling SE, Ding Y, Mozaffarian D, et al. Dietary urbanization destabilizes host-gut microbiome homeostasis and informs precision nutrition for human health. Cell Metab. 2025;37(11):2128–48. 10.1016/j.cmet.2025.09.013. 41135513 10.1016/j.cmet.2025.09.013

[477] Gilbert JA, Azad MB, Bäckhed F, Blaser MJ, Byndloss M, Chiu CY, et al. Clinical translation of microbiome research. Nat Med. 2025;31(4):1099–113. 10.1038/s41591-025-03615-9. 40217076 10.1038/s41591-025-03615-9

[478] Metwaly A, Kriaa A, Hassani Z, Carraturo F, Druart C, Arnauts K, et al. A consensus statement on establishing causality, therapeutic applications and the use of preclinical models in microbiome research. Nat Rev Gastroenterol Hepatol. 2025;22(5):343–56. 10.1038/s41575-025-01041-3. 40033063 10.1038/s41575-025-01041-3

[479] Lv BM, Quan Y, Zhang HY. Causal inference in microbiome medicine: principles and applications. Trends Microbiol. 2021;29(8):736–46. 10.1016/j.tim.2021.03.015. 33895062 10.1016/j.tim.2021.03.015

[480] Huang X, Yu Y, Tian N, Huang J, Zhang X, Yu R. Human microbiota-associated animal models: a review. Front Cell Infect Microbiol. 2025;15:1644187. 10.3389/fcimb.2025.1644187. 40937436 10.3389/fcimb.2025.1644187 PMC12420304

[481] Armetta J, Li SS, Vaaben TH, Vazquez-Uribe R, Sommer MOA. Metagenome-guided culturomics for the targeted enrichment of gut microbes. Nat Commun. 2025;16(1):663. 10.1038/s41467-024-55668-y. 39809763 10.1038/s41467-024-55668-y PMC11733127

[482] Mirzayi C, Renson A, Genomic Standards Consortium, Massive Analysis And Quality Control Society, Zohra F, Elsafoury S, et al. Reporting guidelines for human microbiome research: the STORMS checklist. Nat Med. 2021;27(11):1885–92. 10.1038/s41591-021-01552-x. 34789871 10.1038/s41591-021-01552-x PMC9105086

[483] Kelliher JM, Mirzayi C, Bordenstein SR, Oliver A, Kellogg CA, Hatcher EL, et al. STREAMS guidelines: standards for technical reporting in environmental and host-associated microbiome studies. Nat Microbiol. 2025;10(12):3059–68. 10.1038/s41564-025-02186-2. 41326814 10.1038/s41564-025-02186-2 PMC13231453

[484] Rodriguez J, Hassani Z, Alves Costa Silva C, Betsou F, Carraturo F, Fasano A, et al. State of the art and the future of microbiome-based biomarkers: a multidisciplinary delphi consensus. Lancet, Microbe. 2025;6(2):100948. 10.1016/j.lanmic.2024.07.011. 10.1016/j.lanmic.2024.07.011 39243797

[485] M VH, Pd C. From microbiome to metabolism: bridging a two-decade translational gap. Cell Metab. 2026;38(1):14–32. 10.1016/j.cmet.2025.10.011. 10.1016/j.cmet.2025.10.011 41237775

[486] Hou S, Yu J, Li Y, Zhao D, Zhang Z. Advances in fecal microbiota transplantation for gut dysbiosis-related diseases. Adv Sci. 2025;12(13):2413197. 10.1002/advs.202413197. 10.1002/advs.202413197 PMC11967859 40013938

[487] Vonaesch P, Garneau JR, Dominguez-Bello MG. From global to local: rethinking the design of probiotic intervention strategies. Trends Microbiol. 2025;S0966–842X(25)00336–1. 10.1016/j.tim.2025.11.009. 10.1016/j.tim.2025.11.009 41350154

[488] Cho MY, Eom JH, Choi EM, Yang SJ, Lee D, Kim YY, et al. Recent advances in therapeutic probiotics: insights from human trials. Clin Microbiol Rev. 2025;38(2):e0024024. 10.1128/cmr.00240-24. 40261032 10.1128/cmr.00240-24 PMC12160572

[489] Zhao L, Xin J, Hu M, Xue C, Dong N. Programmable microbial therapeutics: advances in engineered bacteria for targeted in vivo delivery and precision medicine. J Adv Res. 2025;S2090-1232(25)00823-9. 10.1016/j.jare.2025.10.028. 10.1016/j.jare.2025.10.028 PMC13316436 41135875

[490] You Y, Lu H, Wang Y, Wang B, Chen Q, Shi Y. Probiotic encapsulation strategies for controlled intestinal delivery and microbiome dysbiosis therapy. J Control Release: Off J Control Release Soc. 2025;387:114252. 10.1016/j.jconrel.2025.114252. 10.1016/j.jconrel.2025.114252 40983293

[491] Rodriguez J, Cordaillat-Simmons M, Pot B, Druart C. The regulatory framework for microbiome-based therapies: insights into European regulatory developments. NPJ Biofilms Microbiomes. 2025;11(1):53. 10.1038/s41522-025-00683-0. 40155609 10.1038/s41522-025-00683-0 PMC11953238

[492] Muller E, Shiryan I, Borenstein E. Multi-omic integration of microbiome data for identifying disease-associated modules. Nat Commun. 2024;15(1):2621. 10.1038/s41467-024-46888-3. 38521774 10.1038/s41467-024-46888-3 PMC10960825

[493] Zhang Y, Thomas JP, Korcsmaros T, Gul L. Integrating multi-omics to unravel host-microbiome interactions in inflammatory bowel disease. Cell Rep, Med. 2024;5(9):101738. 10.1016/j.xcrm.2024.101738. 39293401 10.1016/j.xcrm.2024.101738 PMC11525031

[494] Rozera T, Pasolli E, Segata N, Ianiro G. Machine learning and artificial intelligence in the multi-omics approach to gut microbiota. Gastroenterology. 2025;169(3):487–501. 10.1053/j.gastro.2025.02.035. 40118220 10.1053/j.gastro.2025.02.035

[495] Wu X, Zhang T, Zhang T, Park S. The impact of gut microbiome enterotypes on ulcerative colitis: Identifying key bacterial species and revealing species co-occurrence networks using machine learning. Gut Microbes. 2024;16(1):2292254. 10.1080/19490976.2023.2292254. 38117560 10.1080/19490976.2023.2292254 PMC10761161

